# The ontogeny of elements: distinct ontogenetic patterns in the radular tooth mineralization of gastropods

**DOI:** 10.1007/s00114-022-01829-2

**Published:** 2022-12-01

**Authors:** Jan-Ole Brütt, Stanislav N. Gorb, Wencke Krings

**Affiliations:** 1 Department of Behavioral Biology, Institute of Cell and Systems Biology of Animals, Universität Hamburg, Martin-Luther-King-Platz 3, 20146 Hamburg, Germany; 2 Department of Mammalogy and Palaeoanthropology, Leibniz Institute for the Analysis of Biodiversity Change, Martin-Luther-King-Platz 3, 20146 Hamburg, Germany; 3 Department of Functional Morphology and Biomechanics, Zoological Institute, Christian-Albrechts-Universität Zu Kiel, Am Botanischen Garten 1-9, 24118 Kiel, Germany

**Keywords:** Elemental composition, Material properties, Feeding, Biomineralization, Mollusca, Gastropoda

## Abstract

The molluscan phylum is characterized by the radula, used for the gathering and processing of food. This structure can consist of a chitinous membrane with embedded rows of teeth, which show structural, chemical, and biomechanical adaptations to the preferred ingesta. With regard to the chemical composition of teeth, some taxa (Polyplacophora and Patellogastropoda) were extensively studied, and high proportions of incorporated iron, calcium, and silicon were previously reported. However, outside these two groups, there is an immense lack of knowledge about the elemental composition of radular teeth. The here presented work aims at shedding some light on the radular composition by performing energy-dispersive X-ray spectroscopy (EDX) on six non-patelliform gastropod species (*Anentome helena*, *Cornu aspersum*, *Lavigeria nassa*, *Littorina littorea*, *Reymondia horei*, and *Vittina turrita*), with the focus on the ontogeny of the elemental composition. Proportions of elements, which are not part of chitin and other purely organic molecules, were documented for overall 1027 individual teeth of all ontogenetic radular stages, i.e., for the building zone, the maturation zone, and the working zone. We detected that the proportions of these elements increased from the building to the maturation zone. However, from the maturation to the working zone, two general trends are visible: either the proportions of the elements increased or decreased. The latter trend could potentially be explained by the acidic pH of the gastropod saliva, which awaits further investigations.

**Supplementary Information:**

The online version contains supplementary material available at 10.1007/s00114-022-01829-2.

## Introduction

Food gathering and processing in most molluscs is enabled by the radula, a thin chitinous membrane with transversal and longitudinal rows of embedded teeth. Each tooth serves as an actual interface between the organism and its ingesta (food, minerals, biofilms, plants, and feeding substrates such as stone or sand) and becomes worn during foraging actions (Runham and Thornton 1967; Shaw et al. 2010; Krings and Gorb 2021a; Krings et al. 2021a). As consequence, teeth and membranes are continuously produced by under and overlain epithelia in the posterior “building zone” or “radular sac” and become maturated in the “maturation zone” before they enter the anterior “working zone,” where teeth actually interact while feeding (e.g., Runham 1963; Runham and Isarankura 1966; Mackenstedt and Märkel 2001).

Adaptations to trophic preferences have been previously reported for (1) the general tooth morphology (e.g., Crampton 1977; Steneck and Watling 1982; Jensen 1997; Nishi and Kohn 1999; Duda et al. 2001; Rintelen et al. 2004; Ekimova et al. 2019; Krings 2020; Krings et al. 2020b, 2021a, 2021b, 2021c; Mikhlina et al. 2020; also in the tooth anchorage with the membrane: Krings et al. 2020a), (2) the arrangement of teeth within the membrane and the resulting tooth-tooth interactions (Solem 1972; Hickman 1980, 1984; Morris and Hickman 1981; Padilla 2003; Herrera et al. 2015; Krings et al. 2020b, 2021d, 2021e, 2021f), and (3) tooth material properties (e.g., hardness and elasticity) (Lu and Barber 2012; Grunenfelder et al. 2014; Barber et al. 2015; Herrera et al. 2015; Ukmar-Godec et al. 2017; Krings et al. 2019a, 2021e; Krings 2020; Pohl et al. 2020; Gorb and Krings 2021; Stegbauer et al. 2021). The properties measured can either result from the architecture of the organic components (matrix of alpha chitin with associated proteins), e.g., fiber orientation and/or density, the folding or bounding conditions of chitin, and/or the incorporation of inorganic components as e.g., iron, silicon, and calcium (e.g., Weaver et al. 2010; Wang et al. 2013; Grunenfelder et al. 2014; Herrera et al. 2015; Ukmar-Godec 2016; Pohl et al. 2020; Stegbauer et al. 2021; Krings et al. 2022a, 2022b; for reviews, see Brooker and Shaw 2012; Faivre and Ukmar-Godec 2015; Joester and Brooker 2016; Kisailus and Nemoto 2018; Moura and Unterlass 2020). In some species, possessing very hard and stiff teeth, i.e., Patellogastropoda and Polyplacophora, very high proportions of silicon and iron are incorporated in the tooth cusps, which is an adaptation to loosening algae from stones (e.g., Lu and Barber 2012; Grunenfelder et al. 2014; Barber et al. 2015; Herrera et al. 2015; Ukmar-Godec et al. 2017; Pohl et al. 2020; Stegbauer et al. 2021; Krings et al. 2022a). In many molluscan taxa, however, teeth seem to be rather more chitinous and less mineralized, even though some of these species, e.g., the paludomid gastropods from Lake Tanganyika and surrounding river systems, also forage on algae attached to rocks (Krings et al. 2022b).

Only very few studies on the composition of radular teeth outside the limpet and chiton realm were conducted (e.g., Troschel 1863; Sollas 1907; Jones et al. 1935; Tillier and Cuif 1986; Macey et al. 1997; Cruz et al. 1998; Krings et al. 2022b). Thus, the inorganic content of the larger, species-rich molluscan orders (e.g., Heterobranchia, Caenogastropoda, Neritimorpha, etc.) remains enigmatic. To shed some light on the elemental composition, we performed elemental analyses using energy disperse X-ray spectroscopy (EDX, EDS) on radulae from six non-patelliform gastropod species (the caenogastropods *Anentome helena*, *Lavigeria nassa*, *Littorina littorea*, and *Reymondia horei*; the heterobranch *Cornu aspersum*; and the neritimorph *Vittina turrita*), overall, on 1027 individual teeth. The data on the elemental composition of the working zone was published before (Krings et al. 2022b), and here we present data on the ontogeny of the elemental composition of the building and maturation zones. In general, we detected that the proportions of the elements that are not part of chitin and other purely organic molecules increased from the building to the maturation zone. However, we detected two patterns from the maturation to the working zone: either the elemental proportions increased or decreased.

## Materials and methods

### Species and specimens

The results of the elemental analyses presented in this work were obtained from the same specimens studied in our previous paper, in which we described the elemental composition of the radular working zones (Krings et al. 2022b). Individuals of *Anentome helena* (von dem Busch 1847) (Caenogastropoda), *Cornu aspersum* (Müller 1774) (Heterobranchia), and *Vittina turrita* (Gmelin 1791) (Neritimorpha) were bought from online pet shops in 2018, 2019, and 2020. *Littorina littorea* (Linnaeus 1758) (Caenogastropoda) was collected at the North Sea, at Husum, Germany, in autumn 2019. *Lavigeria nassa* (Woodward 1859) and *Reymondia horei* (Smith 1880) (both Caenogastropoda) were collected in Lake Tanganyika; *L. nassa* in Zambia (08°29′23″S, 30°28′46″E) on 09/09/2016 and *R. horei* in Tanzania (Kigoma) on 02/26/1995. Specimens are either inventoried at the Museum für Naturkunde Berlin (ZMB) or the Zoological Museum Hamburg (ZMH), which is now part of the Leibniz Institute for the Analysis of Biodiversity Change (LIB): *L. nassa*, ZMH 119369/999, *R. horei*, ZMB 220.147, *V. turrita*, ZMH 154753, *L. littorea*, ZMH 154633, *C. aspersum*, ZMH 150005, and *A. helena* (ZMH 122792). All specimens were initially preserved in 70% ethanol.

*A. helena* forages on other gastropods, fish eggs, shrimps, and carrion (Bogan and Hanneman 2013; Strong et al. 2017), *L. littorea* on fleshy macroalgae from rocks (Watson and Norton 1985; Imrieet al. 1990; Olsson et al. 2007; Lauzon-Guay and Scheibling 2009), *L. nassa* on algae from rocks (Bourguignat 1885, 1888; Moore 1903; Leloup 1953; Brown 1994; personal comment from the collector Heinz Büscher), *R. horei* on algae from rocks (Bourguignat 1885, 1888; Coulter 1991; Bandel 1997; West et al. 2003; personal comment from the collector Heinz Büscher), *V. turrita* on algae from solid substrates, but also porous ingesta (Eichhorst 2016), and *C. aspersum* on various plant types (www.cabi.org/isc/datasheet/26821).

Overall, we studied four adult individuals of similar shell size per species. For this purpose, specimens were dissected, and each radula was extracted and carefully freed from surrounding tissues by tweezers. Then, the radulae were cleaned in an ultrasonic bath for 2–20 s, and each radular membrane was attached to one glass object slide with double-sided adhesive tape.

### Documentation and categorization of the radular zones

All radulae were first documented with the Keyence Digital Microscope VHX-7000 (KEYENCE, Neu-Isenburg, Germany), and radular zones were defined. The building zone (zone 1) is always the most posterior radular area, with fragile membranes and teeth that are often curled up, covered by secreting epithelia, and densely packed. The working zone (usually zone 3; in *Vittina turrita* and *Littorina littorea*, it is defined as zone 4) is always the anterior area, used for feeding and not covered by epithelia. Zone 2, the maturation zone, is situated between these two zones and is also covered by secreting epithelia. For *V. turrita* and *L. littorea,* we defined two maturation zones between the building and working zone (zones 2 and 3), as their radulae are quite long. For the completeness of this paper, we included some scanning electron microscopy (SEM) images from previous studies, where we described the radular morphology of the species studied in detail (Krings et al. 2019b, 2021a, 2021b, 2021d, 2021e; Scheel et al. 2020; Krings 2020; Krings and Gorb 2021b).

### Elemental analysis

After documentation of the external micromorphology of radulae using SEM, two radulae per species were chosen for EDX analysis (these are the same specimens as previously analyzed in Krings et al. 2022b). Here, the radulae were first removed from the adhesive tape with 70% ethanol. Then, radulae were again taped with double-sided adhesive tape to glass object slides, but now the outer teeth of one side were attached (see Krings et al. 2022a, 2022b for details). Each radula was air-dried and surrounded by a small metallic ring, which was filled with epoxy resin (RECKLI EPOXI WST, RECKLI GmbH, Herne, Germany). After polymerization at room temperature, the object slide and tape were removed. Samples were polished with sandpapers of different roughness until sections of the outer teeth were on display. Then they have smoothed with aluminum oxide polishing powder suspension of 0.3-μm grain size (PRESI GmbH, Hagen, Germany) on a polishing machine (Minitech 233/333, PRESI GmbH, Hagen, Germany). Afterward, they were cleaned in an ultrasonic bath for 5 min and coated with platinum (Pt, 5 nm-thick layers). The elemental composition of the largest possible area per tooth (point measurements were performed, not elemental mappings) was examined employing the SEM Zeiss LEO 1525 (One Zeiss Drive, Thornwood, NY) equipped with an Octane Silicon Drift Detector (micro analyses system TEAM, EDAX Inc., NJ, USA) always using an acceleration voltage of 20 keV and the same device settings (e.g., exposure time, the opening of the lens, etc.) as in previous studies on radular elemental composition (Krings et al. 2022a, 2022b, 2022c). Before the analysis of a sample, the device was always calibrated with copper (Cu).

The proportions of H (hydrogen), C (carbon), N (nitrogen), O (oxygen), Pt (platinum), Al (aluminum), Ca (calcium), Na (sodium), Mg (magnesium), Si (silicon), P (phosphorus), S (sulfur), Cl (chlorine), K (potassium), F (fluorine), Cu (copper), and Fe (iron) were measured, if detected. The atomic ratios (atomic %) were received with two positions after the decimal point; lower proportions were not detectable with this method. We did not discuss the following elements, as they are either the elemental basis of chitin/proteins (H, C, N, and O), the coating (Pt), or the polishing powder (Al and O).

**Fig. 1:**
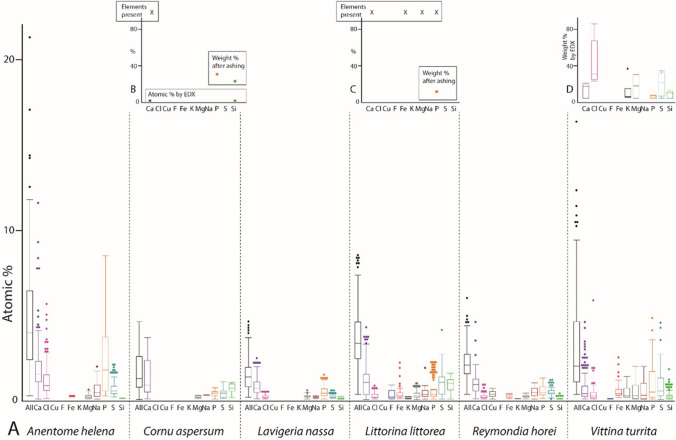
**A** Proportions of elements, in atomic percent, per mollusc species. For values and quantity of measurements, see Supplementary Table 2. **B**–**D** Summary of previous studies on the elemental composition of **B** *Cornu aspersum* (element detected [X], and weight % after ashing from Sollas 1907; atomic % by EDX from Krings et al. 2019a), **C** *Littorina littorea* (element present [X] and weight % after ashing from Sollas 1907), and **D** the neritid *Nerita atramentosa* (weight % by EDX from Macey et al. 1997)

After analysis of the outer teeth, each sample was again polished and smoothed until the next tooth type or longitudinal row was on display; cleaning procedures and EDX analyses were again performed. Every step was repeated until all target teeth were measured. In the past study (Krings et al. 2022b), we already presented the results of the radular working zone. The results from all immature radular zones are new. Overall, we performed and analyzed 1027 individual point measurements (one point measurement per tooth, thus 1027 teeth were studied) from 12 specimens (Fig. 1).

### Statistical analyses

With JMP Pro, Version 14 (SAS Institute Inc., Cary, NC, 1989–2007), mean values and standard deviations were calculated for EDX results. We summed the values from both specimens per species because the elemental compositions of individual radulae did not differ significantly in most cases (see Supplementary Fig. S1 and Supplementary Table S1). Shapiro–Wilk *W*-tests for testing normality were conducted. When the data was not normally distributed, a Kruskal–Wallis test was carried out. Pairwise comparisons were performed with the Wilcoxon method.

## Results

### Radular morphology and types

**Fig. 2:**
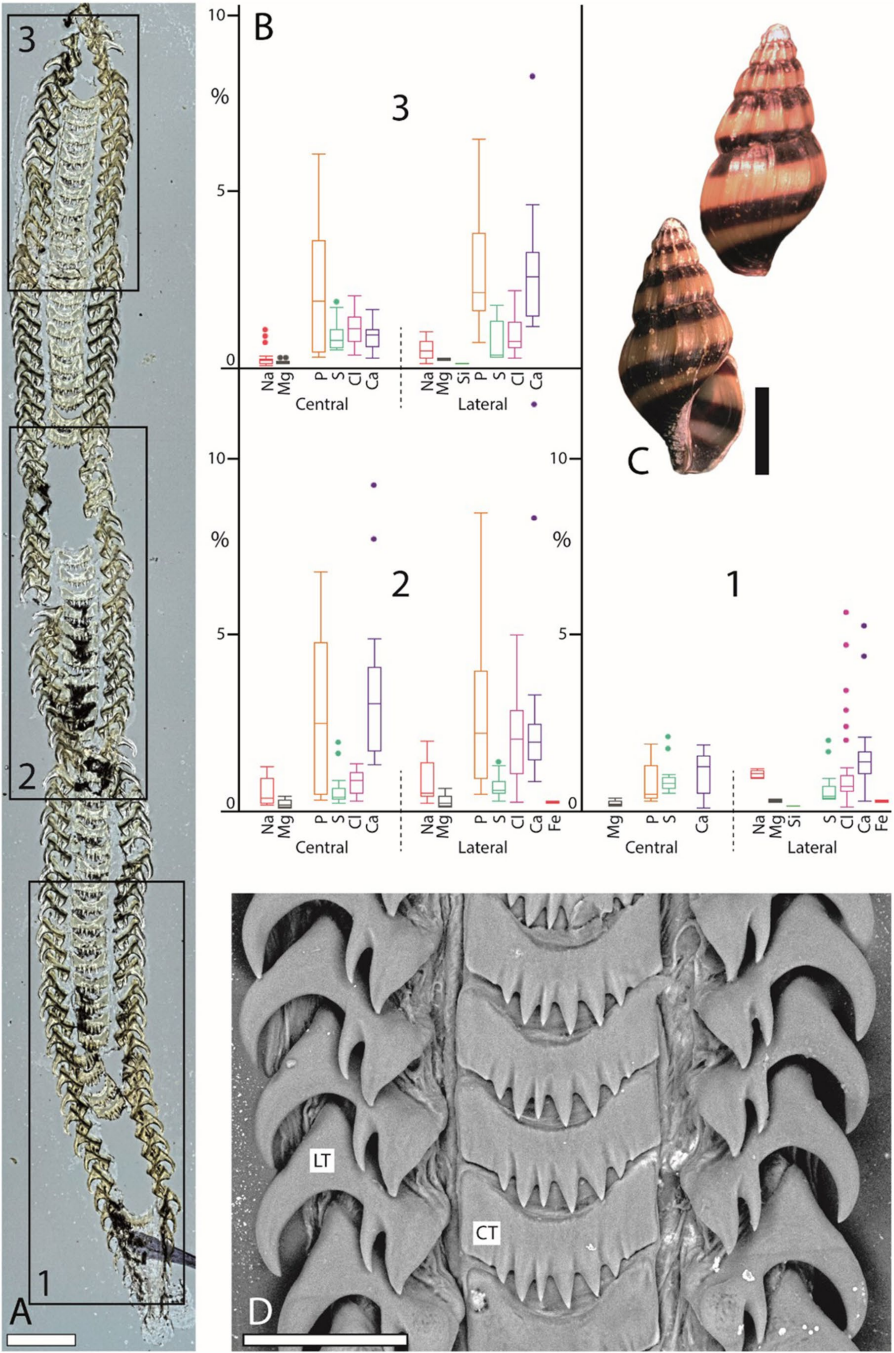
Summarized results for *Anentome helena* (Caenogastropoda). **A** Light microscopy image of the whole radula with highlighted distinct ontogenetic zones (zone 1 = building zone, zone 2 = maturation zone, and zone 3 = working zone). **B** Proportions of individual elements in atomic percent per zone and tooth type (for means, SD, N, and statistics, see Supplementary Tables 2 and 3). **C** Shell habitus from one analyzed individual (taken from Krings et al. 2022b). **D** SEM image of unused teeth of one individual (taken from Krings et al. 2022b). Scale bars: A, 200 µm; C, 5 mm; D, 40 µm. CT, central tooth; LT, lateral tooth. Figures 2, 3, 4, 5, 6, and 7 are at different scales; for comparison at the same scale, see Supplementary Figs. 2, 3, and 4)

**Fig. 3:**
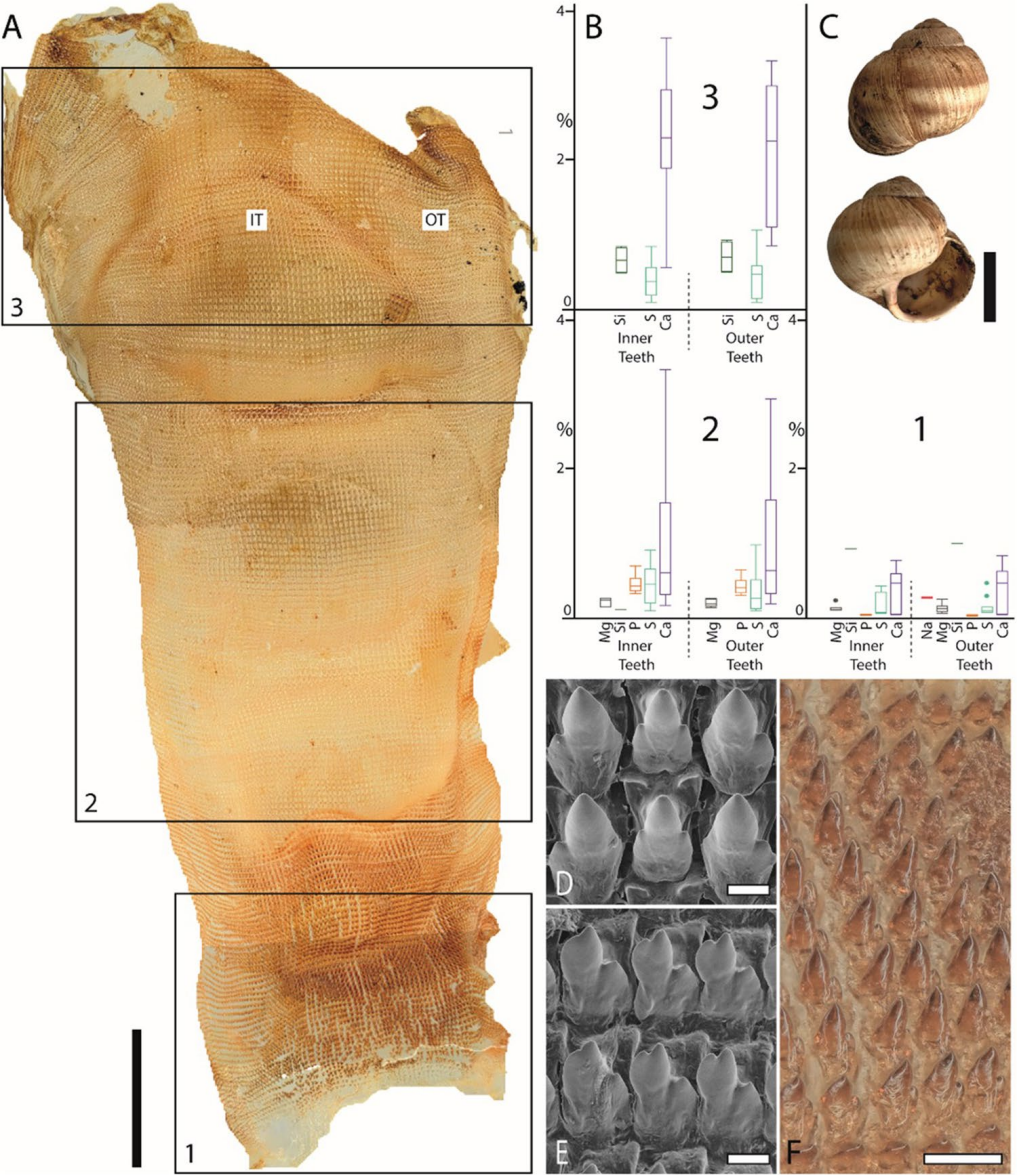
Summarized results for *Cornu aspersum* (Heterobranchia). **A**. Light microscopy image of the whole radula with highlighted distinct ontogenetic zones (zone 1 = building zone, zone 2 = maturation zone, and zone 3 = working zone). **B** Proportions of individual elements in atomic percent per zone and tooth type (for means, SD, N, and statistics, see Supplementary Tables 2 and 3). **C** Shell habitus from one analyzed individual (taken from Krings et al. 2022b). D, E. SEM images of unused teeth of one individual, **D** central and lateral teeth, and **E** marginal teeth (taken from Krings et al. 2019b). **F** Light microscopy image of marginal teeth. Scale bars: A, 1 mm; C, 15 mm; D, E, 10 µm; F, 40 µm. IT, inner teeth; OT, outer teeth. Figures 2, 3, 4, 5, 6, and 7 are at different scales; for comparison at the same scale, see Supplementary Figs. 2, 3, and 4)

**Fig. 4:**
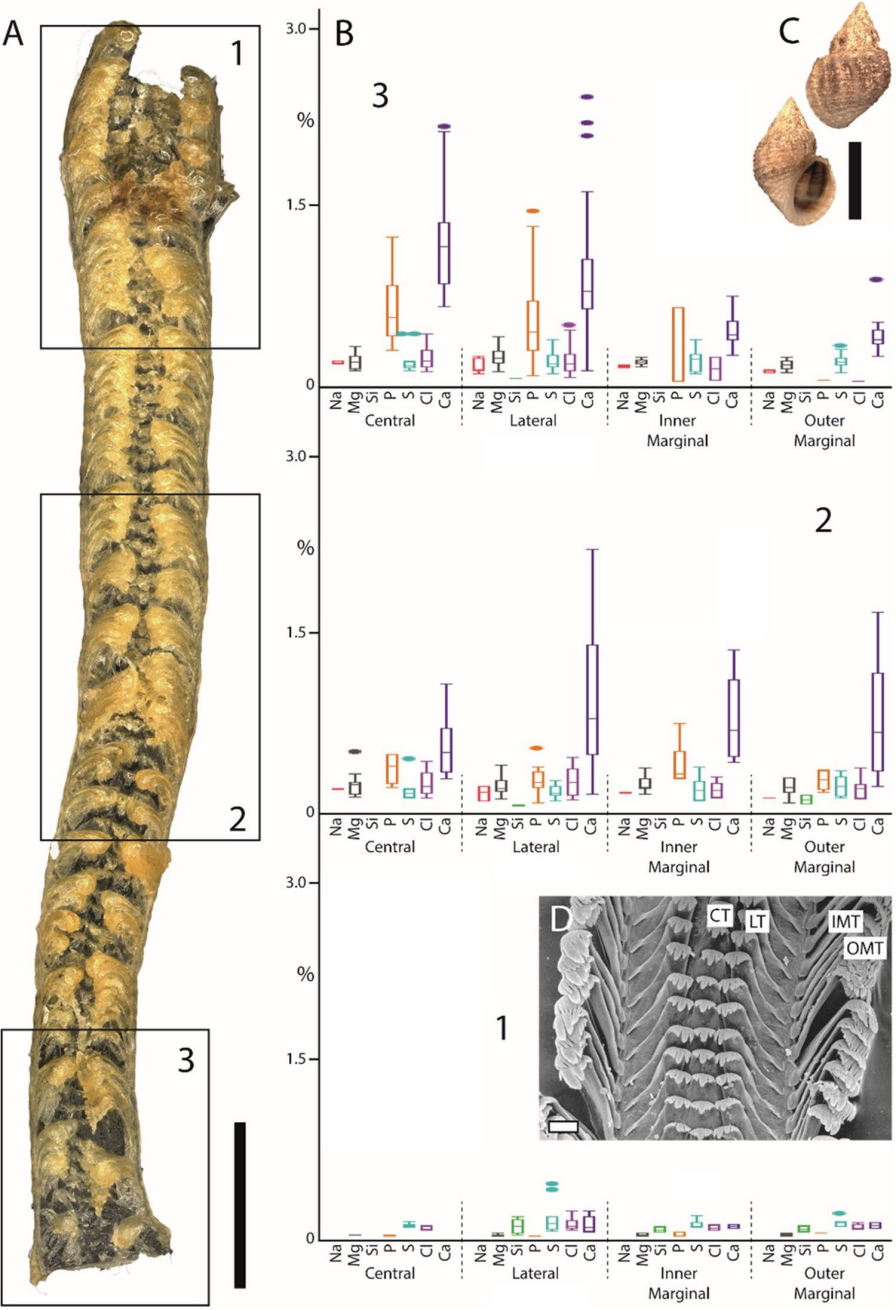
Summarized results for *Lavigeria nassa* (Caenogastropoda). **A** Light microscopy image of the whole radula with highlighted distinct ontogenetic zones (zone 1 = building zone, zone 2 = maturation zone, and zone 3 = working zone). **B** Proportions of individual elements in atomic percent per zone and tooth type (for means, SD, N, and statistics, see Supplementary Tables 2 and 3). **C** Shell habitus from one analyzed individual (taken from Krings et al. 2022b). **D** SEM image of unused teeth of one individual (taken from Krings et al. 2021e). Scale bars: A, 400 µm; C, 20 mm; D, 30 µm. CT, central tooth; IMT, inner marginal tooth; LT, lateral tooth; OMT, outer marginal tooth. Figures 2, 3, 4, 5, 6, and 7 are at different scales; for comparison at the same scale, see Supplementary Figs. 2, 3, and 4)

**Fig. 5:**
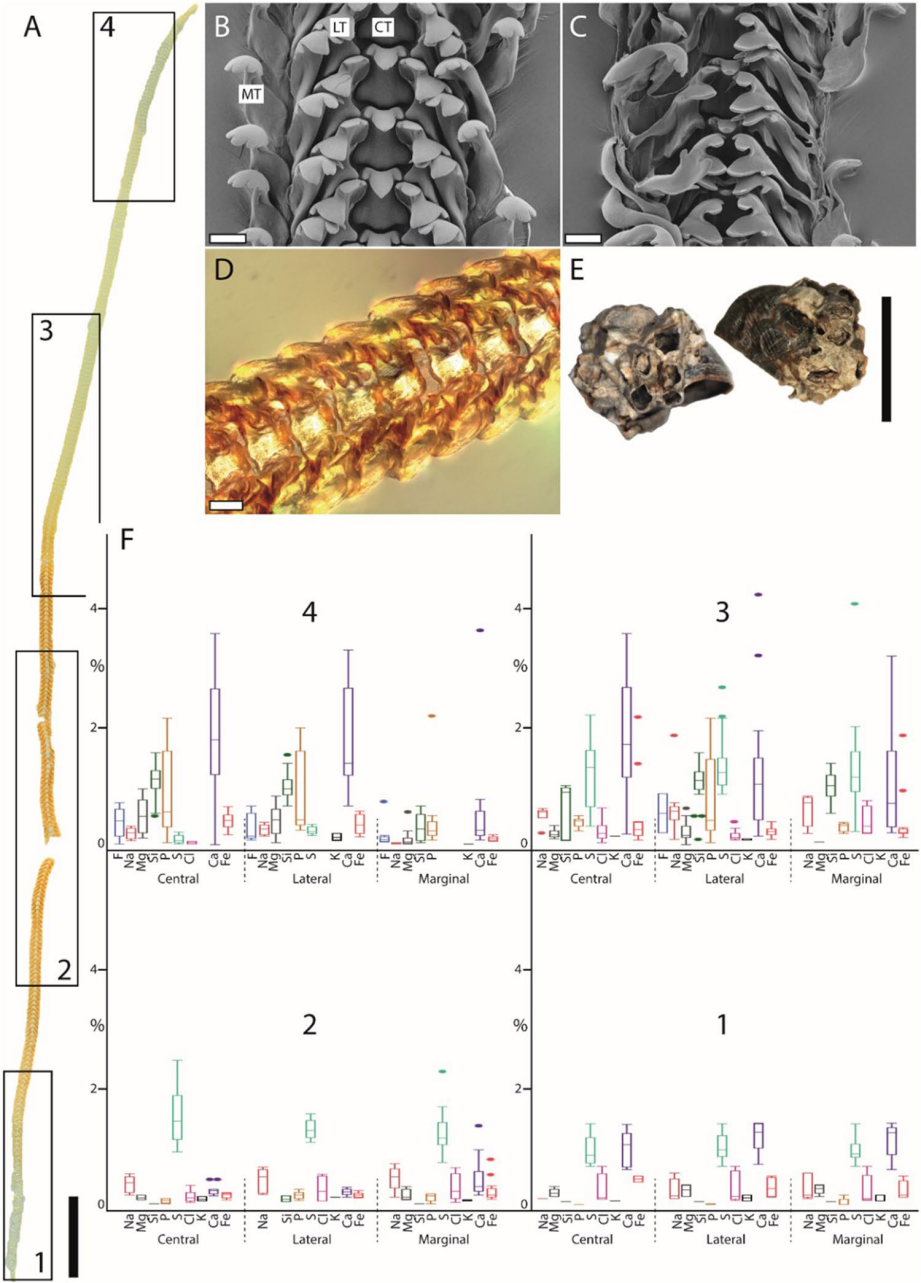
Summarized results for *Littorina littorea* (Caenogastropoda). **A** Light microscopy image of the whole radula with highlighted distinct ontogenetic zones (zone 1 = building zone, zone 2 = maturation zone 1, zone 3 = maturation zone 2, and zone 4 = working zone). **B, C** SEM images of unused teeth of one individual (taken from Scheel et al. 2020), **B** unused teeth from the posterior working zone, and **C** immature teeth from the building zone. **D** Light microscopy image of zone 3. **E** Shell habitus from one analyzed individual (taken from Krings et al. 2022b). **F** Proportions of individual elements in atomic percent per zone and tooth type (for means, SD, N, and statistics, see Supplementary Tables 2 and 3). Scale bars: A, 1 mm; B–C, 80 µm; D, 40 µm; E, 12 mm. CT, central tooth; LT, lateral tooth; MT, marginal tooth. Figures 2, 3, 4, 5, 6, and 7 are at different scales; for comparison at the same scale, see Supplementary Figs. 2, 3, and 4)

**Fig. 6:**
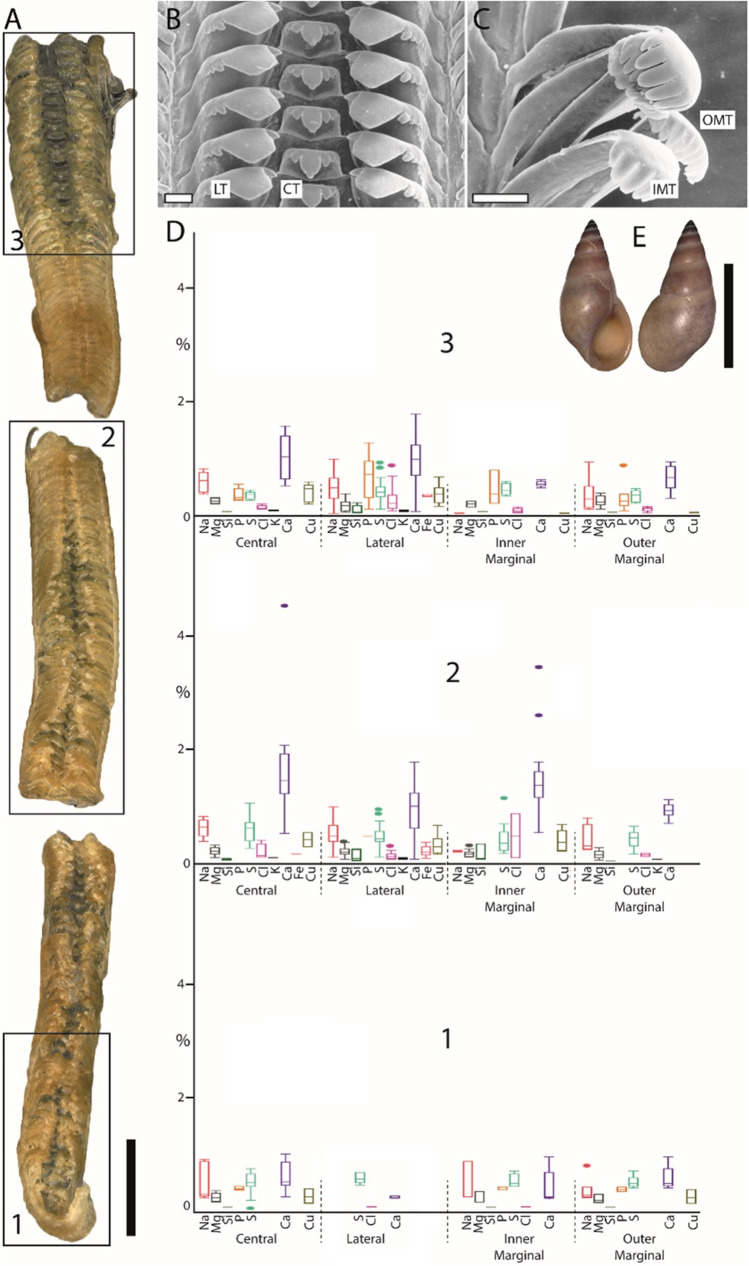
Summarized results for *Reymondia horei* (Caenogastropoda). **A** Light microscopy image of the whole radula with highlighted distinct ontogenetic zones (zone 1 = building zone, zone 2 = maturation zone, and zone 3 = working zone). B, C SEM images of unused teeth of one individual (taken from Krings et al. 2021e), **B** central and lateral teeth, and **C** marginal teeth. **D** Proportions of individual elements in atomic percent per zone and tooth type (for means, SD, N, and statistics, see Supplementary Tables 2 and 3). **E** Shell habitus from one analyzed individual (taken from Krings et al. 2022b). Scale bars: A, 200 µm; B–C, 30 µm; E, 10 mm. CT, central tooth; IMT, inner marginal tooth; LT, lateral tooth; OMT, outer marginal tooth. Figures 2, 3, 4, 5, 6, and 7 are at different scales, for comparison at the same scale, see Supplementary Figs. 2, 3, and 4)

**Fig. 7:**
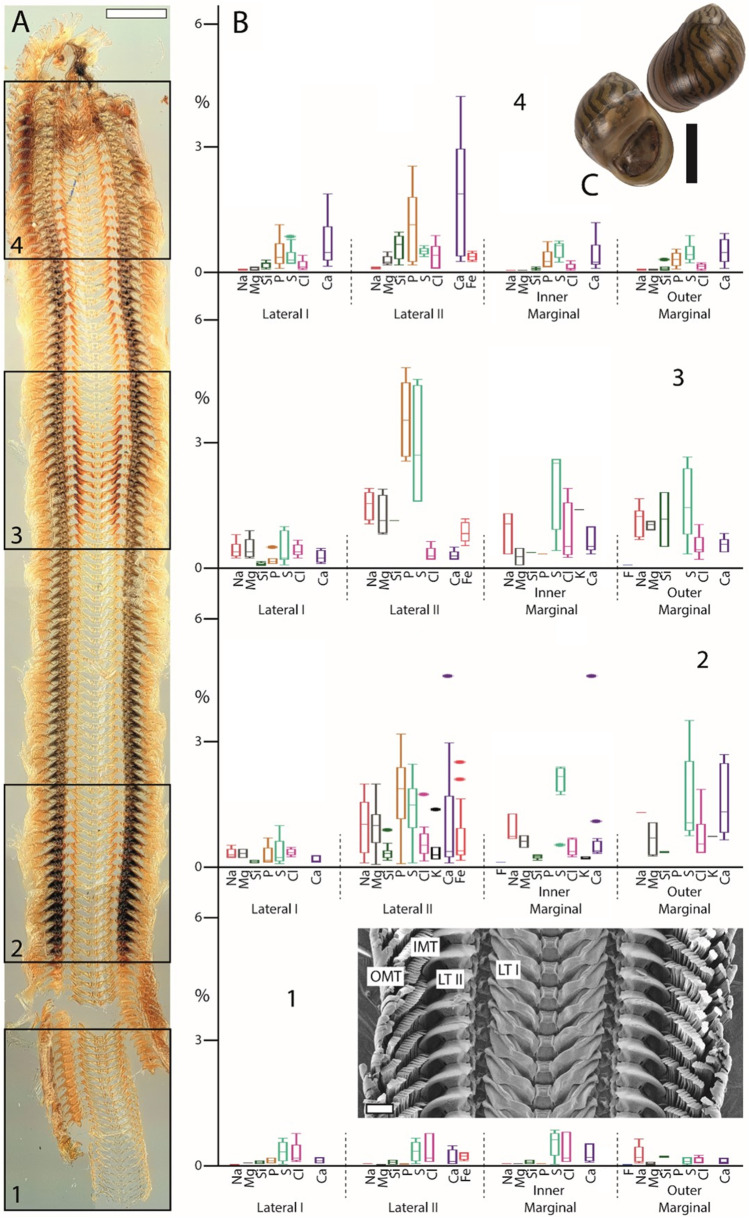
Summarized results for *Vittina turrita* (Neritimorpha). **A** Light microscopy image of the whole radula with highlighted distinct ontogenetic zones (zone 1 = building zone, zone 2 = maturation zone 1, zone 3 = maturation zone 2, and zone 4 = working zone). **B** Proportions of individual elements in atomic percent per zone and tooth type (for means, SD, N, and statistics, see Supplementary Tables 2 and 3). **C** Shell habitus from one analyzed individual (taken from Scheel et al. 2020). **D** SEM image of unused teeth of one specimen (taken from Krings et al. 2021a). Scale bars: A, 500 µm, C = 30 mm, D = 100 µm. IMT, inner marginal tooth; LT I, lateral tooth I; LT II, lateral tooth II; OMT, outer marginal tooth. Figures 2, 2, 4, 5, 6, and 7 are at different scales; for comparison at same scale, see Supplementary Figs. 2, 3, and 4)

The caenogastropod *Anentome helena* (Fig. 2) possesses a stenoglossan radula with one central tooth flanked to each side by one lateral tooth. The heterobranch *Cornu aspersum* (Fig. 3) has an isodont radula with one central tooth and 60–70 lateral and ~ 80 marginal teeth. The caenogastropods *Lavigeria nassa* (Fig. 4), *Littorina littorea* (Fig. 5), and *Reymondia horei* (Fig. 6) have a taenioglossan radula with one central tooth flanked on both sides by one lateral and two marginals. The neritimorph *Vittina turrita* (Fig. 7) possesses a rhipidoglossan type of radula (special type “neritimorph”) with one central tooth, followed by two lateral teeth (lateral tooth I and II) and numerous marginal teeth (~ 40 teeth) on each side.

### Whole radulae

In all specimens studied, we found Ca, Mg, Na, P, S, and Si (Fig. 1). Cl was determined in most species but not in *Cornu aspersum*. Fe was detected in *Anentome helena*, *Littorina littorea*, *Reymondia horei*, and *Vittina turrita*, but not in *C. aspersum* and *Lavigeria nassa*. K was present in *L. littorea*, *R. horei*, and *V. turrita*, Cu only in *R. horei*, and F only in *L. littorea*.

The highest proportion of all elements in the whole radula was found in *A. helena,* followed by *L. littorea, V. turrita*, *R. horei*, *C. aspersum*, and finally, *L. nassa* (see Fig. 1)*.*

### Ontogenetic zones

#### Elements detected

**Fig. 8:**
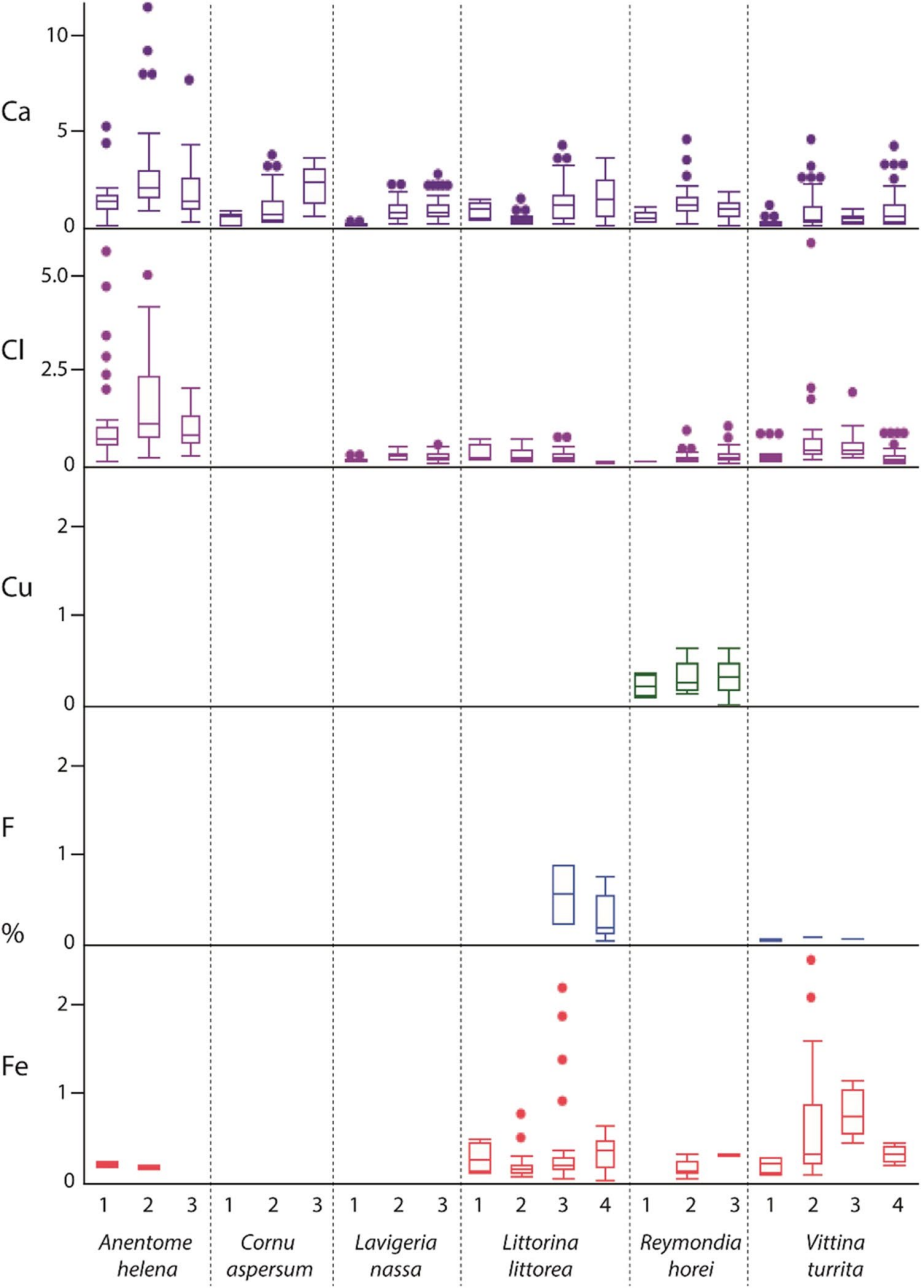
Proportions of individual elements, in atomic percent, per mollusc species and radular zone (zone 1 = building zone, zone 3 in most species, and zone 4 in *Littorina littorea* and *Vittina turrita* = working zone). For values and quantity of measurements, see Supplementary Table 2

**Fig. 9:**
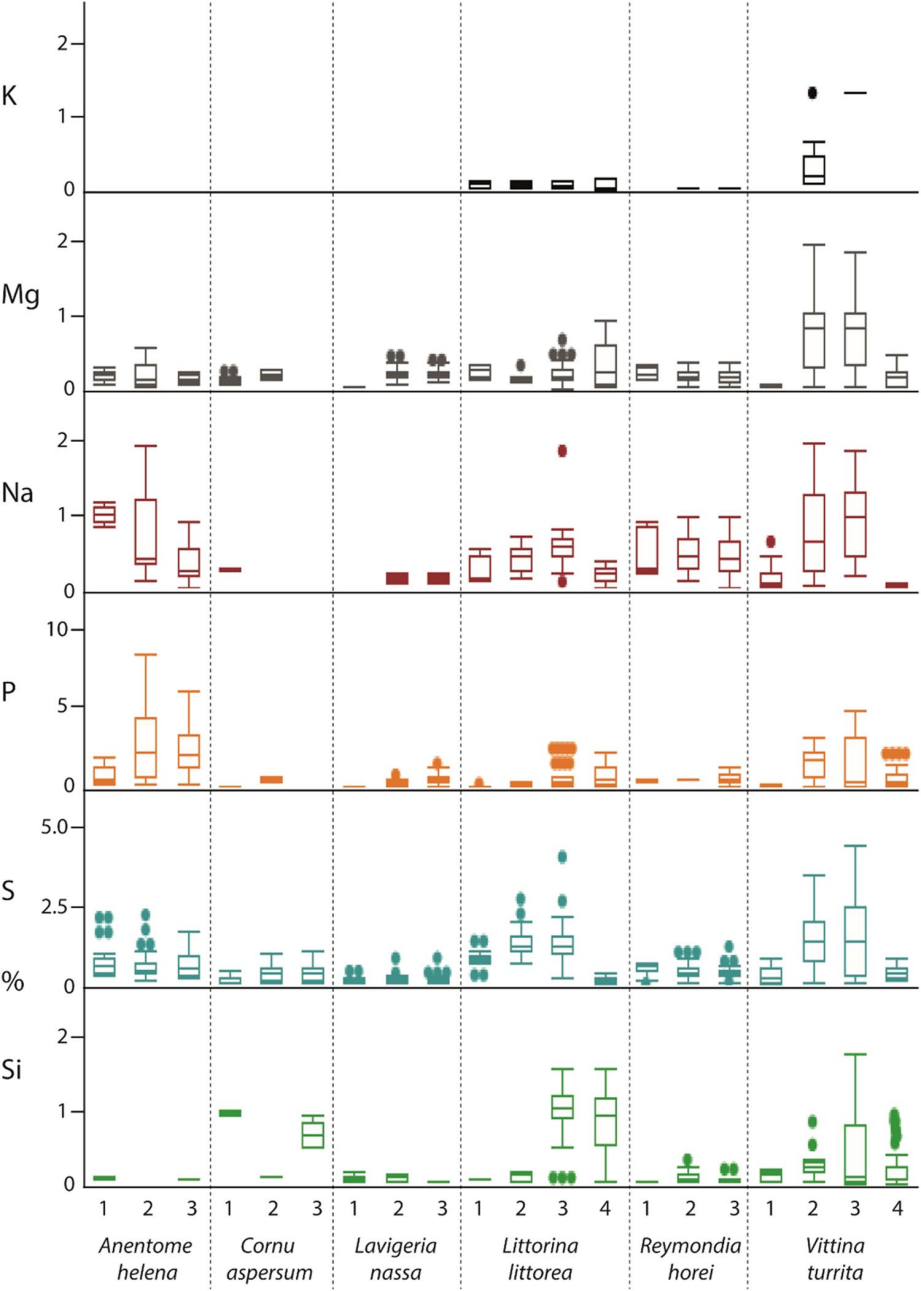
Proportions of individual elements, in atomic percent, per mollusc species and radular zone (zone 1 = building zone, zone 3 in most species, and zone 4 in *Littorina littorea* and *Vittina turrita* = working zone). For values and quantity of measurements, see Supplementary Table 2

In most cases, the individual elements studied are already present in the building zone (zone 1) (Figs. 8 and 9). However, in zone 1, the following elements were not detected: F in *Littorina littorea*, Fe and K in *Reymondia horei*, K in *Vittina turrita*, and Na in *Lavigeria nassa*. They seem to be first secreted in zone 2.

In general, if an element was detected in a species, it was always present in the maturation zone, except for Na in *Cornu aspersum*, as it was only abundant in zone 1, and Si in *Anentome helena*, as it was not detected in zone 2 (Figs. 8 and 9). In most cases, the presence of the individual elements persisted in the working zone. However, no Fe was determined for this zone in *A. helena*, no K in *V. turrita*, and no Mg and P in *C. aspersum*.

#### Elemental proportion comparison between zones

**Fig. 10:**
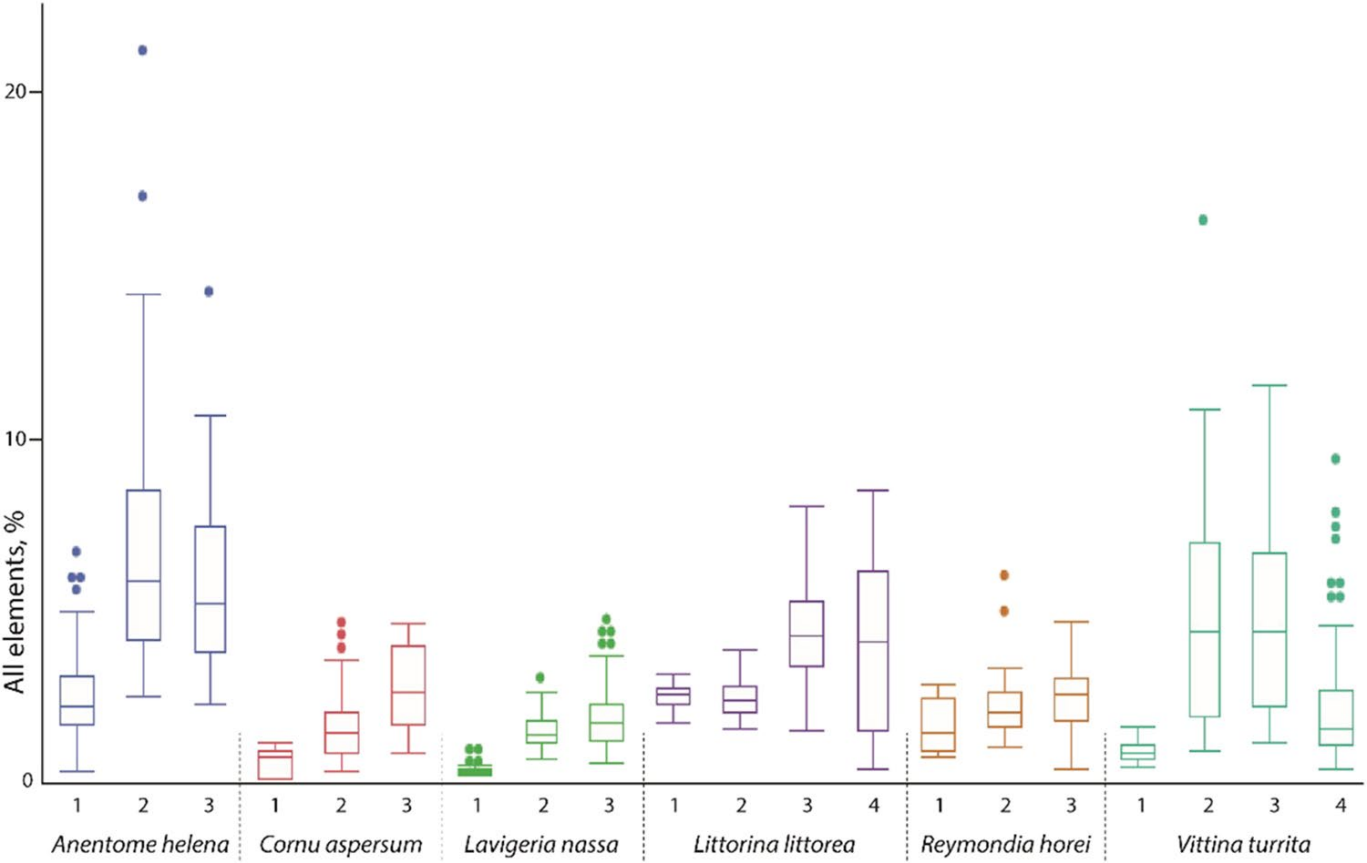
Proportions of all elements, in atomic percent, per mollusc species and radular zone (zone 1 = building zone, zone 3 in most species, and zone 4 in *Littorina littorea* and *Vittina turrita* = working zone). For values and quantity of measurements, see Supplementary Table 2

In general, we detected two contrary trends regarding the chemical composition of the distinct radular ontogenetic zones (Fig. 10). In all studied radulae, the elemental proportions increased from the posterior building zone (zone 1) to the maturation zone (zone 2 in *Anentome helena*, *Cornu aspersum*, *Lavigeria nassa*, and *Reymondia horei*; zones 2 and 3 in *Littorina littorea* and *Vittina turrita*). Then either the proportions increased further (pattern A) from the maturation to the working zone or they decreased (pattern B). Pattern A was detected in *C. aspersum*, *L. nassa*, and *R. horei*. Pattern B was found in *A. helena* and *V. turrita*. In *L. littorea,* we detected only a slight decrease. These patterns are, in general, detectable for every element studied, except for Si in *A. helena* and *C. aspersum*.

### Comparison between species

In *Anentome helena*, the lateral teeth contained more elements than the central ones (Fig. 2). Additionally, Fe was detected in the building and maturation zones but absent in the working zone. In *Cornu aspersum,* however, the inner teeth contain the highest proportions of elements; Ca was the most abundant element (Fig. 3). For *Lavigeria nassa*, the highest contents were detected for the centrals, followed by the laterals, the inner, and finally the outer marginals. The proportions increased strongly in the maturation zone (Fig. 4). *Littorina littorea* possesses the longest radula studied, and its elemental content increased gradually from the building zone to maturation zone 3 (Fig. 5). Here, also the central teeth have the highest proportions of elements, followed by the laterals, and finally the marginals. *Reymondia horei* is the only species analyzed that contained Cu (Fig. 6). In *Vittina turrita,* very small proportions of P, S, and Ca are present in the working zone (Fig. 7).

## Discussion

### Previous studies on the elemental composition

Most previous analyses were either focused on the presence or abundance of specific elements in the whole radula, determined by, e.g., ashing, acid treatment or mass spectrometry (for non-patelliform gastropods, see Troschel 1863; Sollas 1907; Jones et al. 1935; for Patellogastropoda, see Troschel 1863; Sollas 1907; Jones et al. 1935; Shaw et al. 2008; for Polyplacophora, see Jones et al. 1935; Shaw et al. 2008; Emmanuel et al. 2014) or on the detailed composition of the dominant lateral teeth in the working zone of Polyplacophora (van der Wal 1989; Evans et al. 1992; Lee et al. 2003; Brooker et al. 2006; Weaver et al. 2010; Gordon and Joester 2011; Grunenfelder et al. 2014; Herrera et al. 2015; Pohl et al. 2020; Stegbauer et al. 2021; for reviews, see Brooker and Shaw 2012; Faivre and Ukmar-Godec 2015; Joester and Brooker 2016; Kisailus and Nemoto 2018; Moura and Unterlass 2020).

By EDX analyses, the proportions of individual elements in a defined area can be identified, but not the specific bonding and structure of molecules. However, from the presence of the elements and by comparing our results with past studies on radular chemistry, we previously proposed that the following elements are potentially part of the following molecules or minerals (see Krings et al. 2022b). Elements of types 1–5 probably stiffen and harden the chitinous radular teeth.

- Type 1: Characterized by the presence of Fe. Potentially present in the form of magnetite, as documented in polyplacophoran, or goethite, found in limpets (e.g., Lowenstam 1962, Kirschvink and Lowenstam 1979, Lowenstam and Weiner 1989, Huang et al. 1992, Han et al. 2011, Wang et al. 2013, Ukmar-Godec 2016, Nemoto et al. 2019, and McCoey et al. 2020). Fe was detected in the caenogastropods *Anentome helena*, *Littorina littorea*, *Reymondia horei*, and the neritimorph *Vittina turrita—*but only as traces. As it was, however, quite consistently identified in the teeth of *L. littorea* and *V. turrita*, it might play a (small) role in increasing the stiffness or hardness of their teeth—in contrast to the Polyplacophora and Patellogastropoda with very high proportions. The results from our analyses indicate that the epithelium surrounding zone 1 already secretes Fe in *A. helena*, *L. littorea*, and *V. turrita*, whereas in *R. horei*, Fe is incorporated first in zone 2. In the heterobranch *Cornu aspersum* and the caenogastropod *Lavigeria nassa*, it was not detected. Potentially, the incorporation of Fe is ancestral to the Gastropoda, especially since Patellogastropoda contains high proportions and were reduced during evolution; this statement is, however, rather speculative and requires a broader taxon sampling.
- Type 2: Characterized by the presence of Mg and Ca. Elements are potentially involved in the protein packing, increasing the density of chitin fibers and thus the material stiffness, as documented in limpet teeth (Ukmar-Godec et al. 2017). Mg and Ca were detected in all species—thus this type seems to be ancestral to all Gastropoda*.* For the heterobranch *C. aspersum*, we did not detect Mg in the working zone, which could indicate that this element might be lost in this species. In all species, Mg and Ca are already present in zone 1, which indicates that here the epithelium already secretes these elements.
- Type 3: Characterized by the presence of Ca, P, Cl, and/or F. These elements (Ca:P:Cl/F) are potentially part of apatite, either fluorapatite, Ca_5_[F|(PO_4_)_3_], or chlorapatite, Ca_5_[Cl|(PO_4_)_3_], as previously described for radular teeth of polyplacophorans (e.g., Lowenstam 1967, Brooker et al. 2001, Brooker and Macey 2001, Brooker et al. 2003, Shaw et al. 2008, and Shaw et al. 2009). Ca in connection with P, Cl, and/or F was determined in *A. helena*, *R. horei*, *V. turrita*, *L. nassa*, and *L. littorea.* For these species, elements are already present in zone 1, which again indicates that the tissues here secrete P, Cl, F, and Ca. For the heterobranch, *C. aspersum*, Ca, and P are also present from zone 1 on; however, we did not find F or Cl, so potentially, these elements are not bonded in form of apatite.
- Type 4: Characterized by the presence of Si. Potentially present in the form of silica, as documented in limpet teeth (e.g., Hua and Li 2007, Faivre and Ukmar-Godec 2015). In all species studied, we detected Si, so potentially the incorporation in the teeth is ancestral. However, it was determined to be present only with small proportions, indicating that this inorganic content might not have a high influence on the mechanical properties of teeth. In every species, we found that Si is already present in zone 1.
- Type 5: Characterized by the presence of Cu. This element was previously reported for cephalopod teeth (Krings et al. 2022b) and is potentially also involved in the hardening of teeth. We here determined Cu in the caenogastropod *Reymondia horei*, present from zone 1 on.
- Type 6: The presence of Na, K, and S can be related to the protein bonding (e.g., Creighton 1997 and Harding 2002; for proteins in radulae, see Nemoto et al. 2012). These elements were also detected in all species from zone 1 on, as chitin is always associated with proteins in Mollusca.

Regarding the ontogenetic changes in the proportions of elements, the dominant lateral teeth of Polyplacophora (Kim et al. 1986; Macey and Brooker 1996; Lee et al. 2000; Brooker and Macey 2001; Brooker et al. 2003) and Patellogastropoda (Runham et al. 1969; Liddiard et al. 2004; Hua and Li 2007) were usually in the focus of research, except for one study on all teeth in the chiton *Lepidochitona cinerea* (Krings et al. 2022a). No study, to the best of our knowledge, has been conducted on the ontogenetic development of the elemental composition of radular teeth in non-patelliform gastropods before.

Overall, we detected here that the presence, distribution, proportions, and ontogeny of elements during radular ontogeny differs between species. This indicates that the general elemental composition of radular teeth as well as the biomineralization processes during ontogeny could be rather unique for each taxon (see also Brooker and Macey 2001; Krings et al. 2022a, 2022b). With regard to the genetics underpinning radular ontogeny, little is known as well. Alkaline phosphatase, ParaHox gene Gsx, and a Lophotrochozoa-specific chitin synthase with a myosin motor domain were found to be expressed during radular ontogeny (Samadi and Steiner 2010; Hohagen and Jackson 2013; Hilgers et al. 2018). As we detected for our species, most elements seem to be constantly secreted from zone 1 to zone 2 (or zone 3 for *L. littorea* and *V. turrita*), and the epithelia secreting the teeth seem to have a similar gene expression within each species. As, however, elemental contents differ between species, genes are potentially expressed differently during radular ontogeny in the taxa; this should be investigated in the future.

Even though some previous studies were conducted for the same or closely related species (for *Cornu aspersum,* see Sollas 1907 and Krings et al. 2019a; for *Littorina littorea,* see Sollas 1907 and Jones et al. 1935; for the neritid *Nerita atramentosa,* see Macey et al. 1997), results cannot be directly compared as the applied techniques strongly differed between studies (see Fig. 1). For example, Sollas (1907) executed protocols including ashing, acid treatments, boiling, staining, or diffusion columns. She found in *C. aspersum* specimens (termed *Helix aspersa* in Sollas 1907), collected during spring, that the radula contains 35% P_2_O_5_ (weight %). In specimens collected during winter, she detected 33% Si (weight %) and an abundance of Ca (no % is given) using her methodology. Employing EDX, Krings et al. (2019a) detected Si and Ca in *C. aspersum*, both in proportions > 1% (atomic %). For *L. littorea* radulae, Sollas (1907) detected 16% P_2_O_5_ (weight %) and the presence of Ca, Fe, and Mg. Jones et al. (1935) specifically tested for Fe and Si by ashing and acid treatment, but could not detect both elements in *L. littorea*. For *N. atramentosa,* Macey et al. (1997) determined by EDX very high proportions (weight %) of Cl, and smaller proportions of Ca, Mg, S, K, Si, and P. All elements detected previously were also detected in this study. As however, we employed a different method, the quantitative results cannot be compared directly. In addition, specifically for Si, the elemental content of radulae could be potentially directly related to the food obtained (i.e., plants with or without Si). This could explain the low proportions of Si in the maturation zone of the here studied *C. aspersum* individuals (Fig. 9) and also the diverging results from Sollas (1907). However, this awaits further investigations, especially since for the plant-consuming gastropods, studied here, no detailed records about the plant species eaten exist.

### The ontogeny of elements in the radula

As stated before, we detected two general trends in the ontogeny of elements from the maturation to the working zone: either the increase of the proportions (pattern A; in *Cornu aspersum*, *Lavigeria nassa*, and *Reymondia horei*) or they decrease (pattern B; in *Anentome helena*, *Littorina littorea*, and *Vittina turrita*). We could not determine an ecological or phylogenetic signal, but such a determination would, however, require a broader taxon sampling.

In previous studies, an increase followed by more or lesser pronounced plateaus in the elemental proportions of the working zone was detected in some polyplacophoran and limpet radulae (Polyplacophora: for *Acanthopleura*, see Lee et al. 2000; and Brooker et al. 2003; for *Acanthopleura*, *Ischnochiton*, *Onithochiton*, and *Plaxiphora*, see Brooker and Macey 2001; Patellogastropoda: for *Patella*, see Runham et al. 1969). A decrease in the elemental content was previously determined as well (for the limpet *Notoacmea*, see Hua and Li 2007). Sometimes the picture seems to be rather puzzling: in the chiton *Clavarizona,* the concentration of Fe, zinc (Zn), and K decrease in the working zone, whereas Ca, P, F, Na, S, and Cl remain constant (Kim et al. 1986). In the chiton *Cryptoplax,* most elemental proportions, e.g., Fe, P, K, and Si, were found to remain more or less constant in the outer tooth rows, whereas Ca, Mg, Na, Al, and S content in the tooth cores decreased (Macey and Brooker 1996). For the patellogastropod *Patelloida*, the Fe content decreased in the tip of the anterior cusps and the posterior region of the posterior cusps, whereas it increased in the anterior region of the anterior cusp (Liddiard et al. 2004). For this species, the content of Si increased in the posterior region of the posterior cusp (Liddiard et al. 2004). In our previous study on the elemental ontogeny of the chiton *Lepidochitona cinerea*, we detected that the Fe content remained constant in the working zone, whereas the proportions of Ca decreased (Krings et al. 2022a).

Decreasing biomineral composition is a sign of chemical wear, which is part of the decay and potentially the loss of proper functionality. This decrease could be explained by a potential elution of elements by either surrounding water or saliva. The saliva has been previously found to be slightly or highly acidic in gastropods (e.g., Moura et al. 2004), especially in carnivorous gastropods (Houbrick and Fretter 1969; Fänge and Lidman 1976; Morton 1990, 2015), as the acidic fluid is used for extraintestinal digestion. This could potentially explain the decrease from the maturation to the working zone in the radula of *Anentome helena*, as this species is also carnivorous feeding on other snails (e.g., Bogan and Hanneman 2013; Strong et al. 2017). In addition, the acid saliva could also be used when foraging on lime-containing items needed for the construction of the shell. Another content of saliva is enzymes, e.g., aminopeptidase (Moura et al. 2004), which could potentially also damage the tooth structure and promote the elution of elements. However, in all species, the pH and the composition of the saliva are unknown. The saliva effect on the elemental composition of radular teeth in ontogeny awaits further investigations in the future.

## Supplementary Information

Below is the link to the electronic supplementary material.

Supplementary file1 (PDF 2685 KB)
