# Supplementary material for: The ontogeny of elements: distinct ontogenetic patterns in the radular tooth mineralization of gastropods
Source: Naturwissenschaften. 2022 Dec 1;109(6):58. doi: 10.1007/s00114-022-01829-2 (PMC9715475; doi:10.1007/s00114-022-01829-2)
Supplement: Supplementary file 1 — Supplementary file1 (PDF 2685 KB) [file 114_2022_1829_MOESM1_ESM.pdf]

The ontogeny of elements: Distinct ontogenetic patterns in the radular tooth mineralization of gastropods

Jan-Ole Brütt[1,2], Stanislav N. Gorb[3], Wencke Krings[1,2,3*]

[1] Department of Behavioral Biology, Institute of Cell and Systems Biology of Animals, Universität Hamburg, Martin-Luther-King-Platz 3, 20146 Hamburg, Germany

[2] Department of Mammalogy and Palaeoanthropology, Leibniz Institute for the Analysis of Biodiversity Change, Martin-Luther-King-Platz 3, 20146 Hamburg, Germany

[3] Department of Functional Morphology and Biomechanics, Zoological Institute, Christian-Albrechts-Universität zu Kiel, Am Botanischen Garten 9, 24118 Kiel, Germany

*corresponding author: wencke.krings@uni-hamburg.de

Supplementary

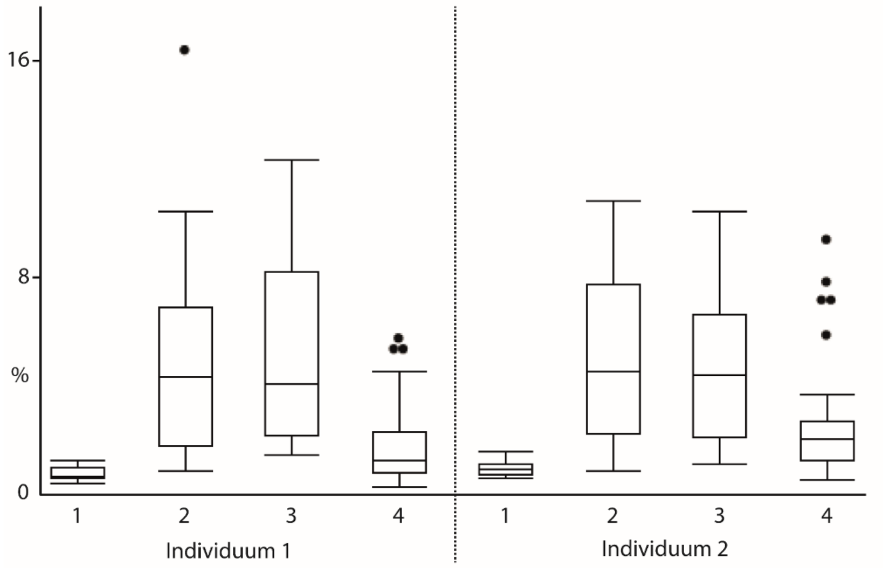

Supplementary Figure 1. *Vittina turrita*, proportions of all elements in atomic percent for each zone (zone 1 = building zone, zone 2 and 3 = maturation zone, zone 4 = working zone) per individuum studied. For Kruskal-Wallis test and pairwise comparison by Wilcoxon method, see Supplementary Table 1.

Supplementary Table 1. Results from Kruskal-Wallis test and pairwise comparison by Wilcoxon method (red p-values = significant, black = not significant) for the proportions of all elements between the ontogenetic zones of the two individual radulae studied in *Vittina turrita*.

Species	Element	Individual 1	Individual 2	1-Way Test. ChiSquare approximation			Wilcoxon method
				ChiSquare	df	p-value	p-value
Vittina turrita	All elements	Zone 1	Zone 1	1.2475	1	0.2640	0.2783
		Zone 2	Zone 2	0.3308	1	0.5652	0.5732
		Zone 3	Zone 3	0.0833	1	0.7728	0.7950
		Zone 4	Zone 4	4.0029	1	0.0454*	0.0462*

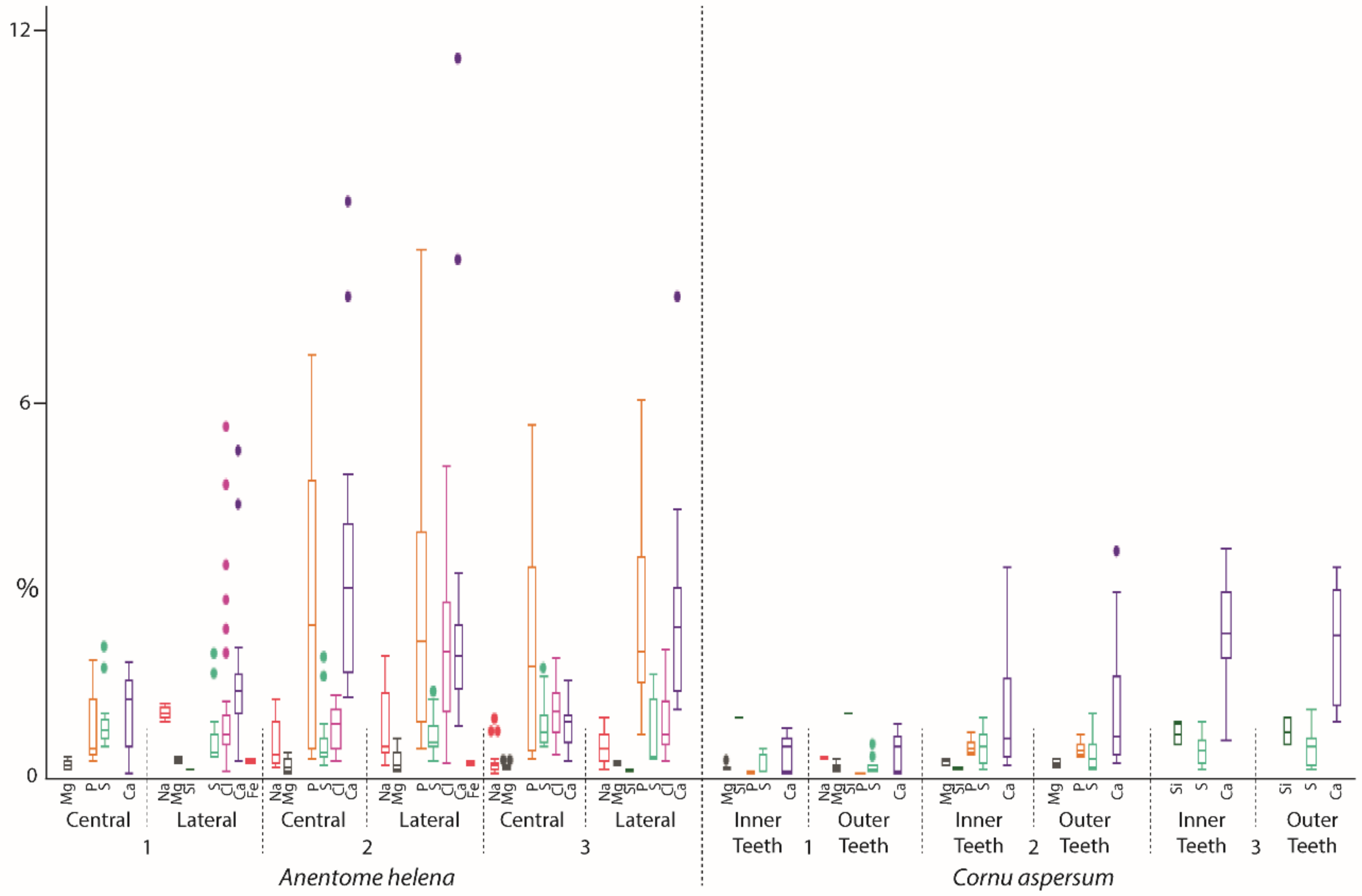

Supplementary Figure 2. *Anentome helena* and *Cornu aspersum*, proportions of all elements in atomic percent for each zone (zone 1 = building zone, zone 2 = maturation zone, zone 3 = working zone) and tooth type. Supplementary Figures 2, 3, and 4 are scaled identically to ease comparison. For values and quantity of measurements, see Supplementary Table 2.

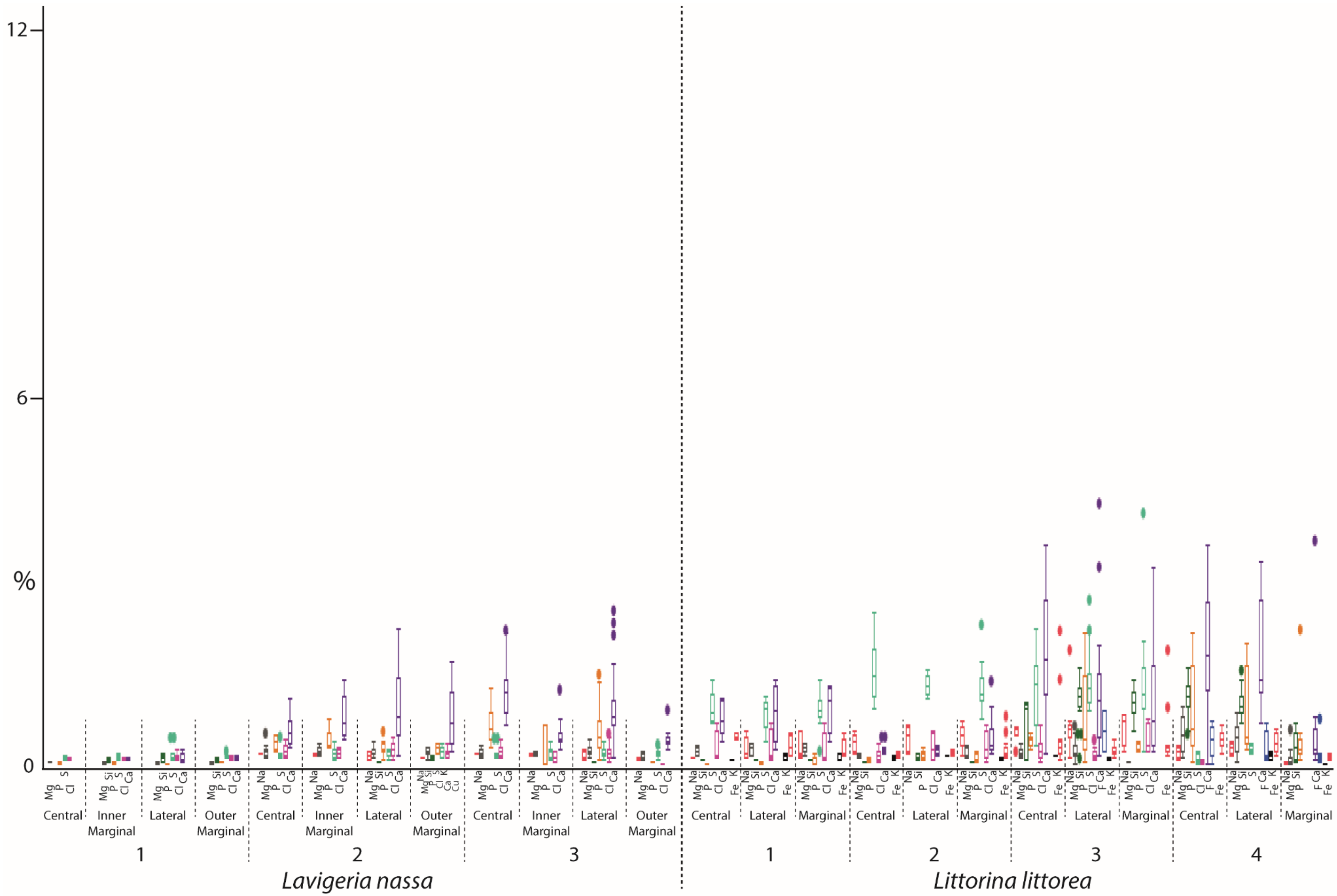

Supplementary Figure 3. *Lavigeria nassa* and *Littorina littorea*, proportions of all elements in atomic percent for each zone and tooth type. Supplementary Figures 2, 3, and 4 are scaled identically to ease comparison. For values and quantity of measurements, see Supplementary Table 2.

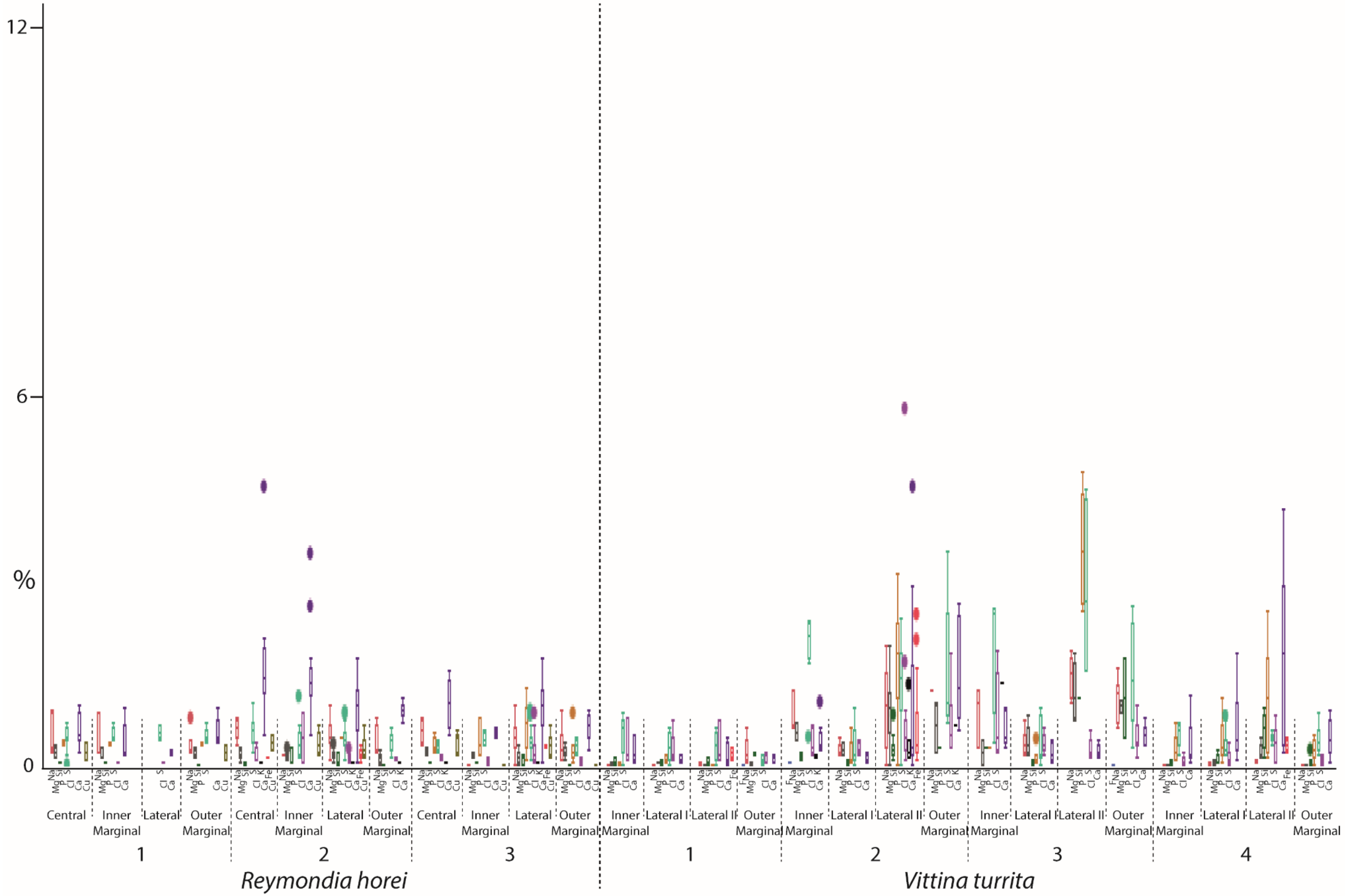

Supplementary Figure 4. *Reymondia horei* and *Vittina turrita*, proportions of all elements in atomic percent for each zone and tooth type. Supplementary Figures 2, 3, and 4 are scaled identically to ease comparison. For values and quantity of measurements, see Supplementary Table 2.

Supplementary Table 2. Means and standart deviations (SD) for the following parameters per species, zone, and tooth type: proportions, atomic %, of F, Na, Mg, Si, P, S, Cl, K, Ca, Fe, Cu, and all elements pooled together. Additionally, the sum of means for all elements is listed. N, quantity of measurements.

Species	Zone	Tooth type	N measurements total	F			Na			Mg			Si			P			S			Cl			K			Ca			Fe			Cu			All elements			
				Mean	Std Dev	N	Mean	Std Dev	N	Mean	Std Dev	N	Mean	Std Dev	N	Mean	Std Dev	N	Mean	Std Dev	N	Mean	Std Dev	N	Mean	Std Dev	N	Mean	Std Dev	N	Mean	Std Dev	N	Mean	Std Dev	N	Mean	Std Dev	N	Sum of means
All	All	All	1027																																					
Anentome helena	1	All	72				1.00	0.11	8	0.18	0.07	24	0.08	0.01	4	0.68	0.58	16	0.71	0.46	36	1.04	1.17	40				1.28	0.79	66	0.22	0.02	4				2.44	1.37	72	7.63
		Central	20							0.16	0.07	16				0.68	0.58	16	0.83	0.39	20							1.02	0.59	20							2.52	1.35	20	5.21
		Lateral	52				1.00	0.11	8	0.22	0.03	8	0.08	0.01	4				0.57	0.50	16	1.04	1.17	40				1.39	0.85	46	0.22	0.02	4				2.41	1.39	52	6.93
	2	All	60				0.71	0.54	31	0.20	0.14	44				2.73	2.09	40	0.58	0.34	60	1.51	1.10	49				2.63	2.02	60	0.19	0.02	4				6.78	3.57	60	15.33
		Central	20				0.47	0.41	8	0.15	0.11	14				2.78	2.18	20	0.51	0.45	20	0.77	0.33	20				3.31	2.08	20							7.67	4.02	20	15.66
		Lateral	40				0.79	0.56	23	0.22	0.15	30				2.68	2.05	20	0.61	0.26	40	2.02	1.17	29				2.28	1.92	40	0.19	0.02	4				6.34	3.29	40	15.13
	3	All	54				0.35	0.27	44	0.14	0.05	18	0.07	0.00	2	2.31	1.58	44	0.72	0.51	18	0.89	0.47	44				1.79	1.39	44							5.68	2.55	44	11.95
		Central	18				0.22	0.25	18	0.12	0.05	14				2.07	1.70	18	0.82	0.45	10	0.98	0.41	18				0.78	0.34	18							4.60	1.85	18	9.59
		Lateral	26				0.43	0.25	26	0.19	0.02	4	0.07	0.00	2	2.47	1.51	26	0.60	0.59	8	0.82	0.50	26				2.48	1.42	26							6.43	2.73	26	13.49
	All	All	176				0.54	0.44	83	0.18	0.11	86	0.08	0.01	6	2.22	1.83	100	0.64	0.41	114	1.16	1.00	133				1.89	1.58	170	0.20	0.02	8				4.73	3.24	176	11.64
Cornu aspersum	1	All	35				0.26	0.01	2	0.12	0.05	16	0.96	0.05	2	0.02	0.01	8	0.14	0.14	16							0.41	0.26	28							0.54	0.37	34	2.45
		Inner Teeth	17							0.12	0.04	7	0.92	.	1	0.03	0.01	2	0.16	0.16	6							0.41	0.26	14							0.50	0.36	17	2.14
		Outer teeth	18				0.26	0.01	2	0.12	0.06	9	0.99	.	1	0.02	0.01	6	0.13	0.13	10							0.42	0.27	14							0.58	0.39	17	2.52
	2	All	63							0.20	0.05	8	0.10	0.01	2	0.43	0.11	26	0.38	0.26	50							1.07	0.95	60							1.55	1.01	62	3.73
		Inner Teeth	31							0.21	0.06	3	0.10	0.01	2	0.44	0.11	13	0.43	0.25	23							1.04	0.93	30							1.54	0.98	31	3.76
		Outer teeth	32							0.19	0.05	5				0.42	0.10	13	0.35	0.27	27							1.10	0.99	30							1.57	1.04	31	3.63
	3	All	40										0.67	0.18	8				0.43	0.27	38							2.18	0.90	40							2.72	1.20	40	6.00
		Inner Teeth	20										0.65	0.17	4				0.40	0.25	18							2.27	0.90	20							2.76	1.16	20	6.08
		Outer teeth	20										0.70	0.22	4				0.46	0.30	20							2.09	0.92	20							2.69	1.26	20	5.94
	All	All	136				0.26	0.01	2	0.15	0.06	24	0.63	0.30	12	0.33	0.20	34	0.36	0.27	104							1.27	1.06	128							1.64	1.25	136	4.64
Lavigeria nassa	1	All	36							0.02	0.01	14	0.08	0.04	16	0.02	0.01	8	0.12	0.08	36	0.09	0.04	30				0.10	0.06	12							0.28	0.18	36	0.71
		Central	7							0.02	0.00	2				0.01	0.01	3	0.10	0.02	7	0.08	0.02	6													0.18	0.02	7	0.39
		Inner Marginal	9							0.02	0.01	3	0.06	0.02	4	0.02	0.02	3	0.10	0.03	9	0.08	0.02	7				0.09	0.03	2							0.23	0.10	9	0.60
		Lateral	12							0.01	0.01	5	0.09	0.06	8	0.01	.	1	0.16	0.13	12	0.11	0.06	10				0.10	0.07	8							0.38	0.25	12	0.86
		Outer Marginal	8							0.02	0.01	4	0.07	0.02	4	0.03	.	1	0.11	0.04	8	0.09	0.02	7				0.10	0.03	2							0.26	0.11	8	0.68
	2	All	57				0.14	0.05	9	0.20	0.08	51	0.06	0.04	4	0.30	0.13	29	0.17	0.07	54	0.21	0.10	35				0.79	0.48	57							1.44	0.53	57	3.31
		Central	12				0.18	0.01	2	0.21	0.10	12				0.35	0.12	6	0.16	0.09	11	0.22	0.11	10				0.54	0.24	12							1.29	0.34	12	2.95
		Inner Marginal	12				0.15	0.01	2	0.21	0.06	11				0.38	0.18	6	0.17	0.09	12	0.17	0.07	9				0.76	0.34	12							1.47	0.44	12	3.31
		Lateral	23				0.14	0.06	4	0.20	0.07	21	0.04	0.01	2	0.25	0.11	13	0.15	0.05	21	0.24	0.12	9				0.96	0.59	23							1.54	0.63	23	3.52
		Outer Marginal	10				0.10	.	1	0.19	0.07	7	0.09	0.05	2	0.25	0.08	4	0.20	0.08	10	0.18	0.09	7				0.73	0.48	10							1.32	0.58	10	3.06
	3	All	104				0.14	0.05	22	0.19	0.06	98	0.03	0.00	2	0.52	0.33	84	0.18	0.08	98	0.18	0.11	83				0.84	0.47	104							1.79	0.88	104	3.87
		Central	20				0.17	0.01	4	0.17	0.06	20				0.64	0.31	20	0.17	0.09	18	0.21	0.10	20				1.20	0.45	20							2.41	0.87	20	4.97
		Inner Marginal	12				0.14	0.01	4	0.17	0.02	10				0.22	0.36	3	0.19	0.09	12	0.12	0.13	2				0.49	0.25	12							0.94	0.55	12	2.27
		Lateral	60				0.14	0.06	10	0.22	0.07	56	0.03	0.00	2	0.51	0.33	60	0.17	0.07	56	0.18	0.11	60				0.88	0.44	60							1.96	0.72	60	4.09
		Outer Marginal	12				0.10	0.01	4	0.15	0.04	12				0.02	.	1	0.18	0.07	12	0.01	.	1				0.41	0.16	12							0.77	0.19	12	1.64
	All	All	197				0.14	0.04	31	0.18	0.08	163	0.07	0.04	22	0.44	0.32	121	0.16	0.08	188	0.17	0.10	148				0.77	0.49	173							1.41	0.90	197	3.34
Littorina littorea	1	All	24				0.24	0.18	12	0.25	0.08	24	0.05	0.00	3	0.03	0.06	8	0.86	0.26	24	0.27	0.23	18	0.12	0.05	6	0.83	0.36	24	0.30	0.16	12				2.45	0.40	24	5.40
		Central	5				0.11	.	1	0.20	0.07	5	0.05	.	1	0.01	.	1	0.91	0.28	5	0.25	0.27	4	0.07	.	1	0.75	0.29	5	0.44	0.07	2				2.29	0.39	5	5.08
		Lateral	6				0.24	0.20	4	0.24	0.09	6	0.05	.	1	0.02	0.01	2	0.82	0.26	6	0.30	0.26	5	0.12	0.07	2	0.83	0.46	6	0.30	0.17	4				2.55	0.34	6	5.47
		Marginal	13				0.26	0.20	7	0.26	0.07	13	0.05	.	1	0.04	0.07	5	0.87	0.27	13	0.25	0.23	9	0.14	0.06	3	0.85	0.35	13	0.25	0.15	6				2.46	0.43	13	5.43
	2	All	33				0.41	0.17	26	0.15	0.08	7	0.06	0.06	4	0.13	0.07	14	1.34	0.39	32	0.22	0.18	22	0.10	0.03	9	0.33	0.24	33	0.20	0.13	30				2.40	0.60	33	5.34
		Central	11				0.36	0.14	10	0.12	0.04	3	0.02	.	1	0.07	0.04	4	1.51	0.48	11	0.14	0.09	10	0.10	0.04	3	0.26	0.09	11	0.16	0.04	9				2.45	0.60	11	5.19
		Lateral	8				0.43	0.19	7				0.10	0.07	2	0.16	0.06	5	1.28	0.16	8	0.26	0.23	4	0.13	0.00	2	0.22	0.06	8	0.17	0.04	8				2.34	0.43	8	5.09
		Marginal	14				0.45	0.19	9	0.17	0.10	4	0.02	.	1	0.13	0.07	5	1.24	0.39	13	0.29	0.22	8	0.08	0.01	4	0.45	0.33	14	0.25	0.19	13				2.41	0.72	14	5.49
	3	All	70	0.53	0.47	2	0.56	0.30	30	0.20	0.13	37	0.99	0.32	53	0.63	0.62	42	1.29	0.56	66	0.21	0.16	32	0.11	0.03	5	1.24	0.94	70	0.31	0.37	58				4.32	1.29	70	10.39
		Central	17				0.49	0.14	7	0.19	0.07	8	0.65	0.45	6	0.37	0.09	7	1.22	0.54	17	0.24	0.17	11	0.14	0.00	2	1.83	1.01	17	0.42	0.55	16				4.29	1.25	17	9.84
		Lateral	39	0.53	0.47	2	0.58	0.35	19	0.21	0.15	28	1.05	0.29	33	0.73	0.69	31	1.29	0.40	38	0.15	0.08	17	0.09	0.01	3	1.06	0.82	39	0.22	0.07	29				4.49	1.19	39	10.40
		Marginal	14				0.61	0.28	4	0.05	.	1	0.98	0.24	14	0.30	0.08	4	1.41	1.00	11	0.39	0.25	4				1.04	0.90	14	0.39	0.48	13				3.86	1.56	14	9.03
	4	All	58	0.28	0.24	44	0.19	0.11	44	0.34	0.29	58	0.83	0.42	56	0.71	0.66	58	0.15	0.10	40	0.03	0.03	2	0.09	0.08	6	1.48	1.07	54	0.34	0.16	46				3.97	2.50	58	8.41
		Central	24	0.37	0.25	18	0.19	0.09	22	0.50	0.28	24	1.09	0.25	24	0.92	0.71	24	0.09	0.07	24	0.03	0.03	2				1.87	1.00	24	0.40	0.13	24				5.31	1.88	24	10.77
		Lateral	16	0.26	0.22	16	0.26	0.08	16	0.40	0.25	16	0.98	0.23	16	0.79	0.65	16	0.23	0.06	16				0.13	0.06	4	1.73	0.82	16	0.34	0.16	16				5.03	1.62	16	10.15
		Marginal	18	0.15	0.21	10	0.02	0.01	6	0.09	0.13	18	0.29	0.24	16	0.36	0.47	18							0.01	0.00	2	0.53	0.91	14	0.10	0.04	6				1.25	1.56	18	2.80
	All	All	185	0.29	0.25	46	0.35	0.25	112	0.27	0.22	126	0.86	0.42	116	0.57	0.62	122	0.96	0.64	162	0.22	0.19	74	0.10	0.05	26	1.09	0.93	181	0.30	0.27	146				3.63	1.82	185	8.64

Reymondia horei	1	All	46				0.45	0.29	18	0.22	0.08	24	0.04	0.00	6	0.37	0.03	12	0.50	0.15	36	0.05	0.00	6				0.47	0.26	36				0.23	0.13	12	1.56	0.71	36	3.89
		Central	12				0.46	0.31	9	0.22	0.08	12	0.04	0.00	3	0.38	0.03	6	0.47	0.21	12							0.60	0.25	12				0.24	0.14	6	1.95	0.56	12	4.36
		Inner Marginal	9				0.65	0.36	3	0.25	0.10	3	0.04	0.00	2	0.37	0.02	3	0.52	0.11	9	0.05	0.00	3				0.39	0.28	9							1.36	0.90	9	3.63
		Lateral	6																0.55	0.10	6	0.05	0.00	3				0.22	0.02	6							0.81	0.09	6	1.63
		Outer Marginal	9				0.34	0.22	6	0.20	0.07	9	0.04	.	1	0.36	0.04	3	0.49	0.10	9							0.55	0.20	9				0.23	0.13	6	1.75	0.47	9	3.96
	2	All	71				0.47	0.24	21	0.16	0.08	62	0.09	0.10	13	0.45		1	0.45	0.21	69	0.16	0.18	24	0.06	0.01	5	1.18	0.67	70	0.18	0.09	9	0.34	0.16	24	2.11	0.82	71	5.65
		Central	12				0.61	0.16	5	0.20	0.08	10	0.04	0.01	4				0.58	0.24	12	0.17	0.13	8	0.07	.	1	1.65	1.02	12	0.14	.	1	0.40	0.18	2	2.85	1.15	12	6.71
		Inner Marginal	16				0.19	0.01	2	0.15	0.06	12	0.14	0.15	3				0.40	0.26	14	0.47	0.56	2				1.45	0.77	15				0.38	0.19	6	2.07	0.85	16	5.25
		Lateral	27				0.49	0.24	10	0.18	0.08	24	0.11	0.10	5	0.45	.	1	0.45	0.18	27	0.11	0.07	12	0.06	0.01	3	0.98	0.43	27	0.19	0.09	8	0.31	0.16	16	2.11	0.59	27	5.44
		Outer Marginal	16				0.39	0.27	4	0.13	0.07	16	0.02	.	1				0.41	0.15	16	0.13	0.04	2	0.05	.	1	0.92	0.12	16							1.58	0.32	16	3.63
	3	All	72				0.42	0.26	44	0.17	0.09	58	0.06	0.06	14	0.52	0.33	55	0.38	0.15	70	0.19	0.16	67	0.06	0.01	10	0.87	0.38	70	0.32	0.02	2	0.33	0.17	52	2.46	0.96	72	5.78
		Central	8				0.58	0.17	6	0.23	0.05	6	0.04	0.00	2	0.34	0.11	8	0.34	0.06	8	0.11	0.04	8	0.07	0.00	2	1.02	0.39	8				0.39	0.16	8	2.83	0.71	8	5.95
		Inner Marginal	8				0.02	0.01	3	0.18	0.05	4	0.05	0.00	2	0.44	0.30	3	0.43	0.10	6	0.06	0.04	5				0.54	0.05	6				0.02	0.01	2	1.04	0.46	8	2.78
		Lateral	44				0.47	0.23	25	0.15	0.09	38	0.07	0.08	8	0.65	0.33	34	0.40	0.17	44	0.24	0.17	44	0.05	0.01	8	0.96	0.39	44	0.32	0.02	2	0.35	0.15	40	2.84	0.81	44	6.50
		Outer Marginal	12				0.34	0.30	10	0.23	0.09	10	0.03	0.00	2	0.28	0.23	10	0.32	0.09	12	0.09	0.03	10				0.64	0.22	12				0.03	0.01	2	1.75	0.52	12	3.71
	All	All	179				0.44	0.26	83	0.18	0.08	144	0.07	0.07	33	0.50	0.30	68	0.43	0.18	175	0.17	0.16	97	0.06	0.01	15	0.91	0.56	176	0.21	0.10	11	0.32	0.17	88	2.14	0.92	179	5.43
Vittina turrita	1	All	24	0.02	0.01	2	0.15	0.19	13	0.03	0.02	9	0.10	0.07	8	0.06	0.05	7	0.30	0.26	22	0.25	0.25	18				0.16	0.15	20	0.21	0.09	4				0.84	0.28	22	2.12
		Inner Marginal	6				0.03	.	1	0.04	0.01	2	0.08	0.06	2	0.04	0.01	2	0.52	0.31	5	0.35	0.39	3				0.27	0.21	5							1.07	0.34	5	2.40
		Lateral I	5				0.02	0.01	3	0.05	.	1	0.07	0.06	2	0.11	0.05	3	0.32	0.24	5	0.28	0.27	5				0.11	0.06	4							0.80	0.21	5	1.76
		Lateral II	6				0.04	0.01	2	0.02	0.01	2	0.07	0.07	2	0.03	0.01	2	0.33	0.23	5	0.34	0.37	3				0.17	0.20	4	0.21	0.09	4				0.89	0.17	5	2.10
		Outer Marginal	7	0.02	0.01	2	0.25	0.21	7	0.04	0.03	4	0.20	0.01	2				0.09	0.07	7	0.14	0.07	7				0.09	0.05	7							0.66	0.23	7	1.49
	2	All	46	0.05	.	1	0.77	0.59	19	0.77	0.50	22	0.24	0.16	25	1.47	0.95	29	1.35	0.83	44	0.62	0.92	40	0.36	0.38	10	0.80	0.99	44	0.66	0.64	23				4.97	3.41	44	12.06
		Inner Marginal	8	0.05	.	1	0.84	0.33	3	0.56	0.20	2	0.18	0.04	8				1.92	0.63	8	0.39	0.19	8	0.16	0.04	2	0.44	0.27	8							3.44	1.28	8	7.98
		Lateral I	6				0.27	0.11	5	0.27	0.15	2	0.07	0.06	2	0.21	0.24	5	0.29	0.37	5	0.30	0.08	5				0.12	0.07	5							1.33	0.45	5	2.86
		Lateral II	24				0.95	0.67	10	0.91	0.53	14	0.30	0.19	13	1.74	0.82	24	1.33	0.69	24	0.77	1.21	21	0.38	0.43	7	0.88	1.17	24	0.66	0.64	23				6.45	3.56	24	14.37
		Outer Marginal	8				1.25	.	1	0.63	0.43	4	0.30	0.02	2				1.51	1.06	7	0.68	0.58	6	0.68	.	1	1.43	0.81	7							4.25	3.01	7	10.73
	3	All	28	0.03	.	1	0.92	0.51	22	0.74	0.48	15	0.43	0.61	9	1.46	1.79	13	1.55	1.26	24	0.46	0.37	23	1.35		1	0.39	0.24	24	0.79	0.27	5				4.70	2.98	24	12.03
		Inner Marginal	7				0.85	0.50	3	0.22	0.28	2	0.31	.	1	0.29	.	1	1.87	0.98	5	0.75	0.76	4	1.35	.	1	0.61	0.29	5							4.07	1.07	5	10.32
		Lateral I	7				0.40	0.19	7	0.40	0.26	5	0.04	0.04	5	0.15	0.13	7	0.36	0.37	7	0.37	0.14	7				0.22	0.13	7							1.81	0.46	7	3.75
		Lateral II	6				1.45	0.35	5	1.20	0.48	4	1.09	.	1	3.53	0.93	5	2.91	1.40	5	0.28	0.17	5				0.25	0.11	5	0.79	0.27	5				9.59	1.48	5	20.30
		Outer Marginal	8	0.03	.	1	1.10	0.36	7	0.98	0.09	4	1.12	0.92	2				1.54	0.86	7	0.50	0.27	7				0.52	0.16	7							4.54	1.25	7	10.33
	4	All	64				0.03	0.02	20	0.16	0.13	27	0.21	0.26	64	0.55	0.56	54	0.42	0.18	64	0.17	0.18	64				0.82	0.90	64	0.33	0.09	16				2.25	1.96	64	4.94
		Inner Marginal	12				0.01	0.00	3	0.01	0.00	2	0.04	0.02	12	0.28	0.22	7	0.51	0.19	12	0.09	0.07	12				0.39	0.37	12							1.20	0.64	12	2.53
		Lateral I	20				0.02	0.01	6	0.04	0.03	5	0.14	0.06	20	0.43	0.30	18	0.33	0.18	20	0.13	0.10	20				0.66	0.50	20							1.66	0.93	20	3.41
		Lateral II	16				0.06	0.01	6	0.25	0.10	16	0.58	0.26	16	1.04	0.78	16	0.46	0.07	16	0.35	0.26	16				1.73	1.29	16	0.33	0.09	16				4.75	2.35	16	9.55
		Outer Marginal	16				0.02	0.01	5	0.02	0.01	4	0.06	0.06	16	0.27	0.16	13	0.44	0.20	16	0.09	0.06	16				0.45	0.28	16							1.27	0.49	16	2.62
	All	All	154	0.03	0.02	4	0.50	0.57	74	0.45	0.48	73	0.23	0.28	106	0.89	1.02	103	0.84	0.85	154	0.35	0.55	145	0.45	0.47	11	0.66	0.83	152	0.50	0.50	43				3.21	2.94	154	8.11

Supplementary Table 3. Results from Kruskal-Wallis test and pairwise comparison by Wilcoxon method (orange and red p-values = significant or highly significant) for the proportions of F, Na, Mg, Si, P, S, Cl, K, Ca, Fe, Cu, and all elements pooled together between the ontogenetic zones for each species.

Species	Element	Zone	Zone	1-Way Test. ChiSquare approximation			Wilcoxon method
				ChiSquare	df	p-value	p-value
Anentome helena	Na	2	1	22.2659	2	<.0001*	0.1303
		3	2				0.0015*
		3	1				<.0001*
	Mg	2	1	1.9005	2	0.3867	0.9386
		3	2				0.4755
		3	1				0.0502
	Si	3	1	2.7022	1	0.1002	0.1588
	P	3	1	17.9541	2	0.0001*	<.0001*
		2	1				0.0001*
		3	2				0.5787
	S	3	2	2.1104	2	0.3481	0.5611
		3	1				0.4912
		2	1				0.1626
	Cl	2	1	11.2873	2	0.0035*	0.0036*
		3	1				0.3311
		3	2				0.0071*
	Ca	2	1	34.8816	2	<.0001*	<.0001*
	Ca	3	1				0.1916
	Ca	3	2				0.0020*
	Fe	2	1	2.0833	1	0.1489	0.1939
	All elements	2	1	88.7166	2	<.0001*	<.0001*
		3	1				<.0001*
		3	2				0.1396
Cornu aspersum	Mg	2	1	9.7707	1	0.0018*	0.0020*
	Si	3	2	6.9712	2	0.0306*	0.0502
		2	1				0.2453
		3	1				0.0896
	P	2	1	17.8504	1	<.0001*	<.0001*
	S	2	1	22.7690	2	<.0001*	<.0001*
		3	1				<.0001*
		3	2				0.5782
	Ca	3	1	50.6911	2	<.0001*	<.0001*
		3	2				<.0001*
		2	1				0.0051*
	All elements	3	1	63.4166	2	<.0001*	<.0001*
		2	1				<.0001*
		3	2				<.0001*
Lavigeria nassa	Na	3	2	0.2515	1	0.6160	0.6314
	Mg	3	1	38.4571	2	<.0001*	<.0001*
		2	1				<.0001*
		3	2				0.5833
	Si	3	2	3.4069	2	0.1821	0.3476
		2	1				0.4212
		3	1				0.1058
	P	3	1	33.0450	2	<.0001*	<.0001*
		3	2				0.0002*
		2	1				<.0001*
	S	3	1	24.7520	2	<.0001*	<.0001*
		2	1				<.0001*
		3	2				0.4331
	Cl	3	1	36.9750	2	<.0001*	<.0001*
		2	1				<.0001*
		3	2				0.2060
	Ca	3	1	33.5654	2	<.0001*	<.0001*
		2	1				<.0001*
		3	2				0.2987
	All elements	3	1	89.5694	2	<.0001*	<.0001*
		2	1				<.0001*

		3	2				0.0113*
Littorina littorea	F	4	3	2.2756	1	0.1314	0.1385
	Na	3	1	49.1450	3	<.0001*	0.0001*
		3	2				0.0087*
		2	1				0.0056*
		4	1				0.9125
		4	2				<.0001*
		4	3				<.0001*
	Mg	4	2	6.6881	3	0.0825	0.2077
		4	3				0.1047
		3	2				0.2471
		4	1				0.6722
		3	1				0.0303*
		2	1				0.0089*
	Si	3	1	21.2243	3	<.0001*	0.0040*
		3	2				0.0014*
		4	1				0.0075*
		4	2				0.0026*
		2	1				0.6933
		4	3				0.0494*
	P	4	1	44.2263	3	<.0001*	<.0001*
		4	2				<.0001*
		3	2				<.0001*
		3	1				<.0001*
		2	1				0.0031*
		4	3				0.6298
	S	3	1	102.9703	3	<.0001*	<.0001*
		2	1				<.0001*
		3	2				0.4578
		4	1				<.0001*
		4	2				<.0001*
		4	3				<.0001*
	Cl	3	2	5.3959	3	0.1450	0.7048
		3	1				0.7233
		2	1				0.7232
		4	1				0.0261*
		4	2				0.0530
		4	3				0.0253*
	K	3	2	1.2410	3	0.7432	0.3480
		3	1				1.0000
		4	1				0.6781
		4	2				0.5949
		4	3				0.5209
		2	1				0.5127
	Ca	3	2	40.7542	3	<.0001*	<.0001*
		4	2				<.0001*
		4	1				0.0162*
		3	1				0.0843
		4	3				0.2945
		2	1				<.0001*
	Fe	4	2	19.0414	3	<.0001*	0.0002*
		4	3				0.0044*
		3	2				0.0038*
		4	1				0.3178
		3	1				0.6122
		2	1				0.1143
	All elements	3	2	53.6241	3	<.0001*	<.0001*
		3	1				<.0001*
		4	2				0.0025*
		4	1				0.0036*
		2	1				0.4330
		4	3				0.3902
Reymondia horei	Na	2	1	0.4498	2	0.7986	0.6521
		3	1				0.9629

		3	2				0.5096
	Mg	3	2	5.6003	2	0.0608	0.6250
		3	1				0.0540
		2	1				0.0199*
	Si	2	1	1.4081	2	0.4946	0.5362
		3	1				1.0000
		3	2				0.2417
	P	2	1	0.4147	2	0.8127	0.1394
		3	1				0.6587
		3	2				0.9506
	S	2	1	20.0553	2	<.0001*	0.0250*
		3	2				0.0358*
		3	1				<.0001*
	Cl	3	1	14.6722	2	0.0007*	0.0002*
		2	1				0.0020*
		3	2				0.2203
	K	3	2	0.3168	1	0.5736	0.6169
	Ca	2	1	52.5669	2	<.0001*	<.0001*
		3	1				<.0001*
		3	2				0.0018*
	Fe	3	2	3.1537	1	0.0758	0.0974
	All elements	3	1	25.3443	2	<.0001*	<.0001*
		2	1				0.0010*
		3	2				0.0056*
	Cu	3	1	5.2295	2	0.0732	0.0336*
		2	1				0.0330*
		3	2				0.9643
Vittina turrita	F	2	1	2.2500	2	0.3247	0.5403
		3	1				1.0000
		3	2				1.0000
	Na	3	1	47.6544	3	<.0001*	<.0001*
		2	1				0.0005*
		3	2				0.3015
		4	1				0.0171*
		4	2				<.0001*
		4	3				<.0001*
	Mg	2	1	38.0484	3	<.0001*	<.0001*
		3	1				0.0002*
		4	1				0.0432*
		3	2				0.9753
		4	3				<.0001*
		4	2				<.0001*
	Si	2	1	8.3696	3	0.0390*	0.0080*
		4	1				0.4093
		3	1				0.7342
		4	3				0.9933
		3	2				0.5839
		4	2				0.0086*
	P	4	1	23.8280	3	<.0001*	0.0001*
		2	1				0.0006*
		3	1				0.0047*
		4	3				0.8490
		3	2				0.6632
		4	2				0.0003*
	S	2	1	44.6131	3	<.0001*	<.0001*
		4	1				0.0114*
		3	1				0.0001*
		3	2				0.6532
		4	3				0.0006*
		4	2				<.0001*
	Cl	2	1	54.5165	3	<.0001*	0.0001*
		3	1				0.0019*
		3	2				0.4883
		4	1				0.0552

		4	3				<.0001*
		4	2				<.0001*
	K	3	2	2.6190	1	0.1056	0.1453
	Ca	4	1	27.2877	3	<.0001*	<.0001*
		2	1				<.0001*
		3	1				0.0002*
		4	3				0.0898
		4	2				0.4876
		3	2				0.5293
	Fe	2	1	5.0775	2	0.0790	0.0514
		4	1				0.0654
		4	2				0.3106
	All elements	2	1	62.5687	3	<.0001*	<.0001*
		4	1				<.0001*
		3	1				<.0001*
		3	2				0.9591
		4	3				<.0001*
		4	2				<.0001*

Supplementary Table 4. Results from Kruskal-Wallis test and pairwise comparison by Wilcoxon method (orange and red p-values = significant or highly significant) for the proportions of F, Na, Mg, Si, P, S, Cl, K, Ca, Fe, Cu, and all elements pooled together between the tooth types of each ontogenetic zone for each species.

Species	Element	Tooth and Zone	Tooth and Zone	1-Way Test. ChiSquare approximation			Wilcoxon method
				ChiSquare	df	p-value	p-value
Anentome helena	**Na**	Lateral 2	Central 3	33.3314	4	<.0001*	<.0001*
		Lateral 3	Central 3				0.0006*
		Lateral 1	Central 3				<.0001*
		Lateral 2	Central 2				0.0286*
		Lateral 1	Central 2				0.0406*
		Lateral 3	Central 2				0.7918
		Lateral 2	Lateral 1				0.2887
		Central 3	Central 2				0.0371*
		Lateral 3	Lateral 2				0.0363*
		Lateral 3	Lateral 1				<.0001*
	**Mg**	Lateral 1	Central 2	9.5843	5	0.0879	0.0015*
		Lateral 2	Central 1				0.0639
		Lateral 1	Central 3				0.0297*
		Lateral 3	Central 3				0.0380*
		Lateral 2	Central 2				0.1544
		Lateral 1	Central 1				0.1420
		Lateral 3	Central 1				0.2021
		Lateral 2	Central 2				0.3269
		Lateral 3	Lateral 2				0.5592
		Lateral 3	Central 2				0.9148
		Central 3	Lateral 1				0.9450
		Lateral 2	Central 1				0.8020
		Central 2	Lateral 1				0.4176
		Lateral 3	Central 1				0.0735
		Central 3	Central 2				0.1696
	**Si**	Lateral 3	Lateral 1	2.7022	1	0.1002	0.1588
	**P**	Lateral 3	Central 1	19.7791	4	0.0006*	<.0001*
		Lateral 2	Central 1				<.0001*
		Central 2	Central 1				0.0068*
		Central 3	Central 1				0.0272*
		Lateral 3	Central 3				0.2722
		Lateral 2	Central 3				0.2859
		Lateral 3	Central 2				0.8942
		Lateral 3	Lateral 2				0.9558
		Lateral 2	Central 2				0.9676
		Central 3	Central 2				0.3129
	**S**	Lateral 2	Central 2	27.1975	5	<.0001*	0.0040*
		Lateral 2	Lateral 1				0.0103*
		Central 3	Central 2				0.0064*
		Lateral 1	Central 2				0.4737
		Lateral 3	Central 2				0.7796
		Central 3	Central 1				0.4412
		Lateral 3	Lateral 1				0.1668
		Lateral 3	Central 3				0.0454*
		Lateral 1	Central 3				0.0142*
		Lateral 3	Central 1				0.0269*
		Lateral 2	Central 3				0.1294
		Lateral 3	Lateral 2				0.0544
		Lateral 1	Central 1				0.0015*
		Lateral 2	Central 1				0.0078*
		Central 2	Central 1				0.0003*
	**Cl**	Lateral 2	Lateral 1	25.7539	4	<.0001*	0.0001*
		Lateral 2	Central 2				0.0001*
		Lateral 2	Central 3				0.0015*
		Central 3	Central 2				0.1601
		Lateral 3	Lateral 1				0.9634
		Lateral 3	Central 2				0.7733
		Lateral 1	Central 2				0.5356
		Lateral 3	Central 3				0.1520
		Lateral 1	Central 3				0.0586
		Lateral 3	Lateral 2				<.0001*
	**Ca**	Lateral 2	Central 3	72.3733	5	<.0001*	<.0001*
		Lateral 2	Lateral 1				<.0001*
		Lateral 3	Central 3				<.0001*
		Lateral 3	Lateral 1				<.0001*
		Lateral 2	Central 1				<.0001*
		Lateral 1	Central 3				0.0002*
		Central 2	Central 1				<.0001*
		Lateral 3	Central 1				<.0001*
		Lateral 1	Central 1				0.1968
		Lateral 3	Lateral 2				0.1829
		Central 3	Central 1				0.1397
		Lateral 3	Central 2				0.0879
		Lateral 2	Central 2				0.0099*
		Central 3	Central 2				<.0001*
		Lateral 1	Central 2				<.0001*
	**Fe**	Lateral 2	Lateral 1	2.0833	1	0.1489	0.1939
	**All elements**	Lateral 2	Lateral 1	92.1825	5	<.0001*	<.0001*
		Lateral 3	Lateral 1				<.0001*
		Lateral 2	Central 1				<.0001*
		Lateral 3	Central 1				<.0001*
		Central 2	Central 1				<.0001*
		Central 3	Central 1				0.0003*
		Lateral 2	Central 3				0.0259*
		Lateral 3	Central 3				0.0144*
		Lateral 3	Lateral 2				0.6507

		Lateral 1	Central 1				0.8259
		Lateral 3	Central 2				0.3936
		Lateral 2	Central 2				0.2303
		Central 3	Central 2				0.0105*
		Lateral 1	Central 3				<.0001*
		Lateral 1	Central 2				<.0001*
Cornu aspersum	**Mg**	Outer teeth 2	Outer teeth 1	9.8827	3	0.0196*	0.0329*
		Outer teeth 2	Inner teeth 1				0.0230*
		Inner teeth 2	Inner teeth 1				0.0402*
		Outer teeth 1	Inner teeth 1				0.9156
		Outer teeth 2	Inner teeth 2				0.7628
		Outer teeth 1	Inner teeth 2				0.0645
	**Si**	Inner teeth 3	Inner teeth 2	7.5962	4	0.1075	0.1052
		Outer teeth 3	Inner teeth 2				0.1052
		Outer teeth 1	Inner teeth 3				0.2888
		Outer teeth 3	Inner teeth 3				0.4705
		Outer teeth 1	Inner teeth 2				0.5403
		Outer teeth 1	Inner teeth 1				1.0000
		Outer teeth 3	Inner teeth 1				0.7237
		Inner teeth 2	Inner teeth 1				0.5403
		Inner teeth 3	Inner teeth 1				0.2888
		Outer teeth 3	Outer teeth 1				0.2888
	**P**	Outer teeth 2	Outer teeth 1	18.0703	3	0.0004*	0.0007*
		Inner teeth 2	Inner teeth 1				0.0338*
		Outer teeth 2	Inner teeth 1				0.0338*
		Outer teeth 2	Inner teeth 2				0.6816
		Outer teeth 1	Inner teeth 1				0.2200
		Outer teeth 1	Inner teeth 2				0.0007*
	**S**	Outer teeth 2	Outer teeth 1	24.5911	5	0.0002*	0.0005*
		Outer teeth 3	Outer teeth 1				0.0003*
		Inner teeth 2	Inner teeth 1				0.0071*
		Outer teeth 2	Inner teeth 1				0.0208*
		Outer teeth 3	Inner teeth 1				0.0068*
		Inner teeth 3	Inner teeth 1				0.0164*
		Outer teeth 3	Outer teeth 2				0.2080
		Outer teeth 3	Inner teeth 3				0.3494
		Outer teeth 1	Inner teeth 1				0.4125
		Outer teeth 3	Inner teeth 2				0.8170
		Outer teeth 2	Inner teeth 3				0.6514
		Inner teeth 3	Inner teeth 2				0.5632
		Outer teeth 2	Inner teeth 2				0.1954
		Outer teeth 1	Inner teeth 3				0.0010*
		Outer teeth 1	Inner teeth 2				0.0003*
	**Ca**	Outer teeth 3	Inner teeth 1	50.7547	5	<.0001*	<.0001*
		Outer teeth 3	Outer teeth 1				<.0001*
		Inner teeth 3	Inner teeth 1				<.0001*
		Inner teeth 3	Inner teeth 2				0.0002*
		Outer teeth 3	Inner teeth 2				0.0003*
		Outer teeth 3	Outer teeth 2				0.0006*
		Outer teeth 2	Inner teeth 1				0.0425*
		Outer teeth 2	Outer teeth 1				0.0479*
		Inner teeth 2	Inner teeth 1				0.0539
		Outer teeth 2	Inner teeth 2				0.8130
		Outer teeth 1	Inner teeth 1				0.8359
		Outer teeth 3	Inner teeth 3				0.6949
		Outer teeth 1	Inner teeth 2				0.0571
		Outer teeth 2	Inner teeth 3				0.0003*
		Outer teeth 1	Inner teeth 3				<.0001*
	**All elements**	Outer teeth 3	Inner teeth 1	63.6305	5	<.0001*	<.0001*
		Outer teeth 3	Outer teeth 1				<.0001*
		Outer teeth 2	Inner teeth 1				<.0001*
		Inner teeth 3	Inner teeth 1				<.0001*
		Inner teeth 2	Inner teeth 1				<.0001*
		Outer teeth 2	Outer teeth 1				0.0003*
		Inner teeth 3	Inner teeth 2				0.0008*
		Outer teeth 3	Inner teeth 2				0.0013*
		Outer teeth 3	Outer teeth 2				0.0018*
		Outer teeth 1	Inner teeth 1				0.4587
		Outer teeth 2	Inner teeth 2				0.9215
		Outer teeth 3	Inner teeth 3				0.9892
		Outer teeth 2	Inner teeth 3				0.0013*
		Outer teeth 1	Inner teeth 2				0.0005*
		Outer teeth 1	Inner teeth 3				<.0001*
Lavigeria nassa	**Na**	Lateral 3	Lateral 2	8.0175	7	0.3311	0.7754
		Lateral 2	Central 2				1.0000
		Lateral 2	Central 3				1.0000
		Lateral 2	Inner Marginal 2				1.0000
		Lateral 2	Inner Marginal 3				1.0000
		Outer Marginal 2	Lateral 2				1.0000
		Outer Marginal 3	Outer Marginal 2				0.7237
		Outer Marginal 2	Central 2				0.5403
		Outer Marginal 2	Inner Marginal 2				0.5403
		Lateral 3	Central 2				0.7464
		Lateral 3	Inner Marginal 2				0.7464
		Central 3	Central 2				0.4745
		Inner Marginal 3	Inner Marginal 2				0.4745
		Lateral 3	Central 3				0.6198
		Lateral 3	Inner Marginal 3				0.6198
		Outer Marginal 3	Lateral 2				0.4705
		Inner Marginal 2	Central 2				0.2453
		Outer Marginal 2	Lateral 3				0.6314
		Outer Marginal 2	Central 3				0.2888
		Outer Marginal 2	Inner Marginal 3				0.2888
		Inner Marginal 2	Central 3				0.1052

		Inner Marginal 3	Central 2				0.1052
		Outer Marginal 3	Central 2				0.1052
		Outer Marginal 3	Inner Marginal 2				0.1052
		Outer Marginal 3	Lateral 3				0.2268
		Inner Marginal 3	Central 3				0.0304*
		Outer Marginal 3	Central 3				0.0304*
		Outer Marginal 3	Inner Marginal 3				0.0304*
	**Mg**	Lateral 3	Lateral 1	51.0047	11	<.0001*	0.0002*
		Lateral 3	Inner Marginal 1				0.0039*
		Lateral 3	Central 1				0.0180*
		Lateral 3	Central 3				0.0220*
		Lateral 2	Lateral 1				0.0007*
		Lateral 3	Inner Marginal 3				0.0680
		Lateral 2	Inner Marginal 1				0.0068*
		Lateral 2	Central 1				0.0251*
		Central 3	Central 1				0.0257*
		Outer Marginal 3	Lateral 1				0.0018*
		Outer Marginal 3	Outer Marginal 1				0.0043*
		Outer Marginal 3	Inner Marginal 1				0.0115*
		Inner Marginal 2	Inner Marginal 1				0.0121*
		Central 2	Central 1				0.0350*
		Outer Marginal 3	Central 1				0.0356*
		Inner Marginal 3	Inner Marginal 1				0.0140*
		Inner Marginal 2	Central 1				0.0364*
		Outer Marginal 2	Lateral 1				0.0049*
		Lateral 3	Lateral 2				0.3166
		Inner Marginal 3	Central 1				0.0402*
		Outer Marginal 2	Outer Marginal 1				0.0104*
		Outer Marginal 2	Inner Marginal 1				0.0222*
		Lateral 2	Central 3				0.2101
		Lateral 3	Central 2				0.4590
		Outer Marginal 2	Central 1				0.0550
		Inner Marginal 2	Central 3				0.2305
		Lateral 2	Inner Marginal 3				0.5120
		Outer Marginal 2	Central 3				0.5240
		Lateral 3	Inner Marginal 2				0.7540
		Outer Marginal 2	Inner Marginal 3				0.5565
		Outer Marginal 1	Lateral 1				0.5582
		Inner Marginal 3	Central 3				0.8775
		Lateral 2	Central 2				0.8956
		Inner Marginal 2	Central 2				0.8773
		Inner Marginal 1	Central 1				1.0000
		Outer Marginal 2	Lateral 2				1.0000
		Outer Marginal 2	Central 2				0.9662
		Outer Marginal 2	Inner Marginal 2				0.9273
		Outer Marginal 1	Inner Marginal 1				0.8516
		Outer Marginal 1	Central 1				0.8026
		Lateral 1	Inner Marginal 1				0.3894
		Lateral 1	Central 1				0.2801
		Lateral 2	Inner Marginal 2				0.6049
		Inner Marginal 3	Central 2				0.4877
		Outer Marginal 3	Outer Marginal 2				0.1626
		Central 3	Central 2				0.2576
		Inner Marginal 3	Inner Marginal 2				0.1452
		Outer Marginal 3	Central 3				0.2348
		Outer Marginal 3	Inner Marginal 3				0.1050
		Outer Marginal 2	Lateral 3				0.5044
		Outer Marginal 3	Central 2				0.0643
		Outer Marginal 1	Inner Marginal 3				0.0057*
		Outer Marginal 3	Inner Marginal 2				0.0135*
		Inner Marginal 1	Central 2				0.0113*
		Outer Marginal 1	Inner Marginal 2				0.0047*
		Lateral 1	Inner Marginal 3				0.0024*
		Outer Marginal 3	Lateral 2				0.0312*
		Outer Marginal 1	Central 2				0.0043*
		Lateral 1	Inner Marginal 2				0.0020*
		Lateral 1	Central 2				0.0017*
		Inner Marginal 1	Central 3				0.0070*
		Outer Marginal 1	Central 3				0.0022*
		Outer Marginal 1	Lateral 2				0.0021*
		Lateral 1	Central 3				0.0007*
		Outer Marginal 3	Lateral 3				0.0013*
		Outer Marginal 1	Lateral 3				0.0009*
	**Si**	Outer Marginal 1	Lateral 2	6.1091	5	0.2957	0.1052
		Outer Marginal 1	Lateral 3				0.1052
		Lateral 1	Inner Marginal 1				0.3949
		Outer Marginal 2	Lateral 2				0.2453
		Outer Marginal 2	Lateral 3				0.2453
		Outer Marginal 1	Inner Marginal 1				0.6650
		Outer Marginal 2	Inner Marginal 1				0.6386
		Outer Marginal 2	Outer Marginal 1				0.8170
		Lateral 3	Lateral 2				1.0000
		Outer Marginal 2	Lateral 1				1.0000
		Outer Marginal 1	Lateral 1				0.4962
		Lateral 2	Lateral 1				0.3608
		Lateral 3	Lateral 1				0.3608
		Lateral 2	Inner Marginal 1				0.1588
		Lateral 3	Inner Marginal 1				0.1052
	**P**	Lateral 3	Central 1	45.6191	11	<.0001*	0.0039*
		Lateral 3	Inner Marginal 1				0.0039*
		Lateral 3	Lateral 1				0.0937
		Lateral 3	Lateral 2				0.0014*
		Lateral 3	Inner Marginal 3				0.1332
		Central 3	Central 1				0.0071*

		Lateral 3	Central 2				0.2694
		Lateral 2	Central 1				0.0102*
		Lateral 2	Inner Marginal 1				0.0102*
		Central 3	Central 2				0.0331*
		Lateral 3	Inner Marginal 2				0.3964
		Lateral 2	Lateral 1				0.1340
		Central 2	Central 1				0.0269*
		Inner Marginal 2	Central 1				0.0275*
		Inner Marginal 2	Inner Marginal 1				0.0275*
		Outer Marginal 2	Central 1				0.0497*
		Outer Marginal 2	Inner Marginal 1				0.0497*
		Lateral 2	Inner Marginal 3				0.4171
		Outer Marginal 2	Lateral 1				0.2888
		Outer Marginal 2	Outer Marginal 1				0.2888
		Outer Marginal 1	Central 1				0.3458
		Outer Marginal 2	Inner Marginal 3				0.5926
		Outer Marginal 3	Central 1				0.6171
		Outer Marginal 2	Lateral 2				0.9093
		Inner Marginal 1	Central 1				1.0000
		Inner Marginal 2	Central 2				1.0000
		Inner Marginal 3	Central 1				1.0000
		Inner Marginal 3	Inner Marginal 1				1.0000
		Lateral 1	Central 1				1.0000
		Lateral 1	Inner Marginal 1				1.0000
		Lateral 1	Inner Marginal 3				1.0000
		Outer Marginal 1	Inner Marginal 1				1.0000
		Outer Marginal 1	Inner Marginal 3				1.0000
		Outer Marginal 1	Lateral 1				1.0000
		Outer Marginal 3	Inner Marginal 1				1.0000
		Outer Marginal 3	Inner Marginal 3				1.0000
		Outer Marginal 3	Lateral 1				1.0000
		Outer Marginal 3	Outer Marginal 1				1.0000
		Inner Marginal 3	Central 2				0.5151
		Inner Marginal 3	Inner Marginal 2				0.3642
		Outer Marginal 3	Outer Marginal 2				0.2888
		Outer Marginal 2	Central 2				0.2835
		Outer Marginal 2	Inner Marginal 2				0.2850
		Lateral 1	Central 2				0.2072
		Lateral 1	Inner Marginal 2				0.2113
		Outer Marginal 1	Central 2				0.2072
		Outer Marginal 1	Inner Marginal 2				0.2113
		Outer Marginal 3	Central 2				0.2072
		Outer Marginal 3	Inner Marginal 2				0.2113
		Lateral 2	Central 2				0.1576
		Inner Marginal 1	Central 2				0.0269*
		Lateral 2	Inner Marginal 2				0.0477*
		Outer Marginal 1	Lateral 2				0.1340
		Outer Marginal 3	Lateral 2				0.1340
		Inner Marginal 3	Central 3				0.1002
		Inner Marginal 2	Central 3				0.0284*
		Lateral 1	Central 3				0.1167
		Outer Marginal 1	Central 3				0.1167
		Outer Marginal 3	Central 3				0.1167
		Outer Marginal 2	Central 3				0.0047*
		Inner Marginal 1	Central 3				0.0071*
		Lateral 3	Central 3				0.0559
		Lateral 2	Central 3				<.0001*
		Outer Marginal 2	Lateral 3				0.0692
		Outer Marginal 1	Lateral 3				0.0937
		Outer Marginal 3	Lateral 3				0.0937
		Lateral 3	Central 1				0.0023*
		Lateral 3	Inner Marginal 1				0.0026*
		Lateral 3	Lateral 1				0.0974
		Central 3	Central 1				0.0018*
		Lateral 2	Central 1				0.0053*
		Lateral 2	Inner Marginal 1				0.0056*
		Outer Marginal 3	Inner Marginal 1				0.0053*
		Lateral 3	Lateral 2				0.2121
		Outer Marginal 2	Inner Marginal 1				0.0058*
		Outer Marginal 3	Central 1				0.0087*
		Outer Marginal 2	Central 1				0.0053*
		Central 2	Central 1				0.0073*
		Lateral 3	Central 2				0.2976
		Outer Marginal 2	Outer Marginal 1				0.0084*
		Lateral 3	Central 3				0.2926
		Outer Marginal 3	Outer Marginal 1				0.0272*
		Outer Marginal 2	Lateral 3				0.3855
	s	Inner Marginal 3	Inner Marginal 1	29.7225	11	0.0018*	0.0413*
		Outer Marginal 3	Lateral 1				0.0645
		Outer Marginal 2	Lateral 2				0.1499
		Inner Marginal 3	Central 1				0.0755
		Inner Marginal 2	Inner Marginal 1				0.0840
		Outer Marginal 3	Central 3				0.1555
		Outer Marginal 2	Lateral 1				0.0991
		Inner Marginal 2	Central 1				0.1274
		Lateral 2	Lateral 1				0.2455
		Outer Marginal 3	Lateral 2				0.2454
		Outer Marginal 3	Lateral 3				0.5303
		Outer Marginal 3	Central 2				0.2060
		Outer Marginal 2	Central 3				0.3020
		Outer Marginal 2	Central 2				0.3047
		Inner Marginal 3	Central 3				0.4088
		Inner Marginal 3	Central 2				0.4052
		Outer Marginal 2	Inner Marginal 2				0.4277

		Outer Marginal 1	Inner Marginal 1				0.4647
		Lateral 3	Inner Marginal 2				0.8030
		Outer Marginal 2	Inner Marginal 3				0.6920
		Lateral 2	Central 2				0.7653
		Outer Marginal 3	Inner Marginal 2				0.7949
		Inner Marginal 3	Inner Marginal 2				0.8170
		Inner Marginal 2	Central 2				0.8290
		Lateral 1	Central 1				0.8990
		Outer Marginal 1	Lateral 1				0.9072
		Central 3	Central 2				0.9280
		Lateral 1	Inner Marginal 1				0.9145
		Inner Marginal 2	Central 3				0.9493
		Lateral 2	Central 3				0.9775
		Outer Marginal 1	Central 1				1.0000
		Inner Marginal 1	Central 1				0.7466
		Lateral 2	Inner Marginal 2				0.7216
		Outer Marginal 3	Outer Marginal 2				0.5974
		Outer Marginal 3	Inner Marginal 3				0.6031
		Lateral 1	Central 2				0.3393
		Lateral 3	Inner Marginal 3				0.6407
		Lateral 1	Inner Marginal 2				0.2721
		Lateral 1	Central 3				0.2992
		Outer Marginal 1	Inner Marginal 2				0.2011
		Lateral 2	Inner Marginal 3				0.3117
		Lateral 1	Inner Marginal 3				0.1190
		Outer Marginal 1	Inner Marginal 3				0.0814
		Outer Marginal 1	Central 2				0.0307*
		Inner Marginal 1	Central 2				0.0129*
		Outer Marginal 1	Central 3				0.0112*
		Outer Marginal 1	Lateral 2				0.0165*
		Inner Marginal 1	Central 3				0.0039*
		Outer Marginal 1	Lateral 3				0.0078*
		Lateral 3	Inner Marginal 1				0.0013*
		Lateral 3	Central 1				0.0043*
		Lateral 3	Lateral 1				0.0167*
		Central 3	Central 1				0.0006*
		Lateral 3	Inner Marginal 3				0.4608
		Central 2	Central 1				0.0014*
		Inner Marginal 2	Central 1				0.0017*
		Lateral 2	Inner Marginal 1				0.0025*
		Inner Marginal 2	Inner Marginal 1				0.0028*
		Lateral 2	Lateral 1				0.0099*
		Lateral 2	Central 1				0.0079*
		Outer Marginal 2	Inner Marginal 1				0.0086*
		Outer Marginal 2	Outer Marginal 1				0.0104*
		Outer Marginal 2	Central 1				0.0118*
		Outer Marginal 2	Lateral 1				0.0567
		Lateral 2	Inner Marginal 2				0.1700
		Lateral 1	Inner Marginal 1				0.1690
		Lateral 2	Inner Marginal 3				0.2375
		Outer Marginal 2	Lateral 3				0.7505
		Lateral 2	Central 3				0.6039
		Outer Marginal 1	Inner Marginal 1				0.4798
		Lateral 1	Central 1				0.7030
		Outer Marginal 2	Inner Marginal 3				0.7688
		Lateral 2	Central 2				0.8701
		Lateral 1	Inner Marginal 3				0.9142
		Inner Marginal 3	Central 1				1.0000
		Inner Marginal 3	Inner Marginal 1				1.0000
		Outer Marginal 1	Central 1				1.0000
		Outer Marginal 1	Inner Marginal 3				1.0000
		Outer Marginal 2	Inner Marginal 2				0.9576
	CI	Inner Marginal 1	Central 1	46.4523	11	<.0001*	0.8287
		Outer Marginal 3	Inner Marginal 3				0.5403
		Central 3	Central 2				0.7412
		Inner Marginal 3	Inner Marginal 2				0.6335
		Outer Marginal 2	Central 2				0.4937
		Lateral 3	Inner Marginal 2				0.7959
		Outer Marginal 1	Lateral 1				0.4033
		Outer Marginal 2	Central 3				0.5243
		Inner Marginal 3	Central 2				0.3893
		Outer Marginal 2	Lateral 2				0.3143
		Inner Marginal 2	Central 2				0.3057
		Outer Marginal 3	Central 1				0.2030
		Outer Marginal 3	Inner Marginal 1				0.1851
		Outer Marginal 3	Outer Marginal 1				0.1851
		Outer Marginal 3	Outer Marginal 2				0.1904
		Inner Marginal 2	Central 3				0.2884
		Inner Marginal 3	Central 3				0.3608
		Outer Marginal 3	Inner Marginal 2				0.1586
		Outer Marginal 3	Lateral 2				0.1637
		Outer Marginal 3	Central 2				0.1547
		Outer Marginal 3	Lateral 1				0.1528
		Lateral 1	Inner Marginal 2				0.0363*
		Outer Marginal 1	Inner Marginal 2				0.0089*
		Outer Marginal 1	Lateral 2				0.0042*
		Lateral 1	Central 2				0.0058*
		Outer Marginal 1	Central 2				0.0015*
		Inner Marginal 1	Central 2				0.0013*
		Lateral 3	Central 3				0.1264
		Lateral 3	Central 2				0.1584
		Outer Marginal 3	Central 3				0.1167
		Lateral 1	Central 3				0.0019*
		Lateral 3	Lateral 2				0.1390

		Outer Marginal 1	Central 3				0.0004*
		Inner Marginal 1	Central 3				0.0003*
		Outer Marginal 1	Lateral 3				0.0040*
		Outer Marginal 3	Lateral 3				0.0936
	Ca	Lateral 3	Lateral 1	74.5733	10	<.0001*	<.0001*
		Lateral 3	Inner Marginal 1				0.0198*
		Lateral 3	Inner Marginal 3				0.0003*
		Lateral 3	Central 2				0.0026*
		Lateral 2	Lateral 1				<.0001*
		Central 3	Central 2				<.0001*
		Lateral 2	Inner Marginal 1				0.0242*
		Lateral 2	Inner Marginal 3				0.0039*
		Outer Marginal 3	Lateral 1				0.0003*
		Outer Marginal 2	Lateral 1				0.0007*
		Lateral 2	Central 2				0.0273*
		Lateral 3	Inner Marginal 2				0.2766
		Inner Marginal 2	Inner Marginal 1				0.0358*
		Inner Marginal 3	Inner Marginal 1				0.0358*
		Outer Marginal 3	Inner Marginal 1				0.0354*
		Outer Marginal 3	Outer Marginal 1				0.0354*
		Outer Marginal 2	Inner Marginal 1				0.0413*
		Outer Marginal 2	Outer Marginal 1				0.0413*
		Inner Marginal 2	Central 2				0.0781
		Lateral 2	Inner Marginal 2				0.4240
		Outer Marginal 2	Inner Marginal 3				0.3225
		Outer Marginal 2	Central 2				0.5310
		Outer Marginal 1	Lateral 1				0.7934
		Outer Marginal 1	Inner Marginal 1				0.6985
		Lateral 3	Lateral 2				0.9513
		Lateral 1	Inner Marginal 1				1.0000
		Outer Marginal 2	Inner Marginal 2				0.5310
		Inner Marginal 3	Central 2				0.5067
		Outer Marginal 3	Inner Marginal 3				0.1744
		Outer Marginal 2	Lateral 2				0.2813
		Outer Marginal 3	Central 2				0.1656
		Outer Marginal 3	Outer Marginal 2				0.1060
		Inner Marginal 1	Central 2				0.0358*
		Outer Marginal 1	Central 2				0.0358*
		Outer Marginal 1	Inner Marginal 2				0.0358*
		Outer Marginal 1	Inner Marginal 3				0.0358*
		Outer Marginal 2	Lateral 3				0.2942
		Inner Marginal 3	Inner Marginal 2				0.0111*
		Lateral 2	Central 3				0.0559
		Outer Marginal 2	Central 3				0.0121*
		Outer Marginal 3	Inner Marginal 2				0.0013*
		Inner Marginal 2	Central 3				0.0064*
		Lateral 1	Central 2				0.0002*
		Lateral 1	Inner Marginal 2				0.0002*
		Lateral 1	Inner Marginal 3				0.0002*
		Inner Marginal 1	Central 3				0.0259*
		Outer Marginal 1	Central 3				0.0259*
		Outer Marginal 1	Lateral 2				0.0242*
		Outer Marginal 3	Lateral 2				0.0007*
		Lateral 1	Central 3				<.0001*
		Inner Marginal 3	Central 3				<.0001*
		Outer Marginal 3	Central 3				<.0001*
		Lateral 3	Central 3				0.0015*
		Outer Marginal 3	Lateral 3				<.0001*
		Outer Marginal 1	Lateral 3				0.0198*
	**All elements**	Lateral 3	Lateral 1	135.5405	11	<.0001*	<.0001*
		Lateral 3	Inner Marginal 1				<.0001*
		Lateral 3	Central 1				<.0001*
		Lateral 3	Inner Marginal 3				<.0001*
		Lateral 3	Central 2				0.0001*
		Lateral 2	Lateral 1				<.0001*
		Lateral 2	Inner Marginal 1				<.0001*
		Lateral 3	Lateral 2				0.0092*
		Lateral 2	Central 1				<.0001*
		Lateral 3	Inner Marginal 2				0.0263*
		Central 3	Central 2				<.0001*
		Central 3	Central 1				0.0001*
		Lateral 2	Inner Marginal 3				0.0013*
		Inner Marginal 2	Inner Marginal 1				0.0001*
		Inner Marginal 3	Inner Marginal 1				0.0001*
		Outer Marginal 3	Inner Marginal 1				0.0001*
		Outer Marginal 2	Lateral 1				0.0003*
		Outer Marginal 3	Outer Marginal 1				0.0002*
		Outer Marginal 2	Inner Marginal 1				0.0003*
		Central 2	Central 1				0.0004*
		Inner Marginal 2	Central 1				0.0004*
		Inner Marginal 3	Central 1				0.0004*
		Outer Marginal 3	Central 1				0.0004*
		Outer Marginal 3	Lateral 1				0.0016*
		Outer Marginal 2	Outer Marginal 1				0.0004*
		Outer Marginal 2	Central 1				0.0008*
		Lateral 1	Central 1				0.0200*
		Outer Marginal 2	Inner Marginal 3				0.0518
		Lateral 1	Inner Marginal 1				0.0942
		Inner Marginal 2	Central 2				0.2252
		Lateral 2	Central 2				0.3944
		Outer Marginal 1	Inner Marginal 1				0.3603
		Outer Marginal 1	Central 1				0.3537
		Inner Marginal 1	Central 1				1.0000
		Outer Marginal 2	Central 2				0.9212

		Lateral 2	Inner Marginal 2				0.8620
		Outer Marginal 3	Inner Marginal 3				0.5834
		Outer Marginal 2	Inner Marginal 2				0.4681
		Outer Marginal 1	Lateral 1				0.2797
		Outer Marginal 2	Lateral 2				0.3887
		Outer Marginal 3	Outer Marginal 2				0.0068*
		Inner Marginal 3	Inner Marginal 2				0.0035*
		Inner Marginal 3	Central 2				0.0024*
		Lateral 1	Inner Marginal 3				0.0009*
		Outer Marginal 1	Central 2				0.0002*
		Outer Marginal 1	Inner Marginal 2				0.0002*
		Outer Marginal 1	Inner Marginal 3				0.0002*
		Inner Marginal 1	Central 2				0.0001*
		Outer Marginal 3	Inner Marginal 2				0.0002*
		Outer Marginal 3	Central 2				0.0002*
		Inner Marginal 2	Central 3				0.0015*
		Outer Marginal 2	Central 3				0.0010*
		Lateral 1	Inner Marginal 2				<.0001*
		Lateral 1	Central 2				<.0001*
		Lateral 3	Central 3				0.0282*
		Lateral 2	Central 3				0.0005*
		Outer Marginal 1	Central 3				<.0001*
		Inner Marginal 3	Central 3				<.0001*
		Inner Marginal 1	Central 3				<.0001*
		Outer Marginal 3	Lateral 2				<.0001*
		Outer Marginal 1	Lateral 2				<.0001*
		Lateral 1	Central 3				<.0001*
		Outer Marginal 3	Central 3				<.0001*
		Outer Marginal 2	Lateral 3				0.0052*
		Outer Marginal 1	Lateral 3				<.0001*
		Outer Marginal 3	Lateral 3				<.0001*
		Lateral 3	Central 4				0.3447
		Lateral 4	Central 4				0.2925
		Marginal 4	Lateral 3				0.0678
	**F**	Lateral 4	Lateral 3	9.0759	3	0.0283*	0.1400
		Marginal 4	Lateral 4				0.0267*
		Marginal 4	Central 4				0.0258*
		Lateral 3	Central 4				<.0001*
		Marginal 2	Central 4				0.0008*
		Lateral 2	Central 4				0.0074*
		Marginal 3	Central 4				0.0208*
		Lateral 3	Central 2				0.0072*
		Lateral 3	Central 1				0.1644
		Lateral 3	Lateral 1				0.0254*
		Lateral 4	Central 4				0.0245*
		Lateral 4	Central 1				0.1253
		Marginal 2	Lateral 4				0.0173*
		Marginal 3	Lateral 4				0.0652
		Central 3	Central 2				0.0359*
		Central 2	Central 1				0.1547
		Marginal 3	Marginal 1				0.0287*
		Marginal 2	Central 1				0.1625
		Marginal 3	Lateral 3				0.2553
		Lateral 3	Lateral 2				0.2131
		Marginal 2	Marginal 1				0.0887
		Marginal 3	Central 2				0.1039
		Marginal 2	Lateral 1				0.1218
		Lateral 4	Lateral 1				0.2768
		Lateral 2	Lateral 1				0.0882
		Central 3	Central 1				0.1904
		Lateral 2	Central 1				0.1904
		Marginal 3	Lateral 1				0.0606
Littorina littorea		Central 4	Central 1				0.6507
		Marginal 3	Marginal 2				0.1891
		Marginal 3	Lateral 2				0.1564
	**Na**	Marginal 1	Central 1	58.9856	11	<.0001*	0.2636
		Marginal 3	Central 3				0.1849
		Lateral 2	Central 2				0.2828
		Marginal 2	Central 2				0.3268
		Marginal 3	Central 1				0.2888
		Lateral 3	Central 3				0.5820
		Marginal 1	Central 4				0.7209
		Lateral 1	Central 1				0.4682
		Lateral 1	Central 4				0.9150
		Marginal 1	Lateral 1				0.8455
		Marginal 2	Lateral 2				0.9155
		Lateral 2	Central 3				0.6544
		Marginal 2	Central 3				0.6711
		Marginal 4	Central 1				0.2113
		Marginal 2	Lateral 3				0.3495
		Lateral 1	Central 2				0.2026
		Marginal 1	Lateral 4				0.2998
		Marginal 1	Central 2				0.1861
		Lateral 1	Central 3				0.0472*
		Marginal 1	Lateral 2				0.0537
		Marginal 4	Lateral 1				0.0142*
		Marginal 4	Marginal 3				0.0142*
		Marginal 1	Central 3				0.0210*
		Lateral 4	Central 2				0.0612
		Marginal 4	Central 3				0.0034*
		Marginal 4	Lateral 2				0.0034*
		Marginal 4	Marginal 1				0.0033*
		Lateral 4	Lateral 2				0.0324*
		Marginal 4	Marginal 2				0.0018*

		Marginal 4	Central 2				0.0014*
		Lateral 4	Central 3				0.0036*
		Marginal 1	Lateral 3				0.0064*
		Central 4	Central 2				0.0028*
		Marginal 4	Lateral 4				0.0005*
		Central 4	Central 3				0.0008*
		Marginal 4	Lateral 3				0.0003*
		Lateral 4	Lateral 3				<.0001*
		Marginal 4	Central 4				0.0002*
		Central 4	Central 2				0.0184*
		Central 4	Central 3				0.0065*
		Lateral 4	Lateral 3				0.0151*
		Central 4	Central 1				0.0402*
		Marginal 1	Lateral 3				0.0705
		Marginal 1	Central 2				0.0216*
		Lateral 3	Central 2				0.2693
		Lateral 4	Central 3				0.0536
		Lateral 4	Central 2				0.1047
		Marginal 1	Central 3				0.0459*
		Lateral 4	Central 1				0.1367
		Lateral 4	Lateral 1				0.1723
		Marginal 1	Central 1				0.1482
		Lateral 1	Central 2				0.0919
		Central 3	Central 2				0.1836
		Lateral 1	Central 3				0.2714
		Lateral 1	Central 1				0.4537
		Marginal 1	Lateral 1				0.7228
		Marginal 2	Central 2				0.8571
		Marginal 4	Marginal 3				1.0000
		Lateral 3	Central 3				0.9543
		Central 3	Central 1				0.8257
		Marginal 2	Central 3				0.5515
		Marginal 3	Central 2				0.3711
		Marginal 2	Central 1				0.4587
		Marginal 3	Marginal 2				0.2888
		Lateral 3	Central 1				0.6329
	**Mg**	Marginal 2	Lateral 1	50.4065	10	<.0001*	0.2395
		Marginal 3	Central 1				0.2348
		Central 2	Central 1				0.1337
		Marginal 3	Lateral 1				0.2072
		Marginal 2	Lateral 3				0.5116
		Marginal 3	Central 3				0.1734
		Lateral 3	Lateral 1				0.3538
		Lateral 4	Central 4				0.2633
		Marginal 1	Lateral 4				0.1190
		Marginal 2	Marginal 1				0.0776
		Marginal 2	Lateral 4				0.1187
		Marginal 4	Central 2				0.1183
		Marginal 3	Marginal 1				0.1340
		Marginal 3	Lateral 4				0.1840
		Marginal 4	Marginal 2				0.0498*
		Lateral 1	Central 4				0.0517
		Marginal 1	Central 4				0.0269*
		Marginal 4	Central 1				0.0111*
		Marginal 4	Central 3				0.0042*
		Marginal 4	Lateral 1				0.0041*
		Marginal 2	Central 4				0.0180*
		Marginal 3	Lateral 3				0.1684
		Marginal 3	Central 4				0.1103
		Marginal 4	Lateral 4				0.0003*
		Marginal 4	Marginal 1				0.0001*
		Marginal 4	Lateral 3				0.0003*
		Lateral 3	Central 4				0.0001*
		Marginal 4	Central 4				<.0001*
		Lateral 3	Lateral 2				0.0251*
		Lateral 3	Central 1				0.1026
		Lateral 3	Central 2				0.1026
		Lateral 3	Lateral 1				0.1026
		Lateral 3	Central 3				0.0113*
		Central 4	Central 1				0.1108
		Central 4	Central 2				0.1108
		Central 4	Central 3				0.0076*
		Lateral 4	Lateral 2				0.0294*
		Lateral 4	Central 1				0.1258
		Lateral 4	Central 2				0.1258
		Lateral 4	Lateral 1				0.1258
		Marginal 4	Central 2				0.1253
		Marginal 4	Marginal 2				0.1253
	**Si**	Marginal 3	Lateral 2	58.0894	11	<.0001*	0.0321*
		Marginal 3	Central 1				0.1325
		Marginal 3	Central 2				0.1325
		Marginal 3	Lateral 1				0.1325
		Marginal 3	Marginal 1				0.1325
		Marginal 3	Marginal 2				0.1325
		Marginal 4	Central 1				0.2610
		Marginal 4	Lateral 1				0.2610
		Marginal 4	Marginal 1				0.2610
		Marginal 3	Central 3				0.1270
		Lateral 4	Central 3				0.2233
		Marginal 4	Lateral 2				0.4392
		Central 3	Central 1				0.2113
		Central 3	Central 2				0.2113
		Lateral 2	Central 2				0.5403
		Marginal 3	Lateral 4				0.8190

		Central 2	Central 1				1.0000
		Lateral 1	Central 1				1.0000
		Lateral 1	Central 2				1.0000
		Lateral 2	Central 1				1.0000
		Lateral 2	Lateral 1				1.0000
		Marginal 1	Central 1				1.0000
		Marginal 1	Central 2				1.0000
		Marginal 1	Lateral 1				1.0000
		Marginal 1	Lateral 2				1.0000
		Marginal 2	Central 1				1.0000
		Marginal 2	Central 2				1.0000
		Marginal 2	Lateral 1				1.0000
		Marginal 2	Marginal 1				1.0000
		Marginal 2	Lateral 2				0.5403
		Lateral 3	Central 4				0.6918
		Lateral 2	Central 3				0.2433
		Lateral 1	Central 3				0.2113
		Marginal 1	Central 3				0.2113
		Marginal 2	Central 3				0.2113
		Marginal 3	Lateral 3				0.2131
		Marginal 3	Central 4				0.1421
		Marginal 4	Central 3				0.0504
		Lateral 4	Central 4				0.0586
		Lateral 4	Lateral 3				0.0900
		Marginal 1	Lateral 4				0.1258
		Marginal 2	Lateral 4				0.1258
		Lateral 1	Central 4				0.1108
		Marginal 1	Central 4				0.1108
		Marginal 2	Central 4				0.1108
		Lateral 2	Central 4				0.0237*
		Marginal 4	Marginal 3				<.0001*
		Marginal 4	Lateral 4				<.0001*
		Marginal 1	Lateral 3				0.1026
		Marginal 2	Lateral 3				0.1026
		Marginal 4	Central 4				<.0001*
		Marginal 4	Lateral 3				<.0001*
		Lateral 3	Central 2				0.0024*
		Lateral 3	Lateral 1				0.0214*
		Lateral 3	Central 1				0.1042
		Lateral 3	Lateral 2				0.0091*
		Central 4	Central 2				0.0035*
		Central 4	Central 1				0.1108
		Marginal 4	Central 2				0.0043*
		Lateral 4	Lateral 2				0.0015*
		Marginal 4	Marginal 1				0.0032*
		Lateral 4	Central 2				0.0029*
		Marginal 4	Lateral 1				0.0275*
		Marginal 4	Central 1				0.1207
		Lateral 4	Lateral 1				0.0294*
		Lateral 4	Central 1				0.1256
		Marginal 4	Marginal 2				0.0403*
		Central 4	Central 3				0.0764
		Marginal 4	Lateral 2				0.1089
		Central 3	Central 2				0.0107*
		Marginal 3	Marginal 1				0.0175*
		Marginal 3	Marginal 2				0.0195*
		Marginal 3	Lateral 2				0.0365*
		Marginal 3	Central 2				0.0294*
		Lateral 4	Lateral 3				0.4060
		Central 3	Central 1				0.1904
		Marginal 2	Marginal 1				0.0714
		Lateral 2	Central 2				0.0662
		Lateral 4	Central 3				0.3003
		Lateral 2	Lateral 1				0.0814
	P	Marginal 2	Lateral 1	55.8509	11	<.0001*	0.1180
		Marginal 2	Central 2				0.1400
		Marginal 3	Lateral 1				0.1002
		Lateral 2	Central 1				0.2416
		Marginal 2	Central 1				0.2416
		Lateral 3	Central 3				0.6650
		Central 2	Central 1				0.2888
		Marginal 3	Central 1				0.2765
		Marginal 1	Central 1				0.7290
		Lateral 1	Central 1				1.0000
		Marginal 1	Lateral 1				1.0000
		Marginal 2	Lateral 2				0.7533
		Lateral 4	Central 4				0.6487
		Lateral 1	Central 2				0.1588
		Marginal 1	Central 2				0.2109
		Marginal 3	Central 3				0.2183
		Marginal 4	Marginal 3				0.4692
		Marginal 1	Lateral 2				0.0714
		Lateral 1	Central 3				0.0570
		Marginal 3	Lateral 3				0.3506
		Lateral 2	Central 3				0.0094*
		Lateral 3	Central 4				0.2059
		Marginal 1	Central 3				0.0054*
		Marginal 2	Central 3				0.0058*
		Marginal 4	Central 3				0.0742
		Marginal 3	Lateral 4				0.0652
		Marginal 3	Central 4				0.0819
		Marginal 4	Lateral 3				0.0302*
		Marginal 4	Lateral 4				0.0030*
		Marginal 1	Lateral 4				0.0011*

		Marginal 2	Lateral 4				0.0011*
		Marginal 4	Central 4				0.0016*
		Lateral 2	Central 4				0.0035*
		Lateral 1	Central 4				0.0237*
		Marginal 2	Central 4				0.0020*
		Marginal 1	Central 4				0.0009*
		Marginal 2	Lateral 3				0.0042*
		Marginal 1	Lateral 3				0.0010*
		Lateral 3	Central 4				<.0001*
		Marginal 2	Central 4				<.0001*
		Marginal 1	Central 4				<.0001*
		Lateral 4	Central 4				<.0001*
		Marginal 3	Central 4				<.0001*
		Lateral 3	Lateral 1				0.0038*
		Lateral 2	Central 4				<.0001*
		Lateral 3	Central 1				0.0124*
		Lateral 1	Central 4				0.0002*
		Marginal 2	Lateral 4				<.0001*
		Marginal 1	Lateral 4				<.0001*
		Marginal 3	Lateral 4				<.0001*
		Marginal 2	Marginal 1				0.0056*
		Marginal 2	Lateral 1				0.0109*
		Lateral 2	Lateral 1				0.0037*
		Marginal 3	Marginal 1				0.0319*
		Central 2	Central 1				0.0174*
		Marginal 2	Central 1				0.0611
		Marginal 3	Lateral 1				0.0786
		Lateral 2	Central 1				0.0481*
		Marginal 3	Central 1				0.1408
		Central 3	Central 1				0.3083
		Marginal 1	Central 1				0.8817
		Lateral 1	Central 1				1.0000
		Marginal 1	Lateral 1				1.0000
		Marginal 3	Marginal 2				1.0000
		Marginal 3	Central 3				0.7242
	**S**	Marginal 2	Lateral 3	105.7584	10	<.0001*	0.8037
		Lateral 3	Central 3				0.7777
		Marginal 3	Lateral 3				0.7553
		Marginal 3	Lateral 2				0.4828
		Marginal 2	Central 3				0.5579
		Lateral 2	Central 3				0.5407
		Lateral 2	Central 2				0.2829
		Marginal 2	Lateral 2				0.2934
		Marginal 3	Central 2				0.2504
		Lateral 3	Lateral 2				0.5331
		Central 3	Central 2				0.2688
		Marginal 2	Central 2				0.1642
		Lateral 1	Central 3				0.0741
		Marginal 1	Central 3				0.0541
		Lateral 1	Central 2				0.0066*
		Lateral 3	Central 2				0.1373
		Marginal 1	Lateral 2				0.0016*
		Marginal 1	Central 2				0.0008*
		Lateral 4	Central 1				0.0011*
		Lateral 4	Lateral 1				0.0005*
		Lateral 4	Lateral 2				<.0001*
		Lateral 4	Central 2				<.0001*
		Central 4	Central 1				0.0006*
		Lateral 4	Central 3				<.0001*
		Central 4	Central 2				<.0001*
		Marginal 1	Lateral 3				0.0002*
		Central 4	Central 3				<.0001*
		Lateral 4	Lateral 3				<.0001*
		Lateral 3	Central 4				0.0274*
		Marginal 3	Lateral 3				0.0151*
		Marginal 1	Central 4				0.0437*
		Marginal 3	Central 2				0.0399*
		Marginal 2	Central 4				0.0896
		Marginal 2	Lateral 3				0.2091
		Marginal 3	Central 3				0.1685
		Marginal 2	Central 2				0.1812
		Central 3	Central 2				0.2447
		Lateral 1	Central 4				0.0814
		Marginal 3	Marginal 1				0.1878
		Lateral 2	Central 4				0.1052
		Marginal 3	Central 4				0.1052
		Marginal 3	Central 1				0.1939
		Marginal 3	Marginal 2				0.3502
	**Cl**	Lateral 1	Central 2	12.0432	9	0.2109	0.4243
		Marginal 2	Marginal 1				0.4995
		Marginal 3	Lateral 1				0.3913
		Marginal 1	Lateral 2				0.5876
		Marginal 3	Lateral 2				0.4705
		Lateral 2	Central 2				0.6198
		Marginal 1	Central 2				0.6819
		Lateral 3	Central 2				0.7811
		Central 3	Central 1				0.7437
		Marginal 2	Central 3				0.7724
		Marginal 2	Central 1				0.7336
		Marginal 2	Lateral 2				0.7989
		Lateral 1	Central 1				1.0000
		Lateral 1	Central 3				1.0000
		Lateral 3	Lateral 2				1.0000
		Marginal 1	Lateral 3				1.0000

		Marginal 2	Lateral 1				1.0000
		Lateral 2	Central 3				0.9479
		Lateral 3	Central 1				0.8926
		Central 2	Central 1				0.7766
		Lateral 2	Central 1				0.6650
		Marginal 1	Central 1				0.6393
		Marginal 1	Lateral 1				0.6363
		Lateral 2	Lateral 1				0.5403
		Marginal 1	Central 3				0.6479
		Lateral 3	Lateral 1				0.5818
		Central 4	Central 1				0.1052
		Central 4	Central 2				0.1059
		Lateral 3	Central 3				0.1373
		Central 4	Central 3				0.0601
		Central 3	Central 2				0.1386
		Lateral 3	Central 1				0.3458
		Marginal 1	Central 2				0.3758
		Lateral 4	Central 1				0.4682
		Lateral 4	Central 2				0.5959
		Central 3	Central 1				0.4795
		Lateral 2	Central 1				0.4795
		Marginal 1	Lateral 3				0.6531
		Marginal 1	Central 1				0.6171
		Marginal 2	Central 1				0.7168
		Lateral 1	Central 2				0.7728
		Lateral 2	Central 2				0.7671
		Marginal 1	Central 3				0.7609
		Marginal 1	Lateral 2				0.7609
		Lateral 4	Lateral 1				0.8116
		Lateral 4	Lateral 3				0.8584
		Central 2	Central 1				1.0000
		Lateral 1	Central 1				1.0000
		Lateral 1	Central 3				1.0000
		Lateral 2	Lateral 1				1.0000
		Lateral 3	Lateral 1				1.0000
		Lateral 4	Central 3				1.0000
	**K**	Lateral 4	Lateral 2	10.6864	9	0.2978	1.0000
		Marginal 1	Lateral 1				1.0000
		Marginal 1	Lateral 4				1.0000
		Marginal 2	Lateral 1				0.8143
		Lateral 3	Central 2				0.6579
		Marginal 2	Lateral 4				0.6631
		Marginal 4	Central 1				0.5403
		Marginal 2	Central 2				0.4715
		Marginal 2	Lateral 3				0.3681
		Marginal 2	Marginal 1				0.3681
		Lateral 2	Central 3				0.1939
		Marginal 4	Central 3				0.2207
		Marginal 4	Lateral 1				0.2453
		Marginal 4	Lateral 2				0.2207
		Lateral 3	Central 3				0.1281
		Lateral 3	Lateral 2				0.1281
		Marginal 4	Central 2				0.1489
		Marginal 4	Lateral 3				0.1386
		Marginal 4	Marginal 1				0.1386
		Marginal 2	Central 3				0.0952
		Marginal 2	Lateral 2				0.0952
		Marginal 4	Lateral 4				0.1052
		Marginal 4	Marginal 2				0.1002
		Lateral 3	Central 2				0.0004*
		Lateral 3	Lateral 2				0.0010*
		Central 4	Central 2				<.0001*
		Lateral 4	Central 2				<.0001*
		Lateral 4	Lateral 3				0.0059*
		Central 3	Central 2				0.0001*
		Lateral 4	Lateral 2				0.0001*
		Marginal 1	Central 2				0.0002*
		Marginal 1	Lateral 2				0.0002*
		Central 4	Central 1				0.0166*
		Marginal 3	Lateral 2				0.0015*
		Lateral 4	Central 1				0.0044*
		Marginal 3	Central 2				0.0034*
		Central 3	Central 1				0.0187*
		Lateral 4	Lateral 1				0.0182*
		Lateral 1	Central 2				0.0088*
		Marginal 2	Lateral 2				0.0241*
	**Ca**	Marginal 3	Marginal 2	78.4864	11	<.0001*	0.0627
		Marginal 2	Central 2				0.0662
		Lateral 3	Central 1				0.4052
		Lateral 3	Lateral 1				0.7009
		Marginal 1	Central 1				0.5187
		Marginal 4	Lateral 2				0.7071
		Lateral 1	Central 1				0.6466
		Marginal 3	Lateral 1				0.7727
		Central 4	Central 3				0.8323
		Marginal 1	Lateral 1				1.0000
		Marginal 3	Central 1				1.0000
		Marginal 4	Central 2				1.0000
		Marginal 3	Marginal 1				0.9806
		Marginal 3	Lateral 3				0.9598
		Lateral 4	Central 3				0.7320
		Lateral 2	Central 2				0.5081
		Lateral 4	Central 4				0.4644
		Marginal 1	Lateral 3				0.5398

		Marginal 4	Marginal 2				0.2318
		Marginal 2	Lateral 1				0.0694
		Marginal 2	Central 1				0.0464*
		Marginal 3	Lateral 4				0.0558
		Marginal 4	Lateral 1				0.0319*
		Lateral 2	Lateral 1				0.0055*
		Marginal 3	Central 3				0.0542
		Lateral 2	Central 1				0.0043*
		Marginal 4	Central 1				0.0296*
		Lateral 1	Central 3				0.0299*
		Central 2	Central 1				0.0046*
		Marginal 4	Marginal 3				0.0158*
		Marginal 2	Marginal 1				0.0052*
		Marginal 3	Central 4				0.0161*
		Marginal 4	Marginal 1				0.0020*
		Marginal 1	Central 3				0.0036*
		Lateral 1	Central 4				0.0138*
		Marginal 1	Lateral 4				0.0007*
		Lateral 2	Central 3				0.0005*
		Marginal 4	Central 3				0.0004*
		Marginal 1	Central 4				0.0012*
		Marginal 2	Central 3				0.0002*
		Marginal 4	Lateral 4				<.0001*
		Marginal 2	Lateral 3				0.0066*
		Lateral 3	Central 3				0.0054*
		Marginal 4	Central 4				0.0002*
		Marginal 2	Lateral 4				<.0001*
		Lateral 2	Central 4				0.0002*
		Marginal 2	Central 4				<.0001*
		Marginal 4	Lateral 3				0.0012*
		Lateral 3	Central 4				0.0008*
		Central 4	Central 2				<.0001*
		Lateral 3	Central 2				0.0169*
		Central 4	Central 3				0.0091*
		Lateral 4	Lateral 3				0.0337*
		Lateral 3	Lateral 2				0.0571
		Lateral 4	Central 2				0.0077*
		Lateral 4	Lateral 2				0.0084*
		Marginal 3	Central 2				0.0100*
		Marginal 3	Lateral 2				0.0268*
		Central 3	Central 2				0.0615
		Lateral 4	Central 3				0.2826
		Marginal 3	Marginal 2				0.2807
		Marginal 3	Marginal 1				0.2715
		Marginal 2	Central 2				0.3318
		Lateral 4	Lateral 1				0.4212
		Lateral 1	Central 2				0.3532
		Marginal 3	Lateral 3				0.6527
		Marginal 1	Lateral 2				0.5551
		Marginal 2	Lateral 2				0.6626
		Marginal 1	Central 2				0.6784
		Lateral 1	Central 3				0.8130
		Marginal 3	Lateral 1				0.8647
		Lateral 2	Central 2				0.9231
		Marginal 3	Central 3				0.9650
		Marginal 2	Marginal 1				0.8255
		Marginal 1	Lateral 1				0.7414
		Lateral 1	Central 1				0.4745
		Marginal 1	Central 3				0.6847
		Marginal 1	Lateral 3				0.7094
		Marginal 2	Lateral 1				0.5319
		Central 4	Central 1				0.7361
	**Fe**	Lateral 4	Central 1	51.5682	11	<.0001*	0.6226
		Lateral 2	Lateral 1				0.3039
		Marginal 3	Lateral 4				0.4691
		Marginal 1	Central 1				0.2319
		Lateral 3	Lateral 1				0.5996
		Marginal 2	Lateral 3				0.4621
		Lateral 3	Central 3				0.3861
		Marginal 2	Central 3				0.2538
		Marginal 4	Central 1				0.0668
		Lateral 4	Central 4				0.2876
		Marginal 4	Lateral 1				0.0252*
		Lateral 2	Central 1				0.0495*
		Marginal 2	Central 1				0.1477
		Marginal 3	Central 1				0.1470
		Marginal 1	Lateral 4				0.1119
		Marginal 4	Central 2				0.0288*
		Marginal 4	Marginal 1				0.0127*
		Central 2	ittorina littorea Central 1				0.0446*
		Lateral 2	Central 3				0.0861
		Lateral 1	Central 4				0.2119
		Marginal 4	Lateral 2				0.0115*
		Marginal 2	Lateral 4				0.0591
		Central 3	Central 1				0.1056
		Marginal 4	Marginal 2				0.0083*
		Marginal 1	Central 4				0.0274*
		Marginal 4	Marginal 3				0.0011*
		Marginal 4	Central 3				0.0032*
		Marginal 3	Central 4				0.0072*
		Marginal 4	Lateral 4				0.0008*
		Marginal 2	Central 4				0.0019*
		Marginal 4	Central 4				0.0002*
		Lateral 2	Central 4				<.0001*

		Lateral 3	Central 1				0.0216*
		Marginal 4	Lateral 3				0.0008*
		Lateral 3	Central 4				<.0001*
	**All elements**	Lateral 3	Lateral 2	117.2615	11	<.0001*	<.0001*
		Lateral 3	Central 2				<.0001*
		Lateral 3	Central 1				0.0004*
		Lateral 3	Lateral 1				0.0002*
		Central 4	Central 2				<.0001*
		Central 4	Central 1				0.0006*
		Lateral 4	Central 2				<.0001*
		Central 3	Central 2				0.0002*
		Lateral 4	Lateral 2				0.0001*
		Lateral 4	Lateral 1				0.0006*
		Lateral 4	Central 1				0.0011*
		Central 3	Central 1				0.0022*
		Marginal 3	Marginal 1				0.0045*
		Marginal 3	Marginal 2				0.0063*
		Marginal 3	Lateral 2				0.0105*
		Marginal 3	Central 2				0.0148*
		Central 4	Central 3				0.0784
		Marginal 3	Central 1				0.0233*
		Marginal 3	Lateral 1				0.0354*
		Lateral 4	Central 3				0.1768
		Lateral 4	Lateral 3				0.3688
		Lateral 3	Central 3				0.5565
		Marginal 1	Central 1				0.4586
		Lateral 1	Central 1				0.3580
		Marginal 1	Lateral 2				0.5868
		Lateral 1	Central 2				0.7250
		Central 2	Central 1				0.8208
		Marginal 1	Central 2				0.9077
		Lateral 2	Central 1				0.8835
		Marginal 2	Central 1				0.8896
		Marginal 2	Central 2				0.7220
		Lateral 2	Central 2				0.6796
		Marginal 1	Lateral 1				0.6606
		Lateral 4	Central 4				0.7094
		Lateral 2	Lateral 1				0.5181
		Marginal 2	Lateral 2				0.6087
		Marginal 2	Marginal 1				0.3956
		Marginal 3	Central 3				0.3718
		Marginal 2	Lateral 1				0.3025
		Marginal 3	Lateral 4				0.0532
		Lateral 3	Central 4				0.1469
		Marginal 3	Lateral 3				0.1227
		Marginal 3	Central 4				0.0261*
		Marginal 4	Central 1				0.0041*
		Lateral 1	Central 3				0.0018*
		Marginal 4	Lateral 1				0.0015*
		Marginal 4	Lateral 2				0.0008*
		Lateral 2	Central 3				0.0002*
		Marginal 2	Central 3				0.0002*
		Marginal 4	Central 2				0.0001*
		Marginal 1	Central 3				<.0001*
		Marginal 4	Marginal 1				<.0001*
		Marginal 4	Marginal 3				<.0001*
		Marginal 4	Marginal 2				<.0001*
		Marginal 2	Lateral 4				<.0001*
		Marginal 1	Lateral 4				<.0001*
		Lateral 1	Central 4				0.0002*
		Marginal 4	Lateral 4				<.0001*
		Marginal 4	Central 3				<.0001*
		Lateral 2	Central 4				<.0001*
		Marginal 2	Central 4				<.0001*
		Marginal 1	Central 4				<.0001*
		Marginal 4	Central 4				<.0001*
		Marginal 2	Lateral 3				<.0001*
		Marginal 1	Lateral 3				<.0001*
		Marginal 4	Lateral 3				<.0001*
Reymondia horei	**Na**	Lateral 3	Inner Marginal 3	21.6336	10	0.0171*	0.0104*
		Lateral 3	Inner Marginal 2				0.0465*
		Lateral 2	Inner Marginal 3				0.0140*
		Outer Marginal 3	Inner Marginal 3				0.0141*
		Lateral 2	Inner Marginal 2				0.1065
		Outer Marginal 1	Inner Marginal 3				0.0275*
		Outer Marginal 1	Inner Marginal 2				0.0668
		Outer Marginal 2	Inner Marginal 3				0.0497*
		Outer Marginal 2	Inner Marginal 2				0.1052
		Lateral 2	Central 1				0.3471
		Central 3	Central 1				0.3156
		Central 2	Central 1				0.3496
		Lateral 3	Central 1				0.7107
		Inner Marginal 1	Central 3				0.5169
		Inner Marginal 1	Central 2				0.5486
		Outer Marginal 3	Inner Marginal 2				0.7473
		Outer Marginal 2	Outer Marginal 1				0.7491
		Inner Marginal 1	Central 1				0.8525
		Outer Marginal 3	Outer Marginal 1				1.0000
		Central 3	Central 2				0.8541
		Outer Marginal 3	Outer Marginal 2				0.8320
		Lateral 3	Lateral 2				0.8407
		Outer Marginal 2	Central 1				0.5354
		Outer Marginal 2	Inner Marginal 1				0.3725
		Lateral 2	Inner Marginal 1				0.5530

		Lateral 2	Central 3				0.4805
		Outer Marginal 3	Central 1				0.4869
		Outer Marginal 1	Inner Marginal 1				0.2976
		Inner Marginal 2	Inner Marginal 1				0.1386
		Inner Marginal 3	Inner Marginal 2				0.1386
		Lateral 2	Central 2				0.3579
		Outer Marginal 2	Lateral 2				0.3574
		Outer Marginal 1	Central 1				0.3156
		Outer Marginal 2	Central 2				0.1779
		Inner Marginal 3	Inner Marginal 1				0.0722
		Outer Marginal 2	Central 3				0.1658
		Outer Marginal 3	Inner Marginal 1				0.2712
		Outer Marginal 2	Lateral 3				0.5064
		Inner Marginal 2	Central 2				0.0814
		Inner Marginal 2	Central 3				0.0668
		Inner Marginal 3	Central 2				0.0358*
		Outer Marginal 1	Central 2				0.0552
		Outer Marginal 3	Central 2				0.0982
		Outer Marginal 1	Lateral 2				0.0925
		Outer Marginal 1	Central 3				0.0453*
		Inner Marginal 3	Central 3				0.0275*
		Outer Marginal 3	Lateral 2				0.0961
		Outer Marginal 3	Central 3				0.0735
		Lateral 3	Inner Marginal 1				0.3727
		Lateral 3	Central 3				0.2606
		Inner Marginal 2	Central 1				0.0442*
		Inner Marginal 3	Central 1				0.0157*
		Lateral 3	Central 2				0.1727
		Outer Marginal 1	Lateral 3				0.1402
		Outer Marginal 3	Lateral 3				0.1042
	**Mg**	Outer Marginal 3	Lateral 3	24.8909	10	0.0056*	0.0128*
		Outer Marginal 3	Outer Marginal 2				0.0105*
		Outer Marginal 1	Lateral 3				0.1263
		Outer Marginal 3	Lateral 2				0.0952
		Lateral 2	Inner Marginal 2				0.1172
		Outer Marginal 3	Inner Marginal 2				0.0440*
		Outer Marginal 1	Inner Marginal 2				0.1175
		Inner Marginal 1	Central 2				0.2699
		Outer Marginal 3	Central 2				0.3071
		Outer Marginal 3	Inner Marginal 3				0.2888
		Inner Marginal 3	Inner Marginal 2				0.3617
		Central 3	Central 2				0.3257
		Outer Marginal 3	Outer Marginal 1				0.4377
		Outer Marginal 1	Inner Marginal 3				0.5886
		Inner Marginal 1	Central 3				0.5169
		Inner Marginal 1	Central 1				0.7172
		Central 3	Central 1				0.7074
		Outer Marginal 3	Central 1				0.7665
		Lateral 2	Inner Marginal 3				0.8949
		Outer Marginal 1	Lateral 2				0.9676
		Outer Marginal 3	Central 3				0.9567
		Outer Marginal 1	Central 2				0.9674
		Inner Marginal 3	Central 2				0.7225
		Outer Marginal 3	Inner Marginal 1				0.6722
		Lateral 3	Inner Marginal 2				0.7850
		Inner Marginal 3	Inner Marginal 1				0.3725
		Central 2	Central 1				0.5519
		Outer Marginal 2	Lateral 3				0.7044
		Outer Marginal 1	Central 3				0.3763
		Outer Marginal 2	Inner Marginal 2				0.4855
		Inner Marginal 3	Central 3				0.1658
		Outer Marginal 1	Inner Marginal 1				0.2270
		Outer Marginal 1	Central 1				0.2700
		Lateral 2	Central 2				0.4039
		Inner Marginal 3	Central 1				0.2239
		Lateral 2	Central 1				0.3539
		Outer Marginal 2	Inner Marginal 3				0.2365
		Lateral 3	Inner Marginal 3				0.5060
		Inner Marginal 2	Central 2				0.1053
		Lateral 2	Inner Marginal 1				0.3317
		Inner Marginal 2	Inner Marginal 1				0.0960
		Outer Marginal 2	Outer Marginal 1				0.0473*
		Outer Marginal 2	Inner Marginal 1				0.0826
		Outer Marginal 2	Central 2				0.0447*
		Inner Marginal 2	Central 1				0.0224*
		Inner Marginal 2	Central 3				0.0113*
		Outer Marginal 2	Lateral 2				0.0445*
		Lateral 3	Lateral 2				0.1046
		Lateral 2	Central 3				0.0422*
		Outer Marginal 2	Central 3				0.0071*
		Outer Marginal 2	Central 1				0.0075*
		Lateral 3	Central 2				0.0795
		Lateral 3	Inner Marginal 1				0.1143
		Lateral 3	Central 1				0.0180*
		Lateral 3	Central 3				0.0151*
	**Si**	Inner Marginal 2	Central 1	9.2661	10	0.5071	0.0765
		Inner Marginal 2	Central 2				0.1498
		Inner Marginal 2	Central 3				0.1489
		Inner Marginal 2	Inner Marginal 1				0.1386
		Inner Marginal 3	Central 1				0.1386
		Inner Marginal 3	Central 3				0.2453
		Inner Marginal 3	Inner Marginal 1				0.2207
		Lateral 2	Central 2				0.4529
		Inner Marginal 1	Central 2				0.4745

		Inner Marginal 3	Central 2				0.4811
		Lateral 2	Central 1				0.6508
		Outer Marginal 3	Outer Marginal 2				0.5403
		Lateral 2	Central 3				0.6959
		Central 3	Central 2				0.8057
		Lateral 2	Inner Marginal 1				0.8451
		Lateral 2	Inner Marginal 3				0.8465
		Central 3	Central 1				1.0000
		Inner Marginal 1	Central 3				1.0000
		Lateral 3	Central 1				1.0000
		Lateral 3	Central 3				1.0000
		Lateral 3	Inner Marginal 1				1.0000
		Lateral 3	Inner Marginal 3				1.0000
		Outer Marginal 1	Lateral 3				1.0000
		Outer Marginal 2	Outer Marginal 1				1.0000
		Outer Marginal 3	Lateral 3				1.0000
		Lateral 3	Central 2				0.8642
		Inner Marginal 1	Central 1				0.7609
		Outer Marginal 2	Lateral 3				0.8432
		Outer Marginal 1	Central 2				0.7168
		Outer Marginal 1	Central 3				0.5403
		Outer Marginal 1	Inner Marginal 1				0.4795
		Outer Marginal 1	Inner Marginal 3				0.5403
		Outer Marginal 2	Central 3				0.5403
		Outer Marginal 2	Inner Marginal 1				0.4795
		Outer Marginal 2	Inner Marginal 3				0.5403
		Outer Marginal 3	Outer Marginal 1				0.5403
		Lateral 2	Inner Marginal 2				0.6528
		Central 2	Central 1				0.5784
		Outer Marginal 1	Lateral 2				0.5582
		Outer Marginal 1	Central 1				0.3458
		Outer Marginal 1	Inner Marginal 2				0.3711
		Outer Marginal 2	Central 1				0.3458
		Outer Marginal 2	Inner Marginal 2				0.3711
		Outer Marginal 3	Central 2				0.3404
		Outer Marginal 3	Central 3				0.2453
		Outer Marginal 3	Inner Marginal 1				0.2207
		Outer Marginal 3	Inner Marginal 3				0.2453
		Inner Marginal 3	Inner Marginal 2				0.2361
		Outer Marginal 3	Lateral 2				0.3329
		Outer Marginal 2	Lateral 2				0.3729
		Outer Marginal 2	Central 2				0.2765
		Outer Marginal 3	Central 1				0.1386
		Outer Marginal 3	Inner Marginal 2				0.1489
		Lateral 3	Inner Marginal 2				0.3041
		Lateral 3	Lateral 2				0.2676
	**P**	Lateral 3	Central 3	17.2038	7	0.0161*	0.0202*
		Lateral 3	Central 1				0.0917
		Lateral 3	Inner Marginal 1				0.2104
		Lateral 3	Inner Marginal 3				0.2777
		Lateral 3	Lateral 2				0.6558
		Lateral 2	Central 1				0.2030
		Lateral 2	Central 3				0.5613
		Inner Marginal 1	Central 3				0.4740
		Outer Marginal 1	Central 3				0.4750
		Lateral 2	Inner Marginal 1				0.3458
		Inner Marginal 3	Central 3				0.8379
		Inner Marginal 3	Inner Marginal 1				1.0000
		Lateral 2	Inner Marginal 3				1.0000
		Outer Marginal 1	Inner Marginal 1				1.0000
		Outer Marginal 1	Inner Marginal 3				1.0000
		Inner Marginal 3	Central 1				0.6961
		Inner Marginal 1	Central 1				0.5133
		Outer Marginal 1	Lateral 2				0.3711
		Outer Marginal 1	Central 1				0.3621
		Outer Marginal 3	Inner Marginal 3				0.2354
		Outer Marginal 3	Central 3				0.2296
		Central 3	Central 1				0.1743
		Outer Marginal 3	Lateral 2				0.2673
		Outer Marginal 3	Inner Marginal 1				0.1073
		Outer Marginal 3	Outer Marginal 1				0.1078
		Outer Marginal 3	Central 1				0.0167*
		Outer Marginal 1	Lateral 3				0.2104
		Outer Marginal 3	Lateral 3				0.0028*
	**S**	Outer Marginal 1	Lateral 3	31.8352	11	0.0008*	0.0393*
		Lateral 2	Central 3				0.0306*
		Inner Marginal 1	Central 3				0.0017*
		Outer Marginal 1	Central 3				0.0020*
		Lateral 3	Central 3				0.2001
		Lateral 1	Central 3				0.0024*
		Lateral 1	Inner Marginal 2				0.0521
		Lateral 2	Inner Marginal 2				0.1647
		Outer Marginal 1	Inner Marginal 2				0.0722
		Outer Marginal 1	Lateral 2				0.2421
		Outer Marginal 2	Central 3				0.1776
		Lateral 1	Inner Marginal 3				0.0646
		Inner Marginal 3	Central 3				0.0929
		Outer Marginal 2	Lateral 3				0.5251
		Lateral 3	Inner Marginal 2				0.5365
		Inner Marginal 3	Inner Marginal 2				0.3210
		Outer Marginal 1	Inner Marginal 3				0.3160
		Outer Marginal 2	Inner Marginal 2				0.4925
		Lateral 1	Inner Marginal 1				0.3738
		Lateral 1	Central 1				0.4518

		Central 2	Central 1				0.5440
		Lateral 2	Inner Marginal 3				0.8886
		Inner Marginal 1	Central 1				0.9149
		Lateral 1	Central 2				0.9626
		Inner Marginal 2	Central 3				1.0000
		Outer Marginal 1	Central 1				0.8310
		Outer Marginal 3	Central 3				0.7870
		Outer Marginal 2	Inner Marginal 3				0.7963
		Inner Marginal 1	Central 2				0.7487
		Outer Marginal 1	Inner Marginal 1				0.7228
		Outer Marginal 3	Inner Marginal 2				0.7574
		Outer Marginal 2	Lateral 2				0.7724
		Outer Marginal 1	Central 2				0.5222
		Inner Marginal 3	Central 1				0.3731
		Outer Marginal 1	Lateral 1				0.2616
		Outer Marginal 2	Central 1				0.3408
		Inner Marginal 3	Central 2				0.2232
		Lateral 2	Central 1				0.3856
		Inner Marginal 3	Inner Marginal 1				0.1389
		Lateral 3	Inner Marginal 3				0.5603
		Inner Marginal 2	Central 1				0.2165
		Outer Marginal 2	Outer Marginal 1				0.2230
		Outer Marginal 3	Inner Marginal 3				0.0750
		Outer Marginal 2	Inner Marginal 1				0.1124
		Outer Marginal 3	Outer Marginal 2				0.0944
		Lateral 2	Central 2				0.1659
		Outer Marginal 2	Lateral 1				0.0706
		Inner Marginal 2	Inner Marginal 1				0.0467*
		Outer Marginal 2	Central 2				0.0665
		Lateral 3	Lateral 2				0.2291
		Inner Marginal 2	Central 2				0.0420*
		Central 3	Central 1				0.0205*
		Central 3	Central 2				0.0184*
		Lateral 2	Inner Marginal 1				0.0926
		Outer Marginal 3	Central 1				0.0141*
		Outer Marginal 3	Central 2				0.0056*
		Outer Marginal 3	Lateral 3				0.1265
		Outer Marginal 3	Outer Marginal 1				0.0018*
		Outer Marginal 3	Lateral 1				0.0012*
		Lateral 2	Lateral 1				0.0472*
		Lateral 3	Central 1				0.0771
		Outer Marginal 3	Lateral 2				0.0168*
		Outer Marginal 3	Inner Marginal 1				0.0005*
		Lateral 3	Central 2				0.0246*
		Lateral 3	Inner Marginal 1				0.0137*
		Lateral 3	Lateral 1				0.0057*
		Lateral 3	Inner Marginal 1				0.0043*
		Lateral 3	Lateral 1				0.0043*
		Lateral 3	Inner Marginal 3				0.0020*
		Lateral 3	Lateral 2				0.0032*
		Lateral 3	Central 3				0.0154*
		Lateral 3	Central 2				0.2330
		Outer Marginal 3	Inner Marginal 1				0.0513
		Outer Marginal 3	Lateral 1				0.0513
		Lateral 2	Inner Marginal 1				0.0960
		Lateral 2	Lateral 1				0.0960
		Lateral 2	Inner Marginal 3				0.1252
		Outer Marginal 2	Inner Marginal 3				0.1752
		Outer Marginal 2	Lateral 2				0.4637
		Outer Marginal 3	Inner Marginal 3				0.3570
		Inner Marginal 2	Inner Marginal 1				0.1386
		Outer Marginal 2	Inner Marginal 1				0.1386
		Outer Marginal 2	Lateral 1				0.1386
		Lateral 1	Inner Marginal 1				0.3687
		Outer Marginal 2	Central 3				0.6953
		Inner Marginal 3	Inner Marginal 1				0.7642
		Inner Marginal 2	Central 2				1.0000
		Inner Marginal 2	Central 3				1.0000
	**Cl**	Outer Marginal 2	Central 2	38.2713	9	<.0001*	1.0000
		Outer Marginal 2	Inner Marginal 2				1.0000
		Lateral 1	Inner Marginal 3				0.7642
		Central 3	Central 2				0.6728
		Lateral 3	Inner Marginal 2				0.9142
		Lateral 2	Central 3				0.5887
		Outer Marginal 3	Lateral 2				0.5519
		Lateral 2	Inner Marginal 2				0.5205
		Lateral 1	Inner Marginal 2				0.1386
		Outer Marginal 3	Inner Marginal 2				0.4513
		Inner Marginal 3	Inner Marginal 2				0.2410
		Outer Marginal 3	Central 3				0.3061
		Lateral 2	Central 2				0.2309
		Outer Marginal 3	Outer Marginal 2				0.1958
		Outer Marginal 3	Central 2				0.1299
		Inner Marginal 3	Central 3				0.0401*
		Inner Marginal 3	Central 2				0.0277*
		Inner Marginal 1	Central 2				0.0184*
		Inner Marginal 1	Central 3				0.0186*
		Lateral 1	Central 2				0.0184*
		Lateral 1	Central 3				0.0186*
		Outer Marginal 2	Lateral 3				0.3456
		Outer Marginal 3	Lateral 3				0.0005*
		Central 3	Central 2				1.0000
	**K**	Outer Marginal 2	Central 2	5.0116	4	0.2861	1.0000
		Outer Marginal 2	Lateral 3				0.8432

		Lateral 2	Central 2				0.6374
		Outer Marginal 2	Lateral 2				0.6374
		Outer Marginal 2	Central 3				0.5403
		Lateral 2	Central 3				0.5536
		Lateral 3	Lateral 2				0.4678
		Lateral 3	Central 2				0.2393
		Lateral 3	Central 3				0.1460
	Ca	Lateral 3	Lateral 1	74.8328	11	<.0001*	0.0004*
		Lateral 3	Inner Marginal 1				0.0006*
		Lateral 3	Central 1				0.0018*
		Lateral 3	Inner Marginal 3				0.0107*
		Lateral 2	Lateral 1				0.0005*
		Lateral 2	Inner Marginal 1				0.0013*
		Lateral 2	Central 1				0.0027*
		Lateral 2	Inner Marginal 3				0.0096*
		Inner Marginal 2	Central 1				0.0003*
		Outer Marginal 2	Inner Marginal 3				0.0005*
		Outer Marginal 2	Lateral 1				0.0004*
		Outer Marginal 2	Inner Marginal 1				0.0004*
		Central 2	Central 1				0.0002*
		Inner Marginal 2	Inner Marginal 1				0.0005*
		Outer Marginal 2	Outer Marginal 1				0.0011*
		Outer Marginal 2	Central 1				0.0017*
		Outer Marginal 3	Lateral 1				0.0008*
		Outer Marginal 1	Lateral 1				0.0017*
		Central 3	Central 1				0.0150*
		Outer Marginal 3	Inner Marginal 1				0.0356*
		Inner Marginal 2	Central 3				0.1293
		Outer Marginal 1	Inner Marginal 1				0.1207
		Outer Marginal 3	Inner Marginal 3				0.2603
		Outer Marginal 3	Outer Marginal 1				0.3369
		Inner Marginal 3	Inner Marginal 1				0.3139
		Outer Marginal 3	Central 1				0.6231
		Inner Marginal 3	Central 1				0.7427
		Lateral 1	Inner Marginal 1				0.8583
		Lateral 3	Lateral 2				0.9104
		Outer Marginal 2	Lateral 2				0.8308
		Lateral 2	Central 3				0.7682
		Outer Marginal 2	Central 3				0.6458
		Inner Marginal 2	Central 2				0.6084
		Outer Marginal 1	Central 1				0.4550
		Outer Marginal 1	Inner Marginal 3				0.3763
		Lateral 3	Central 3				0.6942
		Outer Marginal 2	Lateral 3				0.5036
		Central 3	Central 2				0.0821
		Inner Marginal 3	Central 3				0.0332*
		Outer Marginal 3	Central 3				0.0490*
		Inner Marginal 1	Central 1				0.0500*
		Lateral 1	Inner Marginal 3				0.0047*
		Outer Marginal 1	Central 3				0.0107*
		Inner Marginal 1	Central 3				0.0079*
		Lateral 1	Central 3				0.0023*
		Inner Marginal 3	Central 2				0.0043*
		Lateral 2	Inner Marginal 2				0.0369*
		Lateral 1	Central 1				0.0016*
		Inner Marginal 3	Inner Marginal 2				0.0031*
		Lateral 1	Central 2				0.0009*
		Outer Marginal 3	Lateral 2				0.0216*
		Outer Marginal 2	Inner Marginal 2				0.0047*
		Outer Marginal 1	Central 2				0.0006*
		Outer Marginal 2	Central 2				0.0024*
		Inner Marginal 1	Central 2				0.0004*
		Outer Marginal 3	Outer Marginal 2				0.0017*
		Outer Marginal 3	Inner Marginal 2				0.0013*
		Outer Marginal 3	Central 2				0.0006*
		Lateral 2	Central 2				0.0093*
		Lateral 1	Inner Marginal 2				0.0005*
		Outer Marginal 1	Inner Marginal 2				0.0004*
		Outer Marginal 1	Lateral 2				0.0024*
		Lateral 3	Inner Marginal 2				0.0084*
		Outer Marginal 3	Lateral 3				0.0060*
		Lateral 3	Central 2				0.0022*
		Outer Marginal 1	Lateral 3				0.0013*
	Fe	Lateral 3	Lateral 2	3.4404	2	0.1790	0.1161
		Lateral 2	Central 2				0.6973
		Lateral 3	Central 2				0.5403
	All elements	Lateral 3	Inner Marginal 3	88.5554	11	<.0001*	<.0001*
		Lateral 3	Lateral 1				<.0001*
		Lateral 3	Lateral 2				0.0002*
		Lateral 3	Inner Marginal 1				0.0007*
		Lateral 3	Inner Marginal 2				0.0003*
		Lateral 3	Central 1				0.0008*
		Lateral 2	Lateral 1				0.0002*
		Lateral 2	Inner Marginal 3				0.0002*
		Outer Marginal 2	Lateral 1				0.0005*
		Outer Marginal 3	Lateral 1				0.0009*
		Lateral 2	Inner Marginal 1				0.0341*
		Central 2	Central 1				0.0051*
		Outer Marginal 2	Inner Marginal 3				0.0131*
		Outer Marginal 1	Lateral 1				0.0017*
		Central 3	Central 1				0.0077*
		Outer Marginal 3	Inner Marginal 3				0.0078*
		Outer Marginal 1	Inner Marginal 3				0.0237*
		Inner Marginal 2	Inner Marginal 1				0.1065

		Outer Marginal 2	Inner Marginal 1				0.1653
		Outer Marginal 3	Inner Marginal 1				0.1883
		Outer Marginal 1	Inner Marginal 1				0.1995
		Lateral 2	Inner Marginal 2				0.4975
		Lateral 2	Central 1				0.5032
		Outer Marginal 3	Outer Marginal 2				0.4436
		Central 3	Central 2				0.6160
		Inner Marginal 2	Central 1				0.8709
		Inner Marginal 3	Inner Marginal 1				0.8851
		Outer Marginal 3	Outer Marginal 1				0.9716
		Lateral 3	Central 2				0.8574
		Lateral 1	Inner Marginal 1				0.6789
		Outer Marginal 2	Outer Marginal 1				0.5521
		Outer Marginal 3	Central 1				0.4703
		Lateral 3	Central 3				0.7131
		Lateral 1	Inner Marginal 3				0.2714
		Outer Marginal 1	Central 1				0.3023
		Outer Marginal 3	Inner Marginal 2				0.3413
		Outer Marginal 1	Inner Marginal 2				0.3217
		Inner Marginal 1	Central 1				0.0945
		Outer Marginal 2	Central 1				0.0857
		Outer Marginal 3	Lateral 2				0.1662
		Inner Marginal 1	Central 3				0.0081*
		Outer Marginal 1	Central 3				0.0081*
		Outer Marginal 1	Lateral 2				0.1040
		Lateral 1	Central 3				0.0024*
		Inner Marginal 2	Central 3				0.0184*
		Inner Marginal 3	Central 1				0.0061*
		Outer Marginal 3	Central 3				0.0055*
		Inner Marginal 3	Central 3				0.0014*
		Outer Marginal 1	Central 2				0.0050*
		Inner Marginal 1	Central 2				0.0025*
		Outer Marginal 2	Inner Marginal 2				0.0098*
		Outer Marginal 3	Central 2				0.0024*
		Inner Marginal 2	Central 2				0.0050*
		Lateral 1	Central 1				0.0009*
		Lateral 1	Central 2				0.0009*
		Inner Marginal 3	Central 2				0.0008*
		Lateral 2	Central 2				0.0131*
		Inner Marginal 3	Inner Marginal 2				0.0008*
		Lateral 2	Central 3				0.0113*
		Outer Marginal 2	Central 3				0.0004*
		Lateral 1	Inner Marginal 2				0.0005*
		Outer Marginal 2	Central 2				0.0003*
		Outer Marginal 2	Lateral 2				0.0018*
		Outer Marginal 1	Lateral 3				0.0002*
		Outer Marginal 3	Lateral 3				<.0001*
		Outer Marginal 2	Lateral 3				<.0001*
		Lateral 3	Inner Marginal 3				0.0196*
		Lateral 3	Central 1				0.0653
		Lateral 2	Inner Marginal 3				0.0293*
		Lateral 3	Lateral 2				0.3315
		Lateral 2	Central 1				0.1721
		Central 3	Central 1				0.0604
		Outer Marginal 1	Inner Marginal 3				0.0635
		Inner Marginal 2	Central 1				0.1720
		Central 2	Central 1				0.3990
		Outer Marginal 3	Inner Marginal 3				0.4142
		Inner Marginal 2	Central 3				1.0000
		Central 3	Central 2				0.8948
		Inner Marginal 2	Central 2				0.7374
		Inner Marginal 3	Central 2				0.2453
		Outer Marginal 1	Central 1				0.4680
		Outer Marginal 3	Central 2				0.2453
		Outer Marginal 1	Central 2				0.3990
	**Cu**	Lateral 3	Inner Marginal 2	20.1536	8	0.0098*	0.6477
		Outer Marginal 1	Inner Marginal 2				0.1720
		Lateral 2	Central 2				0.4392
		Lateral 2	Inner Marginal 2				0.3193
		Inner Marginal 3	Central 1				0.0635
		Inner Marginal 3	Inner Marginal 2				0.0668
		Outer Marginal 3	Central 1				0.0635
		Outer Marginal 3	Inner Marginal 2				0.0668
		Outer Marginal 3	Outer Marginal 1				0.0635
		Lateral 2	Central 3				0.1875
		Lateral 3	Central 2				0.6364
		Outer Marginal 1	Central 3				0.0604
		Lateral 3	Central 3				0.4063
		Inner Marginal 3	Central 3				0.0495*
		Outer Marginal 3	Central 3				0.0495*
		Outer Marginal 1	Lateral 2				0.1303
		Outer Marginal 3	Lateral 2				0.0293*
		Outer Marginal 1	Lateral 3				0.0484*
		Outer Marginal 3	Lateral 3				0.0196*
		Outer Marginal 3	Inner Marginal 2				1.0000
	**F**	Outer Marginal 3	Outer Marginal 1	2.2500	2	0.3247	1.0000
		Outer Marginal 1	Inner Marginal 2				0.5403
		Lateral II 2	Lateral I 4				0.0033*
Vittina turrita		Outer Marginal 3	Outer Marginal 1				0.0026*
		Outer Marginal 1	Lateral I 4				0.0033*
	**Na**	Outer Marginal 3	Lateral I 4	60.4272	15	<.0001*	0.0033*
		Outer Marginal 3	Lateral II 4				0.0034*
		Outer Marginal 3	Lateral I 3				0.0049*
		Lateral II 2	Inner Marginal 4				0.0137*

		Lateral II 2	Lateral I 1				0.0141*
		Lateral II 3	Lateral I 3				0.0058*
		Outer Marginal 3	Lateral I 2				0.0058*
		Lateral II 4	Lateral I 4				0.0061*
		Outer Marginal 1	Lateral II 4				0.0119*
		Lateral II 3	Lateral I 4				0.0078*
		Lateral II 3	Lateral I 2				0.0122*
		Lateral I 3	Inner Marginal 4				0.0210*
		Lateral I 3	Lateral I 1				0.0222*
		Outer Marginal 1	Inner Marginal 4				0.0210*
		Outer Marginal 1	Lateral I 1				0.0222*
		Outer Marginal 3	Inner Marginal 4				0.0210*
		Outer Marginal 3	Lateral I 1				0.0222*
		Lateral II 2	Lateral II 1				0.1059
		Lateral II 2	Inner Marginal 1				0.2049
		Lateral II 4	Inner Marginal 4				0.0250*
		Lateral II 4	Lateral I 1				0.0269*
		Outer Marginal 1	Lateral II 1				0.0570
		Outer Marginal 3	Lateral II 1				0.0570
		Lateral II 2	Lateral I 2				0.0982
		Lateral II 2	Lateral I 3				0.1073
		Lateral I 2	Inner Marginal 4				0.0325*
		Lateral I 2	Lateral I 1				0.0358*
		Lateral II 3	Inner Marginal 4				0.0325*
		Lateral II 3	Lateral I 1				0.0358*
		Lateral II 3	Lateral II 2				0.1583
		Lateral I 3	Inner Marginal 1				0.1904
		Outer Marginal 1	Inner Marginal 1				0.1904
		Outer Marginal 2	Lateral I 3				0.1904
		Outer Marginal 2	Outer Marginal 1				0.1904
		Outer Marginal 3	Inner Marginal 1				0.1904
		Lateral II 4	Lateral II 1				0.0916
		Lateral II 3	Lateral II 1				0.0814
		Lateral II 4	Inner Marginal 1				0.2072
		Outer Marginal 2	Lateral I 4				0.2030
		Outer Marginal 2	Lateral II 4				0.2072
		Lateral I 3	Lateral I 2				0.1667
		Lateral I 4	Inner Marginal 4				0.1179
		Lateral II 3	Inner Marginal 2				0.1360
		Lateral II 3	Inner Marginal 3				0.1771
		Lateral I 2	Inner Marginal 1				0.2416
		Lateral II 3	Inner Marginal 1				0.2416
		Outer Marginal 2	Lateral I 2				0.2416
		Outer Marginal 3	Inner Marginal 2				0.3036
		Outer Marginal 4	Inner Marginal 4				0.1685
		Lateral II 1	Inner Marginal 4				0.1066
		Lateral II 1	Lateral I 1				0.1386
		Lateral II 1	Lateral I 4				0.2994
		Lateral I 1	Inner Marginal 4				0.1876
		Outer Marginal 3	Inner Marginal 3				0.4941
		Inner Marginal 2	Inner Marginal 1				0.3711
		Inner Marginal 3	Inner Marginal 1				0.3711
		Outer Marginal 2	Inner Marginal 2				0.3711
		Outer Marginal 2	Inner Marginal 4				0.2482
		Outer Marginal 2	Lateral I 1				0.3458
		Lateral I 4	Lateral I 1				0.5021
		Outer Marginal 2	Lateral II 1				0.5403
		Outer Marginal 2	Inner Marginal 3				0.6374
		Outer Marginal 3	Lateral II 2				0.8070
		Outer Marginal 2	Lateral II 2				0.8744
		Lateral II 2	Inner Marginal 2				0.8656
		Lateral II 2	Inner Marginal 3				0.8656
		Inner Marginal 3	Inner Marginal 2				1.0000
		Lateral II 1	Inner Marginal 1				1.0000
		Outer Marginal 2	Inner Marginal 1				1.0000
		Outer Marginal 2	Lateral II 3				1.0000
		Outer Marginal 3	Outer Marginal 2				1.0000
		Outer Marginal 4	Lateral I 1				1.0000
		Lateral I 4	Inner Marginal 1				0.7933
		Outer Marginal 4	Lateral I 4				0.5049
		Inner Marginal 4	Inner Marginal 1				0.2482
		Lateral I 1	Inner Marginal 1				0.3458
		Outer Marginal 1	Lateral I 2				0.4641
		Outer Marginal 4	Inner Marginal 1				0.3583
		Lateral II 1	Inner Marginal 2				0.1489
		Lateral II 1	Inner Marginal 3				0.1489
		Lateral I 3	Inner Marginal 3				0.2545
		Outer Marginal 4	Outer Marginal 2				0.2278
		Inner Marginal 4	Inner Marginal 2				0.0636
		Inner Marginal 4	Inner Marginal 3				0.0636
		Lateral I 1	Inner Marginal 2				0.0765
		Lateral I 1	Inner Marginal 3				0.0765
		Outer Marginal 4	Lateral II 1				0.1113
		Outer Marginal 3	Lateral II 3				0.1667
		Lateral II 1	Lateral I 2				0.0814
		Lateral I 2	Inner Marginal 3				0.0736
		Outer Marginal 1	Lateral I 3				0.1413
		Lateral I 2	Inner Marginal 2				0.0369*
		Outer Marginal 4	Inner Marginal 2				0.0347*
		Outer Marginal 4	Inner Marginal 3				0.0347*
		Lateral I 3	Inner Marginal 2				0.0682
		Outer Marginal 1	Inner Marginal 3				0.0682
		Lateral II 1	Lateral I 3				0.0570
		Lateral I 4	Inner Marginal 2				0.0269*

		Lateral I 4	Inner Marginal 3				0.0269*
		Lateral II 4	Inner Marginal 2				0.0275*
		Lateral II 4	Inner Marginal 3				0.0275*
		Outer Marginal 1	Lateral II 2				0.0788
		Outer Marginal 1	Inner Marginal 2				0.0299*
		Lateral II 4	Lateral II 2				0.0506
		Outer Marginal 4	Lateral I 2				0.0117*
		Outer Marginal 4	Lateral II 3				0.0117*
		Lateral I 4	Lateral I 2				0.0078*
		Lateral II 4	Lateral I 2				0.0080*
		Lateral II 4	Lateral II 3				0.0080*
		Outer Marginal 4	Lateral II 4				0.0077*
		Outer Marginal 1	Lateral II 3				0.0058*
		Outer Marginal 4	Lateral I 3				0.0056*
		Outer Marginal 4	Outer Marginal 1				0.0056*
		Outer Marginal 4	Outer Marginal 3				0.0056*
		Lateral I 4	Lateral I 3				0.0033*
		Lateral II 4	Lateral I 3				0.0034*
		Outer Marginal 4	Lateral II 2				0.0032*
		Lateral II 4	Lateral I 4				0.0011*
		Outer Marginal 3	Lateral II 4				0.0029*
		Lateral II 4	Inner Marginal 1				0.0294*
		Lateral II 4	Inner Marginal 4				0.0294*
		Lateral II 4	Lateral II 1				0.0294*
		Lateral II 2	Lateral I 4				0.0047*
		Lateral II 4	Lateral I 1				0.1258
		Lateral II 2	Inner Marginal 4				0.0465*
		Lateral II 2	Lateral II 1				0.0566
		Lateral II 2	Inner Marginal 1				0.0679
		Outer Marginal 2	Lateral II 4				0.0654
		Lateral II 2	Lateral I 1				0.2030
		Lateral II 2	Inner Marginal 3				0.1315
		Lateral II 2	Lateral I 2				0.1315
		Lateral II 2	Lateral I 3				0.0955
		Lateral II 3	Lateral I 4				0.0195*
		Outer Marginal 2	Lateral I 4				0.0195*
		Outer Marginal 3	Lateral I 3				0.0200*
		Outer Marginal 3	Lateral I 4				0.0195*
		Lateral II 3	Lateral I 3				0.0373*
		Outer Marginal 2	Outer Marginal 1				0.0304*
		Outer Marginal 3	Outer Marginal 1				0.0304*
		Lateral II 2	Inner Marginal 2				0.3020
		Lateral I 3	Inner Marginal 1				0.0814
		Lateral I 3	Inner Marginal 4				0.0786
		Lateral II 3	Inner Marginal 1				0.1052
		Lateral II 3	Inner Marginal 2				0.1052
		Lateral II 3	Inner Marginal 3				0.1052
		Lateral II 3	Inner Marginal 4				0.1002
		Lateral II 3	Lateral I 2				0.1052
		Lateral II 3	Lateral II 1				0.1052
		Outer Marginal 2	Inner Marginal 1				0.1052
		Outer Marginal 2	Inner Marginal 4				0.1002
		Outer Marginal 2	Lateral II 1				0.1052
		Outer Marginal 3	Inner Marginal 1				0.1052
		Outer Marginal 3	Inner Marginal 2				0.1052
		Outer Marginal 3	Inner Marginal 3				0.1052
	**Mg**	Outer Marginal 3	Inner Marginal 4	53.5764	15	<.0001*	0.1002
		Outer Marginal 3	Lateral I 2				0.1052
		Outer Marginal 3	Lateral II 1				0.1052
		Lateral I 3	Lateral I 1				0.2416
		Lateral II 3	Lateral II 2				0.5238
		Lateral II 3	Lateral I 1				0.2888
		Outer Marginal 1	Inner Marginal 4				0.2188
		Outer Marginal 2	Lateral I 1				0.2888
		Outer Marginal 3	Lateral I 1				0.2888
		Lateral I 4	Inner Marginal 4				0.2854
		Inner Marginal 2	Inner Marginal 1				0.2453
		Lateral I 2	Inner Marginal 1				0.2453
		Lateral I 2	Inner Marginal 4				0.2207
		Outer Marginal 3	Outer Marginal 2				0.3836
		Outer Marginal 1	Lateral II 1				0.4745
		Outer Marginal 2	Inner Marginal 3				0.4875
		Outer Marginal 2	Lateral I 2				0.4875
		Outer Marginal 2	Lateral I 3				0.5403
		Outer Marginal 4	Inner Marginal 4				0.4113
		Lateral I 3	Inner Marginal 3				0.5613
		Lateral I 3	Lateral I 2				0.5613
		Outer Marginal 3	Lateral II 2				0.7499
		Lateral I 1	Inner Marginal 1				0.5403
		Lateral I 1	Inner Marginal 4				0.4795
		Lateral I 2	Lateral I 1				0.5403
		Lateral II 1	Inner Marginal 4				0.6171
		Lateral II 4	Inner Marginal 3				0.9440
		Outer Marginal 1	Lateral I 4				0.9000
		Inner Marginal 3	Inner Marginal 1				1.0000
		Lateral I 1	Inner Marginal 3				1.0000
		Lateral I 2	Inner Marginal 3				1.0000
		Lateral I 4	Lateral I 1				1.0000
		Lateral II 4	Lateral I 2				1.0000
		Outer Marginal 1	Inner Marginal 1				1.0000
		Outer Marginal 1	Lateral I 1				1.0000
		Outer Marginal 2	Inner Marginal 2				1.0000
		Outer Marginal 4	Lateral II 1				1.0000
		Outer Marginal 3	Lateral II 3				0.8852

		Lateral I 4	Inner Marginal 1				0.8451
		Lateral II 1	Lateral I 4				0.6849
		Lateral I 1	Inner Marginal 2				0.5403
		Lateral II 1	Lateral I 1				0.5403
		Outer Marginal 4	Lateral I 4				0.6072
		Inner Marginal 3	Inner Marginal 2				0.4142
		Lateral I 3	Inner Marginal 2				0.5613
		Outer Marginal 1	Inner Marginal 3				0.4875
		Inner Marginal 4	Inner Marginal 1				0.2207
		Inner Marginal 4	Inner Marginal 2				0.2207
		Inner Marginal 4	Inner Marginal 3				0.2207
		Lateral I 2	Inner Marginal 2				0.2453
		Lateral II 1	Inner Marginal 1				0.2453
		Lateral II 1	Inner Marginal 2				0.2453
		Lateral II 1	Inner Marginal 3				0.2453
		Lateral II 1	Lateral I 2				0.2453
		Outer Marginal 4	Outer Marginal 1				0.3719
		Outer Marginal 2	Lateral II 2				0.5952
		Lateral I 4	Inner Marginal 3				0.3286
		Outer Marginal 2	Lateral II 3				0.3123
		Outer Marginal 4	Inner Marginal 3				0.2403
		Outer Marginal 4	Lateral I 1				0.2765
		Outer Marginal 4	Inner Marginal 1				0.1527
		Outer Marginal 1	Inner Marginal 2				0.1052
		Outer Marginal 1	Lateral I 2				0.1052
		Outer Marginal 4	Inner Marginal 2				0.1002
		Outer Marginal 4	Lateral I 2				0.1002
		Lateral I 4	Inner Marginal 2				0.0786
		Lateral I 4	Lateral I 2				0.0786
		Lateral II 1	Lateral I 3				0.0814
		Outer Marginal 1	Lateral II 3				0.0304*
		Outer Marginal 4	Lateral II 3				0.0294*
		Outer Marginal 4	Outer Marginal 2				0.0294*
		Outer Marginal 4	Outer Marginal 3				0.0294*
		Outer Marginal 1	Lateral I 3				0.0200*
		Outer Marginal 4	Lateral I 3				0.0195*
		Lateral I 4	Lateral I 3				0.0119*
		Lateral II 4	Lateral I 3				0.0985
		Outer Marginal 1	Lateral II 2				0.0108*
		Outer Marginal 4	Lateral II 2				0.0091*
		Lateral II 4	Inner Marginal 2				0.0416*
		Lateral II 4	Lateral II 3				0.0029*
		Outer Marginal 1	Lateral II 4				0.0029*
		Outer Marginal 4	Lateral II 4				0.0029*
		Lateral II 4	Lateral II 2				0.0008*
		Lateral II 4	Lateral I 4				<.0001*
		Lateral I 4	Inner Marginal 4				<.0001*
		Lateral II 4	Inner Marginal 4				<.0001*
		Lateral II 2	Inner Marginal 4				<.0001*
		Lateral I 4	Lateral I 3				0.0030*
		Outer Marginal 2	Lateral I 4				0.0254*
		Outer Marginal 3	Lateral I 4				0.0254*
		Lateral II 2	Lateral I 4				0.0025*
		Lateral II 4	Lateral I 3				0.0011*
		Lateral II 3	Lateral I 4				0.1151
		Lateral II 4	Inner Marginal 2				0.0013*
		Lateral II 4	Inner Marginal 1				0.0294*
		Lateral II 4	Lateral I 1				0.0294*
		Lateral II 4	Lateral I 2				0.0294*
		Lateral II 4	Lateral II 1				0.0294*
		Lateral II 2	Lateral I 3				0.0022*
		Lateral II 4	Lateral II 2				0.0103*
		Lateral I 4	Inner Marginal 1				0.1217
		Lateral I 4	Lateral I 1				0.1217
		Lateral I 4	Lateral I 2				0.1217
		Outer Marginal 1	Inner Marginal 4				0.0352*
		Outer Marginal 2	Inner Marginal 4				0.0352*
		Outer Marginal 3	Inner Marginal 4				0.0352*
		Lateral II 2	Inner Marginal 1				0.0502
	**Si**	Lateral II 2	Lateral I 1	79.5076	15	<.0001*	0.0502
		Lateral II 2	Lateral I 2				0.0502
		Lateral II 2	Lateral II 1				0.0502
		Outer Marginal 1	Lateral I 4				0.1685
		Lateral II 3	Lateral II 2				0.1353
		Outer Marginal 3	Lateral II 2				0.0738
		Lateral II 3	Inner Marginal 4				0.1399
		Lateral II 2	Inner Marginal 2				0.0801
		Outer Marginal 2	Inner Marginal 2				0.0460*
		Outer Marginal 3	Inner Marginal 2				0.0460*
		Inner Marginal 2	Inner Marginal 1				0.0838
		Inner Marginal 3	Inner Marginal 2				0.1643
		Lateral II 3	Inner Marginal 2				0.1643
		Lateral II 4	Inner Marginal 3				0.4747
		Outer Marginal 1	Lateral I 3				0.0786
		Outer Marginal 2	Lateral I 3				0.0786
		Outer Marginal 3	Lateral I 3				0.0786
		Outer Marginal 4	Lateral I 3				0.3393
		Outer Marginal 3	Lateral II 4				0.5270
		Outer Marginal 4	Inner Marginal 4				0.4271
		Lateral II 3	Lateral I 3				0.2348
		Outer Marginal 2	Lateral II 2				0.4954
		Outer Marginal 1	Inner Marginal 2				0.3519
		Outer Marginal 1	Inner Marginal 1				0.2453
		Outer Marginal 1	Lateral I 1				0.2453

		Outer Marginal 1	Lateral I 2				0.2453
		Outer Marginal 1	Lateral II 1				0.2453
		Outer Marginal 2	Inner Marginal 1				0.2453
		Outer Marginal 2	Lateral I 1				0.2453
		Outer Marginal 2	Lateral I 2				0.2453
		Outer Marginal 2	Lateral II 1				0.2453
		Outer Marginal 2	Outer Marginal 1				0.2453
		Outer Marginal 3	Inner Marginal 1				0.2453
		Outer Marginal 3	Lateral I 1				0.2453
		Outer Marginal 3	Lateral I 2				0.2453
		Outer Marginal 3	Lateral II 1				0.2453
		Outer Marginal 3	Outer Marginal 1				0.2453
		Outer Marginal 3	Outer Marginal 2				0.2453
		Lateral I 1	Inner Marginal 4				0.6459
		Lateral I 2	Inner Marginal 4				0.6459
		Inner Marginal 3	Inner Marginal 1				0.5403
		Lateral II 3	Inner Marginal 1				0.5403
		Lateral II 3	Lateral I 1				0.5403
		Lateral II 3	Lateral I 2				0.5403
		Lateral II 3	Lateral II 1				0.5403
		Outer Marginal 3	Inner Marginal 3				0.5403
		Lateral II 1	Lateral I 3				0.6933
		Lateral II 1	Inner Marginal 4				0.8545
		Lateral I 1	Inner Marginal 1				1.0000
		Lateral I 2	Inner Marginal 1				1.0000
		Lateral I 2	Lateral I 1				1.0000
		Lateral II 1	Inner Marginal 1				1.0000
		Lateral II 1	Lateral I 1				1.0000
		Lateral II 1	Lateral I 2				1.0000
		Lateral II 3	Inner Marginal 3				1.0000
		Outer Marginal 2	Inner Marginal 3				1.0000
		Outer Marginal 3	Lateral II 3				1.0000
		Lateral I 1	Inner Marginal 3				0.5403
		Lateral I 2	Inner Marginal 3				0.5403
		Lateral II 1	Inner Marginal 3				0.5403
		Outer Marginal 1	Inner Marginal 3				0.5403
		Outer Marginal 1	Lateral II 3				0.5403
		Outer Marginal 2	Lateral II 3				0.5403
		Outer Marginal 4	Lateral II 1				0.8321
		Lateral I 3	Lateral I 1				0.4170
		Lateral I 3	Lateral I 2				0.4170
		Inner Marginal 4	Inner Marginal 1				0.6459
		Outer Marginal 4	Inner Marginal 1				0.6718
		Outer Marginal 4	Lateral I 1				0.6718
		Outer Marginal 4	Lateral I 2				0.6718
		Lateral I 3	Inner Marginal 1				0.3150
		Lateral II 2	Inner Marginal 3				0.6186
		Lateral I 3	Inner Marginal 3				0.2348
		Outer Marginal 1	Lateral II 2				0.4435
		Lateral I 3	Inner Marginal 4				0.3125
		Lateral I 4	Inner Marginal 2				0.2730
		Lateral I 1	Inner Marginal 2				0.0838
		Lateral I 2	Inner Marginal 2				0.0838
		Lateral II 1	Inner Marginal 2				0.0838
		Outer Marginal 2	Lateral II 4				0.2918
		Inner Marginal 4	Inner Marginal 3				0.1399
		Lateral I 3	Inner Marginal 2				0.0062*
		Outer Marginal 1	Lateral II 4				0.1219
		Lateral II 1	Lateral I 4				0.1217
		Outer Marginal 4	Outer Marginal 1				0.0567
		Lateral II 4	Lateral II 3				0.1256
		Outer Marginal 4	Inner Marginal 3				0.1237
		Outer Marginal 4	Lateral II 3				0.1237
		Outer Marginal 4	Outer Marginal 2				0.0287*
		Outer Marginal 4	Outer Marginal 3				0.0287*
		Inner Marginal 4	Inner Marginal 2				0.0002*
		Lateral I 4	Inner Marginal 3				0.1151
		Outer Marginal 4	Inner Marginal 2				0.0007*
		Outer Marginal 4	Lateral I 4				0.0004*
		Outer Marginal 4	Lateral II 2				<.0001*
		Outer Marginal 4	Lateral II 4				<.0001*
		Lateral II 2	Lateral I 4				<.0001*
		Lateral II 2	Lateral I 3				0.0007*
		Lateral II 2	Inner Marginal 4				0.0010*
		Lateral II 3	Lateral II 2				0.0024*
		Lateral II 2	Lateral I 2				0.0035*
		Lateral II 2	Lateral II 1				0.0342*
		Lateral II 2	Inner Marginal 1				0.0385*
		Lateral II 2	Lateral I 1				0.0186*
		Lateral II 3	Lateral I 4				0.0009*
		Lateral II 2	Inner Marginal 3				0.1875
		Lateral I 4	Inner Marginal 1				0.0271*
	P	Lateral II 4	Lateral I 3	62.0254	11	<.0001*	0.0024*
		Lateral II 4	Inner Marginal 1				0.0294*
		Lateral II 4	Lateral II 1				0.0294*
		Lateral II 4	Lateral I 1				0.0162*
		Lateral II 4	Lateral I 2				0.0093*
		Lateral I 4	Lateral I 3				0.0118*
		Lateral I 4	Lateral I 1				0.0344*
		Lateral II 4	Lateral I 4				0.0207*
		Lateral II 4	Inner Marginal 4				0.0177*
		Outer Marginal 4	Lateral II 1				0.0412*
		Outer Marginal 4	Inner Marginal 1				0.0613
		Lateral I 4	Lateral I 2				0.0731

		Lateral II 3	Inner Marginal 4				0.0058*
		Lateral II 3	Lateral I 3				0.0056*
		Lateral II 3	Lateral I 2				0.0122*
		Outer Marginal 4	Lateral I 1				0.1213
		Outer Marginal 4	Lateral I 3				0.1122
		Inner Marginal 4	Inner Marginal 1				0.0570
		Lateral I 3	Inner Marginal 1				0.0550
		Lateral I 4	Inner Marginal 4				0.2252
		Lateral II 3	Lateral I 1				0.0369*
		Lateral II 4	Inner Marginal 3				0.4750
		Lateral I 2	Inner Marginal 1				0.0814
		Lateral II 3	Inner Marginal 1				0.0814
		Lateral II 3	Lateral II 1				0.0814
		Outer Marginal 4	Lateral I 2				0.3485
		Lateral II 3	Inner Marginal 3				0.2416
		Lateral I 1	Inner Marginal 1				0.1489
		Outer Marginal 4	Inner Marginal 4				0.7811
		Inner Marginal 3	Inner Marginal 1				0.5403
		Lateral I 2	Lateral I 1				0.8793
		Lateral I 3	Lateral I 1				0.9067
		Lateral I 4	Inner Marginal 3				1.0000
		Outer Marginal 4	Inner Marginal 3				1.0000
		Lateral I 3	Lateral I 2				0.8687
		Lateral II 1	Inner Marginal 3				0.5403
		Lateral II 1	Inner Marginal 1				0.4142
		Inner Marginal 4	Inner Marginal 3				0.6625
		Lateral I 2	Inner Marginal 3				0.5582
		Lateral I 1	Inner Marginal 3				0.3711
		Lateral II 1	Lateral I 1				0.1489
		Lateral I 2	Inner Marginal 4				0.2903
		Lateral I 3	Inner Marginal 3				0.3770
		Lateral I 1	Inner Marginal 4				0.1715
		Lateral II 1	Lateral I 2				0.0814
		Lateral I 3	Inner Marginal 4				0.1404
		Lateral II 1	Inner Marginal 4				0.0570
		Lateral II 1	Lateral I 3				0.0550
		Outer Marginal 4	Lateral I 4				0.1855
		Outer Marginal 4	Lateral II 4				0.0117*
		Lateral II 4	Lateral II 2				0.0282*
		Outer Marginal 4	Lateral II 3				0.0016*
		Lateral II 1	Lateral I 4				0.0271*
		Lateral II 4	Lateral II 3				0.0011*
		Outer Marginal 4	Lateral II 2				<.0001*
		Lateral II 2	Lateral I 4				<.0001*
		Outer Marginal 2	Lateral I 4				0.0002*
		Lateral II 3	Lateral I 4				0.0008*
		Lateral II 2	Lateral I 3				0.0031*
		Outer Marginal 3	Lateral I 4				0.0010*
		Outer Marginal 2	Lateral II 4				0.0002*
		Lateral II 2	Lateral I 2				0.0066*
		Lateral II 2	Inner Marginal 4				0.0024*
		Outer Marginal 4	Outer Marginal 1				0.0002*
		Lateral II 4	Lateral I 4				0.0014*
		Lateral II 2	Lateral I 1				0.0102*
		Lateral II 2	Lateral II 1				0.0120*
		Lateral II 3	Lateral II 2				0.0165*
		Lateral II 2	Inner Marginal 1				0.0262*
		Outer Marginal 2	Inner Marginal 4				0.0006*
		Lateral II 3	Inner Marginal 4				0.0019*
		Outer Marginal 3	Lateral II 4				0.0083*
		Outer Marginal 3	Inner Marginal 4				0.0078*
		Outer Marginal 4	Lateral I 4				0.0465*
		Outer Marginal 2	Outer Marginal 1				0.0022*
		Outer Marginal 3	Outer Marginal 1				0.0022*
		Lateral I 4	Lateral I 2				0.0891
		Lateral II 4	Lateral I 2				0.0523
		Outer Marginal 4	Lateral I 2				0.0575
		Lateral II 3	Lateral I 3				0.0057*
	S	Outer Marginal 2	Lateral I 1	88.3610	15	<.0001*	0.0058*
		Outer Marginal 2	Lateral II 1				0.0058*
		Outer Marginal 2	Lateral I 3				0.0105*
		Outer Marginal 3	Lateral I 3				0.0105*
		Inner Marginal 2	Inner Marginal 1				0.0157*
		Outer Marginal 2	Inner Marginal 1				0.0149*
		Outer Marginal 2	Lateral I 2				0.0149*
		Outer Marginal 3	Lateral I 2				0.0149*
		Lateral II 4	Lateral I 1				0.1266
		Lateral II 4	Lateral I 3				0.1163
		Outer Marginal 4	Lateral I 3				0.1160
		Lateral II 3	Inner Marginal 1				0.0122*
		Lateral II 3	Lateral I 1				0.0122*
		Lateral II 3	Lateral I 2				0.0122*
		Lateral II 3	Lateral II 1				0.0122*
		Outer Marginal 3	Lateral I 1				0.0230*
		Outer Marginal 3	Lateral II 1				0.0230*
		Lateral II 4	Lateral II 1				0.1371
		Outer Marginal 3	Inner Marginal 1				0.0513
		Lateral I 4	Lateral I 3				0.2444
		Inner Marginal 3	Inner Marginal 1				0.0947
		Outer Marginal 4	Lateral I 1				0.3216
		Outer Marginal 4	Lateral II 1				0.3636
		Lateral II 3	Inner Marginal 3				0.2101
		Outer Marginal 3	Lateral II 2				0.6198
		Lateral II 3	Inner Marginal 2				0.4208

		Inner Marginal 3	Inner Marginal 2				0.5101
		Lateral II 1	Lateral I 2				0.4633
		Lateral I 3	Lateral I 2				0.7418
		Lateral II 1	Lateral I 3				0.7436
		Outer Marginal 3	Outer Marginal 2				0.7983
		Lateral II 1	Lateral I 1				0.9161
		Lateral I 4	Lateral I 1				1.0000
		Lateral II 1	Lateral I 4				1.0000
		Lateral I 3	Lateral I 1				0.8059
		Inner Marginal 4	Inner Marginal 1				0.8329
		Outer Marginal 2	Lateral II 2				0.7767
		Lateral I 2	Lateral I 1				0.5284
		Lateral I 3	Inner Marginal 1				0.5684
		Outer Marginal 2	Inner Marginal 3				0.5160
		Outer Marginal 3	Inner Marginal 3				0.5160
		Lateral I 2	Inner Marginal 1				0.4633
		Outer Marginal 3	Inner Marginal 2				0.4871
		Outer Marginal 2	Inner Marginal 2				0.4519
		Lateral II 1	Inner Marginal 1				0.2963
		Lateral I 1	Inner Marginal 1				0.2492
		Outer Marginal 1	Lateral I 2				0.2224
		Outer Marginal 4	Lateral II 4				0.3558
		Outer Marginal 4	Inner Marginal 4				0.3181
		Outer Marginal 4	Inner Marginal 1				0.3216
		Outer Marginal 1	Lateral I 1				0.0876
		Outer Marginal 1	Lateral II 1				0.0876
		Outer Marginal 1	Inner Marginal 1				0.0740
		Outer Marginal 3	Lateral II 3				0.0740
		Lateral II 1	Inner Marginal 4				0.1547
		Lateral II 4	Inner Marginal 1				0.2154
		Lateral I 3	Inner Marginal 4				0.1390
		Lateral I 1	Inner Marginal 3				0.0367*
		Lateral II 1	Inner Marginal 3				0.0367*
		Outer Marginal 1	Lateral I 3				0.0724
		Lateral I 1	Inner Marginal 4				0.1264
		Outer Marginal 2	Lateral II 3				0.0513
		Lateral I 2	Inner Marginal 3				0.0216*
		Lateral II 4	Inner Marginal 4				0.1568
		Lateral I 2	Inner Marginal 4				0.0651
		Lateral I 3	Inner Marginal 3				0.0147*
		Lateral I 4	Inner Marginal 1				0.1262
		Outer Marginal 1	Inner Marginal 3				0.0058*
		Outer Marginal 1	Lateral II 3				0.0058*
		Lateral I 1	Inner Marginal 2				0.0068*
		Lateral I 2	Inner Marginal 2				0.0068*
		Lateral II 1	Inner Marginal 2				0.0068*
		Inner Marginal 4	Inner Marginal 3				0.0234*
		Lateral II 2	Inner Marginal 3				0.1409
		Lateral II 4	Inner Marginal 3				0.0431*
		Lateral I 3	Inner Marginal 2				0.0031*
		Outer Marginal 1	Inner Marginal 2				0.0015*
		Outer Marginal 4	Inner Marginal 3				0.0118*
		Inner Marginal 4	Inner Marginal 2				0.0023*
		Lateral I 4	Inner Marginal 4				0.0134*
		Outer Marginal 4	Outer Marginal 3				0.0056*
		Outer Marginal 1	Inner Marginal 4				0.0005*
		Lateral II 2	Inner Marginal 2				0.0139*
		Lateral II 4	Lateral II 3				0.0011*
		Outer Marginal 4	Lateral II 3				0.0011*
		Lateral II 4	Inner Marginal 2				0.0007*
		Lateral I 4	Inner Marginal 3				0.0039*
		Outer Marginal 4	Inner Marginal 2				0.0004*
		Outer Marginal 4	Outer Marginal 2				0.0005*
		Outer Marginal 1	Lateral II 4				0.0002*
		Lateral II 4	Lateral II 2				0.0012*
		Outer Marginal 1	Lateral I 4				0.0003*
		Outer Marginal 4	Lateral II 2				0.0005*
		Outer Marginal 1	Lateral II 2				0.0007*
		Lateral I 4	Inner Marginal 2				<.0001*
		Lateral II 2	Lateral I 4				<.0001*
		Lateral II 2	Inner Marginal 4				<.0001*
		Outer Marginal 2	Lateral I 4				0.0005*
		Outer Marginal 3	Lateral I 4				0.0008*
		Lateral II 4	Inner Marginal 4				0.0032*
		Lateral II 4	Lateral I 4				0.0108*
		Lateral I 3	Inner Marginal 4				0.0008*
		Outer Marginal 3	Inner Marginal 4				0.0008*
		Outer Marginal 2	Inner Marginal 4				0.0009*
		Lateral I 2	Inner Marginal 4				0.0037*
		Lateral II 3	Lateral I 4				0.0447*
		Lateral II 3	Inner Marginal 4				0.0071*
	**CI**	Lateral II 2	Lateral I 1	73.9170	15	<.0001*	0.0633
		Outer Marginal 2	Outer Marginal 1				0.0034*
		Outer Marginal 3	Outer Marginal 1				0.0104*
		Lateral I 1	Inner Marginal 4				0.0448*
		Lateral II 2	Lateral I 2				0.2160
		Lateral I 4	Inner Marginal 4				0.1724
		Lateral II 1	Lateral I 4				0.2725
		Lateral II 1	Inner Marginal 4				0.1114
		Outer Marginal 1	Inner Marginal 4				0.0904
		Lateral II 2	Lateral II 1				0.3146
		Outer Marginal 2	Lateral II 4				0.1726
		Lateral II 2	Inner Marginal 1				0.3588
		Outer Marginal 2	Lateral I 2				0.0552

		Outer Marginal 2	Lateral II 3				0.0552
		Outer Marginal 3	Lateral II 4				0.2160
		Lateral II 2	Inner Marginal 2				0.3157
		Outer Marginal 2	Lateral I 1				0.0828
		Lateral II 2	Lateral I 3				0.3667
		Lateral I 3	Lateral I 1				0.1432
		Outer Marginal 3	Lateral I 2				0.1432
		Outer Marginal 3	Lateral II 3				0.1417
		Outer Marginal 2	Inner Marginal 2				0.2195
		Outer Marginal 3	Lateral I 1				0.1931
		Outer Marginal 3	Lateral I 3				0.2491
		Lateral I 2	Lateral I 1				0.2101
		Outer Marginal 2	Lateral I 3				0.2833
		Outer Marginal 1	Lateral I 4				0.5794
		Outer Marginal 3	Inner Marginal 2				0.4166
		Outer Marginal 2	Inner Marginal 1				0.3662
		Outer Marginal 2	Lateral II 1				0.3662
		Lateral I 3	Lateral I 2				0.4118
		Inner Marginal 2	Inner Marginal 1				0.4740
		Inner Marginal 3	Inner Marginal 1				0.3725
		Lateral I 3	Inner Marginal 1				0.4928
		Outer Marginal 3	Inner Marginal 1				0.4928
		Outer Marginal 3	Lateral II 1				0.4928
		Outer Marginal 2	Lateral II 2				0.7262
		Lateral II 3	Lateral I 1				0.5309
		Outer Marginal 4	Inner Marginal 4				0.7097
		Lateral I 2	Inner Marginal 1				0.5510
		Lateral II 4	Lateral I 2				0.7411
		Inner Marginal 3	Inner Marginal 2				0.6700
		Lateral II 4	Lateral II 3				0.7726
		Lateral II 4	Lateral I 1				0.8365
		Lateral II 3	Inner Marginal 1				0.7656
		Lateral II 3	Lateral II 1				0.7656
		Lateral I 3	Inner Marginal 2				0.9538
		Lateral I 1	Inner Marginal 1				1.0000
		Outer Marginal 2	Inner Marginal 3				1.0000
		Outer Marginal 3	Lateral II 2				1.0000
		Lateral II 4	Inner Marginal 1				0.9554
		Lateral II 4	Lateral II 1				0.9554
		Lateral II 4	Lateral I 3				0.9467
		Lateral II 1	Lateral I 1				0.8793
		Lateral II 2	Inner Marginal 3				0.9408
		Lateral II 1	Inner Marginal 1				0.8248
		Outer Marginal 3	Outer Marginal 2				0.8301
		Lateral I 2	Inner Marginal 2				0.8260
		Outer Marginal 3	Inner Marginal 3				0.7758
		Outer Marginal 1	Lateral II 1				0.6445
		Lateral II 1	Lateral I 2				0.5510
		Lateral II 4	Inner Marginal 2				0.6905
		Lateral I 3	Inner Marginal 3				0.5064
		Lateral II 1	Lateral I 3				0.4928
		Outer Marginal 1	Inner Marginal 1				0.4928
		Lateral II 1	Inner Marginal 3				0.3725
		Lateral II 1	Inner Marginal 2				0.4740
		Lateral II 3	Lateral I 2				0.2963
		Lateral II 3	Inner Marginal 3				0.2683
		Outer Marginal 1	Lateral I 1				0.3265
		Lateral I 2	Inner Marginal 3				0.1761
		Lateral I 1	Inner Marginal 3				0.1099
		Lateral II 3	Inner Marginal 2				0.1834
		Lateral II 3	Lateral I 3				0.1432
		Lateral I 1	Inner Marginal 2				0.1238
		Outer Marginal 1	Lateral II 3				0.1038
		Outer Marginal 4	Lateral I 4				0.3151
		Lateral II 4	Inner Marginal 3				0.2770
		Outer Marginal 1	Lateral II 4				0.2161
		Outer Marginal 4	Outer Marginal 1				0.1499
		Lateral I 4	Lateral I 1				0.2208
		Outer Marginal 4	Lateral II 1				0.1975
		Inner Marginal 4	Inner Marginal 1				0.1114
		Lateral I 4	Inner Marginal 1				0.2725
		Outer Marginal 1	Inner Marginal 3				0.0176*
		Outer Marginal 1	Lateral I 2				0.0115*
		Outer Marginal 4	Inner Marginal 1				0.1304
		Lateral II 4	Lateral II 2				0.1074
		Outer Marginal 1	Lateral I 3				0.0039*
		Outer Marginal 1	Inner Marginal 2				0.0045*
		Outer Marginal 4	Lateral I 1				0.0315*
		Lateral II 3	Lateral II 2				0.0631
		Inner Marginal 4	Inner Marginal 3				0.0062*
		Outer Marginal 4	Lateral II 4				0.0088*
		Outer Marginal 4	Lateral II 3				0.0033*
		Inner Marginal 4	Inner Marginal 2				0.0004*
		Outer Marginal 4	Inner Marginal 3				0.0029*
		Lateral I 4	Inner Marginal 3				0.0094*
		Lateral I 4	Lateral I 2				0.0059*
		Outer Marginal 4	Lateral I 2				0.0011*
		Outer Marginal 4	Outer Marginal 3				0.0006*
		Outer Marginal 4	Outer Marginal 2				0.0005*
		Lateral I 4	Inner Marginal 2				0.0010*
		Outer Marginal 4	Lateral I 3				0.0002*
		Lateral I 4	Lateral I 3				0.0009*
		Outer Marginal 4	Inner Marginal 2				<.0001*
		Outer Marginal 1	Lateral II 2				0.0008*

		Outer Marginal 4	Lateral II 2				<.0001*
	K	Outer Marginal 2	Lateral II 2	4.3690	3	0.2243	0.3711
		Lateral II 2	Inner Marginal 2				0.5408
		Inner Marginal 3	Inner Marginal 2				0.5403
		Outer Marginal 2	Inner Marginal 2				0.5403
		Outer Marginal 2	Inner Marginal 3				1.0000
		Lateral II 2	Inner Marginal 3				0.1797
	Ca	Lateral II 2	Lateral I 1	65.0009	15	<.0001*	0.0137*
		Lateral I 4	Lateral I 2				0.0031*
		Lateral I 4	Lateral I 1				0.0059*
		Lateral II 4	Lateral I 2				0.0011*
		Lateral II 2	Lateral I 2				0.0141*
		Lateral II 4	Inner Marginal 4				0.0012*
		Lateral II 4	Lateral I 1				0.0029*
		Lateral II 4	Lateral I 3				0.0015*
		Outer Marginal 4	Outer Marginal 1				0.0017*
		Lateral II 4	Lateral I 4				0.0108*
		Outer Marginal 2	Lateral II 2				0.0233*
		Lateral II 4	Lateral II 3				0.0057*
		Lateral II 4	Lateral II 2				0.0211*
		Lateral II 4	Lateral II 1				0.0094*
		Lateral I 4	Lateral I 3				0.0160*
		Outer Marginal 2	Lateral I 4				0.0186*
		Outer Marginal 4	Lateral I 2				0.0104*
		Lateral II 4	Inner Marginal 1				0.0118*
		Outer Marginal 4	Lateral I 1				0.0159*
		Lateral II 2	Lateral II 1				0.0758
		Outer Marginal 2	Inner Marginal 4				0.0035*
		Outer Marginal 2	Lateral I 3				0.0022*
		Outer Marginal 2	Outer Marginal 1				0.0022*
		Outer Marginal 3	Outer Marginal 1				0.0021*
		Lateral II 2	Lateral I 3				0.0933
		Outer Marginal 4	Lateral I 3				0.0382*
		Outer Marginal 2	Inner Marginal 2				0.0065*
		Outer Marginal 4	Lateral II 1				0.0653
		Lateral I 4	Inner Marginal 4				0.0794
		Lateral I 4	Inner Marginal 1				0.1100
		Outer Marginal 2	Inner Marginal 1				0.0058*
		Outer Marginal 2	Lateral I 2				0.0058*
		Outer Marginal 2	Lateral II 3				0.0057*
		Outer Marginal 3	Lateral I 2				0.0057*
		Lateral II 4	Inner Marginal 2				0.0617
		Outer Marginal 3	Lateral I 3				0.0105*
		Outer Marginal 2	Lateral I 1				0.0106*
		Outer Marginal 2	Lateral II 1				0.0107*
		Outer Marginal 3	Lateral I 1				0.0104*
		Outer Marginal 3	Lateral II 3				0.0145*
		Outer Marginal 4	Lateral II 3				0.1071
		Outer Marginal 3	Lateral II 2				0.1936
		Outer Marginal 3	Lateral II 1				0.0294*
		Lateral II 4	Inner Marginal 3				0.1729
		Outer Marginal 4	Inner Marginal 1				0.1729
		Outer Marginal 2	Inner Marginal 3				0.0416*
		Lateral II 2	Inner Marginal 4				0.2752
		Outer Marginal 3	Inner Marginal 4				0.1386
		Lateral II 3	Lateral I 1				0.0358*
		Outer Marginal 3	Inner Marginal 1				0.0735
		Lateral II 2	Inner Marginal 1				0.3861
		Lateral II 3	Lateral I 2				0.0593
		Outer Marginal 3	Inner Marginal 2				0.1626
		Outer Marginal 4	Inner Marginal 4				0.3174
		Lateral I 3	Lateral I 1				0.1839
		Inner Marginal 2	Inner Marginal 1				0.2716
		Inner Marginal 3	Inner Marginal 1				0.2087
		Lateral I 3	Lateral I 2				0.2523
		Inner Marginal 3	Inner Marginal 2				0.3035
		Lateral II 3	Lateral II 1				0.2683
		Lateral I 4	Inner Marginal 2				0.5930
		Lateral II 3	Lateral I 3				0.5152
		Inner Marginal 4	Inner Marginal 1				0.6348
		Outer Marginal 3	Lateral I 4				0.8899
		Outer Marginal 4	Lateral II 2				0.9011
		Lateral I 2	Lateral I 1				0.8049
		Lateral II 3	Inner Marginal 4				0.9579
		Lateral II 1	Lateral I 1				1.0000
		Lateral II 3	Inner Marginal 1				1.0000
		Outer Marginal 1	Lateral II 1				0.9244
		Lateral II 1	Lateral I 2				0.9025
		Outer Marginal 3	Inner Marginal 3				0.8699
		Outer Marginal 4	Inner Marginal 2				0.8783
		Outer Marginal 2	Lateral II 4				0.8673
		Lateral I 3	Inner Marginal 1				0.6842
		Outer Marginal 1	Lateral I 1				0.6335
		Lateral I 4	Inner Marginal 3				0.7083
		Lateral II 1	Lateral I 3				0.5083
		Lateral II 2	Inner Marginal 2				0.6948
		Lateral I 3	Inner Marginal 4				0.5254
		Outer Marginal 1	Lateral I 2				0.3709
		Lateral II 2	Lateral I 4				0.6121
		Lateral I 2	Inner Marginal 1				0.2963
		Outer Marginal 4	Outer Marginal 3				0.4827
		Lateral II 1	Inner Marginal 1				0.2187
		Lateral I 1	Inner Marginal 1				0.1761
		Inner Marginal 4	Inner Marginal 2				0.2627

		Lateral II 1	Inner Marginal 2				0.1060
		Outer Marginal 4	Lateral I 4				0.2783
		Lateral II 1	Inner Marginal 3				0.0365*
		Outer Marginal 1	Inner Marginal 1				0.0614
		Outer Marginal 4	Inner Marginal 3				0.2003
		Inner Marginal 4	Inner Marginal 3				0.1257
		Lateral I 3	Inner Marginal 2				0.0726
		Lateral II 1	Inner Marginal 4				0.1290
		Outer Marginal 3	Lateral II 4				0.1707
		Lateral I 1	Inner Marginal 3				0.0189*
		Lateral II 2	Inner Marginal 3				0.2983
		Lateral II 3	Inner Marginal 2				0.0475*
		Lateral I 2	Inner Marginal 4				0.1015
		Lateral II 3	Inner Marginal 3				0.0208*
		Outer Marginal 1	Lateral I 3				0.0474*
		Lateral II 3	Lateral II 2				0.2597
		Lateral I 2	Inner Marginal 3				0.0119*
		Lateral I 3	Inner Marginal 3				0.0147*
		Lateral I 1	Inner Marginal 4				0.0592
		Outer Marginal 1	Lateral II 3				0.0092*
		Lateral I 1	Inner Marginal 2				0.0082*
		Outer Marginal 1	Inner Marginal 3				0.0057*
		Outer Marginal 3	Outer Marginal 2				0.0059*
		Lateral I 2	Inner Marginal 2				0.0043*
		Lateral II 3	Lateral I 4				0.0614
		Outer Marginal 1	Inner Marginal 4				0.0086*
		Outer Marginal 1	Inner Marginal 2				0.0014*
		Lateral II 1	Lateral I 4				0.0362*
		Outer Marginal 4	Lateral II 4				0.0063*
		Outer Marginal 4	Outer Marginal 2				0.0019*
		Outer Marginal 1	Lateral II 4				0.0002*
		Outer Marginal 1	Lateral I 4				0.0004*
		Outer Marginal 1	Lateral II 2				0.0013*
		Lateral II 2	Lateral II 1				0.0514
	**Fe**	Lateral II 4	Lateral II 1	5.0775	2	0.0790	0.0654
		Lateral II 4	Lateral II 2				0.3106
		Lateral II 2	Lateral I 4				<.0001*
		Lateral II 2	Inner Marginal 4				<.0001*
		Lateral II 2	Lateral I 1				0.0009*
		Lateral II 2	Lateral II 1				0.0009*
		Lateral II 4	Lateral I 4				<.0001*
		Outer Marginal 3	Lateral I 4				0.0002*
		Lateral II 4	Inner Marginal 4				<.0001*
		Lateral II 2	Inner Marginal 1				0.0029*
		Lateral II 3	Lateral I 4				0.0008*
		Lateral II 2	Lateral I 2				0.0043*
		Lateral II 2	Lateral I 3				0.0076*
		Lateral II 4	Inner Marginal 1				0.0011*
		Lateral II 4	Lateral I 1				0.0011*
		Lateral II 4	Lateral II 1				0.0011*
		Lateral II 4	Lateral I 2				0.0020*
		Lateral II 4	Lateral I 3				0.0019*
		Lateral II 3	Lateral II 2				0.0225*
		Outer Marginal 3	Inner Marginal 4				0.0004*
		Lateral II 2	Inner Marginal 2				0.0177*
		Outer Marginal 2	Lateral I 4				0.0118*
		Lateral II 3	Inner Marginal 4				0.0019*
		Outer Marginal 2	Inner Marginal 4				0.0020*
		Outer Marginal 4	Outer Marginal 1				0.0075*
		Lateral II 2	Inner Marginal 3				0.0734
		Lateral I 4	Lateral I 1				0.0488*
		Outer Marginal 2	Outer Marginal 1				0.0021*
		Outer Marginal 3	Lateral I 3				0.0022*
		Outer Marginal 3	Outer Marginal 1				0.0022*
	**All elements**	Lateral I 3	Inner Marginal 4	105.9252	15	<.0001*	0.0126*
		Lateral II 3	Inner Marginal 2				0.0043*
		Outer Marginal 4	Lateral I 1				0.0575
		Inner Marginal 2	Inner Marginal 1				0.0068*
		Lateral II 3	Lateral I 3				0.0058*
		Outer Marginal 2	Inner Marginal 1				0.0057*
		Outer Marginal 2	Lateral I 1				0.0057*
		Outer Marginal 2	Lateral II 1				0.0056*
		Outer Marginal 3	Inner Marginal 1				0.0058*
		Outer Marginal 3	Lateral I 1				0.0058*
		Outer Marginal 3	Lateral I 2				0.0058*
		Outer Marginal 3	Lateral II 1				0.0057*
		Lateral I 3	Lateral I 1				0.0094*
		Outer Marginal 2	Lateral I 2				0.0092*
		Outer Marginal 2	Lateral I 3				0.0213*
		Outer Marginal 4	Lateral II 1				0.1265
		Inner Marginal 3	Inner Marginal 1				0.0122*
		Lateral II 3	Inner Marginal 1				0.0122*
		Lateral II 3	Inner Marginal 3				0.0122*
		Lateral II 3	Lateral I 1				0.0122*
		Lateral II 3	Lateral I 2				0.0122*
		Lateral II 3	Lateral II 1				0.0119*
		Lateral I 3	Inner Marginal 1				0.0230*
		Outer Marginal 3	Inner Marginal 2				0.0562
		Lateral I 2	Lateral I 1				0.0601
		Lateral I 3	Lateral I 2				0.1044
		Lateral I 4	Inner Marginal 4				0.3402
		Lateral II 4	Inner Marginal 2				0.3123
		Outer Marginal 4	Inner Marginal 1				0.3423
		Lateral I 4	Inner Marginal 1				0.4148

		Outer Marginal 3	Outer Marginal 2				0.2496
		Outer Marginal 4	Inner Marginal 4				0.4299
		Lateral I 4	Lateral I 2				0.6588
		Lateral I 2	Inner Marginal 1				0.4034
		Lateral II 1	Lateral I 1				0.4020
		Lateral I 2	Inner Marginal 4				0.5618
		Inner Marginal 3	Inner Marginal 2				0.6084
		Lateral II 4	Inner Marginal 3				0.7726
		Inner Marginal 4	Inner Marginal 1				0.7920
		Outer Marginal 3	Inner Marginal 3				1.0000
		Outer Marginal 4	Lateral I 2				1.0000
		Outer Marginal 2	Inner Marginal 2				0.9538
		Outer Marginal 2	Lateral II 4				0.7132
		Outer Marginal 3	Lateral II 4				0.7133
		Lateral II 1	Inner Marginal 1				0.4020
		Lateral I 4	Lateral I 3				0.6381
		Outer Marginal 2	Inner Marginal 3				0.4160
		Outer Marginal 1	Lateral I 1				0.3709
		Lateral I 1	Inner Marginal 1				0.1437
		Lateral II 1	Lateral I 2				0.0937
		Lateral II 1	Inner Marginal 4				0.2249
		Outer Marginal 4	Lateral I 4				0.3395
		Outer Marginal 1	Lateral II 1				0.0509
		Lateral I 1	Inner Marginal 4				0.1021
		Outer Marginal 1	Inner Marginal 1				0.0348*
		Lateral I 1	Inner Marginal 3				0.0122*
		Lateral I 2	Inner Marginal 3				0.0122*
		Lateral II 1	Inner Marginal 3				0.0119*
		Outer Marginal 2	Lateral II 3				0.0228*
		Lateral II 1	Lateral I 4				0.1636
		Outer Marginal 1	Lateral I 2				0.0149*
		Lateral II 4	Lateral II 2				0.1550
		Outer Marginal 2	Lateral II 2				0.1633
		Lateral II 1	Lateral I 3				0.0092*
		Lateral I 2	Inner Marginal 2				0.0104*
		Lateral I 3	Inner Marginal 2				0.0128*
		Lateral I 3	Inner Marginal 3				0.0058*
		Outer Marginal 1	Inner Marginal 3				0.0058*
		Outer Marginal 1	Lateral II 3				0.0058*
		Outer Marginal 3	Lateral II 3				0.0058*
		Outer Marginal 1	Inner Marginal 4				0.0250*
		Lateral I 1	Inner Marginal 2				0.0043*
		Lateral II 1	Inner Marginal 2				0.0043*
		Outer Marginal 4	Lateral I 3				0.0353*
		Outer Marginal 1	Lateral I 3				0.0033*
		Outer Marginal 3	Lateral II 2				0.0689
		Outer Marginal 1	Inner Marginal 2				0.0015*
		Inner Marginal 4	Inner Marginal 3				0.0019*
		Inner Marginal 4	Inner Marginal 2				0.0008*
		Outer Marginal 1	Lateral I 4				0.0052*
		Lateral II 4	Lateral II 3				0.0020*
		Outer Marginal 4	Inner Marginal 2				0.0011*
		Outer Marginal 4	Inner Marginal 3				0.0011*
		Outer Marginal 4	Lateral II 3				0.0011*
		Outer Marginal 4	Outer Marginal 2				0.0005*
		Outer Marginal 4	Outer Marginal 3				0.0002*
		Outer Marginal 1	Lateral II 4				0.0002*
		Lateral I 4	Inner Marginal 2				0.0009*
		Lateral I 4	Inner Marginal 3				0.0013*
		Outer Marginal 1	Lateral II 2				<.0001*
		Outer Marginal 4	Lateral II 4				<.0001*
		Outer Marginal 4	Lateral II 2				<.0001*
